# Supplementary material for: A cool temperature–induced ubiquitination-controlled transcription factor promotes starch degradation and ripening in kiwifruit
Source: Plant Commun. 2026 Jan 21;7(7):101736. doi: 10.1016/j.xplc.2026.101736 (PMC13370233; doi:10.1016/j.xplc.2026.101736)
Supplement: Document S1. Supplemental Figures 1–22 and Supplemental Tables 1 and 2 [file mmc1.pdf]

Plant Communications, Volume 7

Supplemental information

A cool temperature–induced ubiquitination-controlled transcription factor promotes starch degradation and ripening in kiwifruit

Ang Li, Yunhe Meng, Xiaoya Chen, Zhebin Zeng, Zhidan Zhao, Tiantian Li, Gang Ding, Ross G. Atkinson, Yue Huang, Yunjiang Cheng, Xiuxin Deng, and Yunliu Zeng

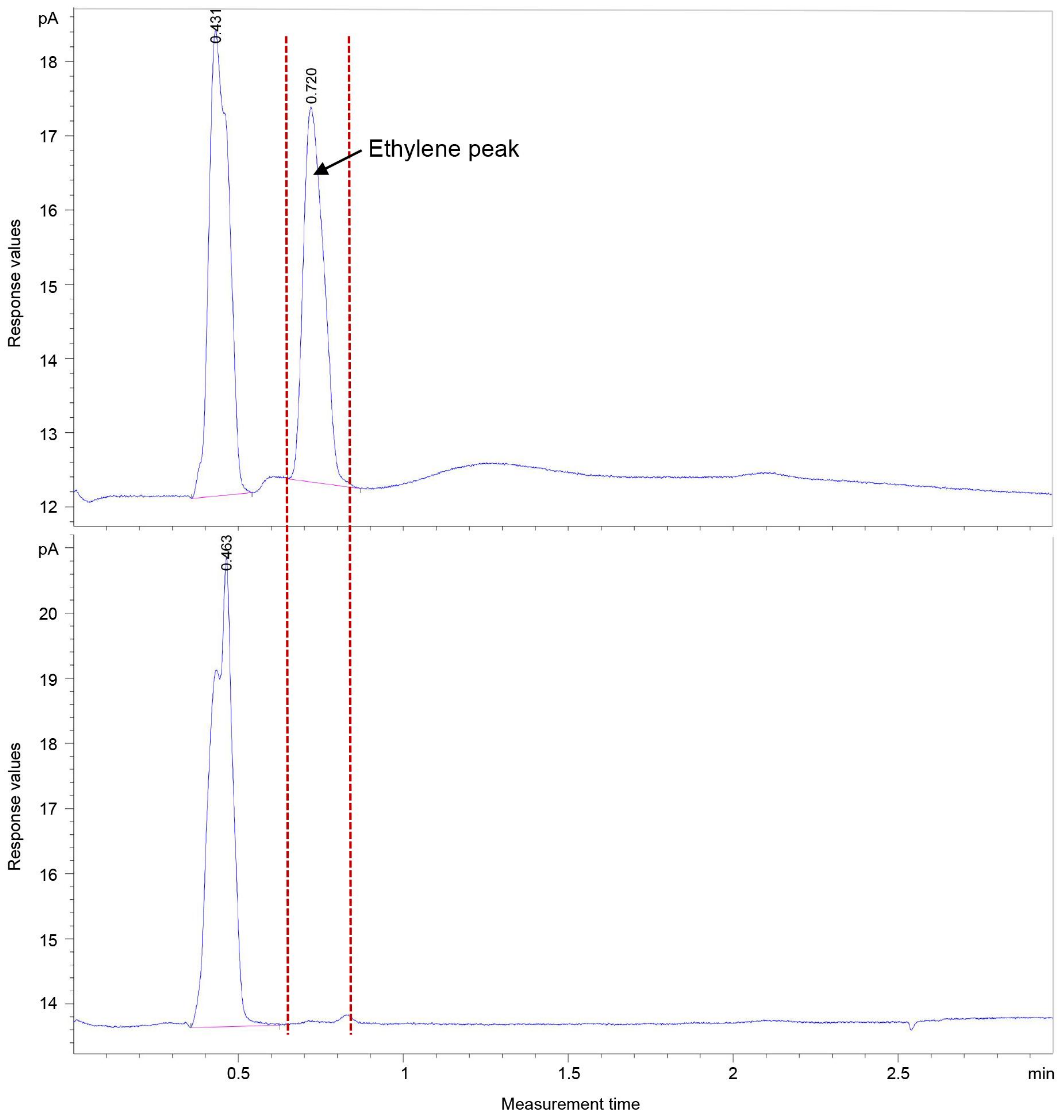

Supplementary Fig. 1 Ethylene analysis by gas chromatography in kiwifruit during storage. Representative chromatogram traces confirm a detectable ethylene peak in fruit treated with exogenous ethylene, but no ethylene peak in fruit stored at cool temperature (CT).

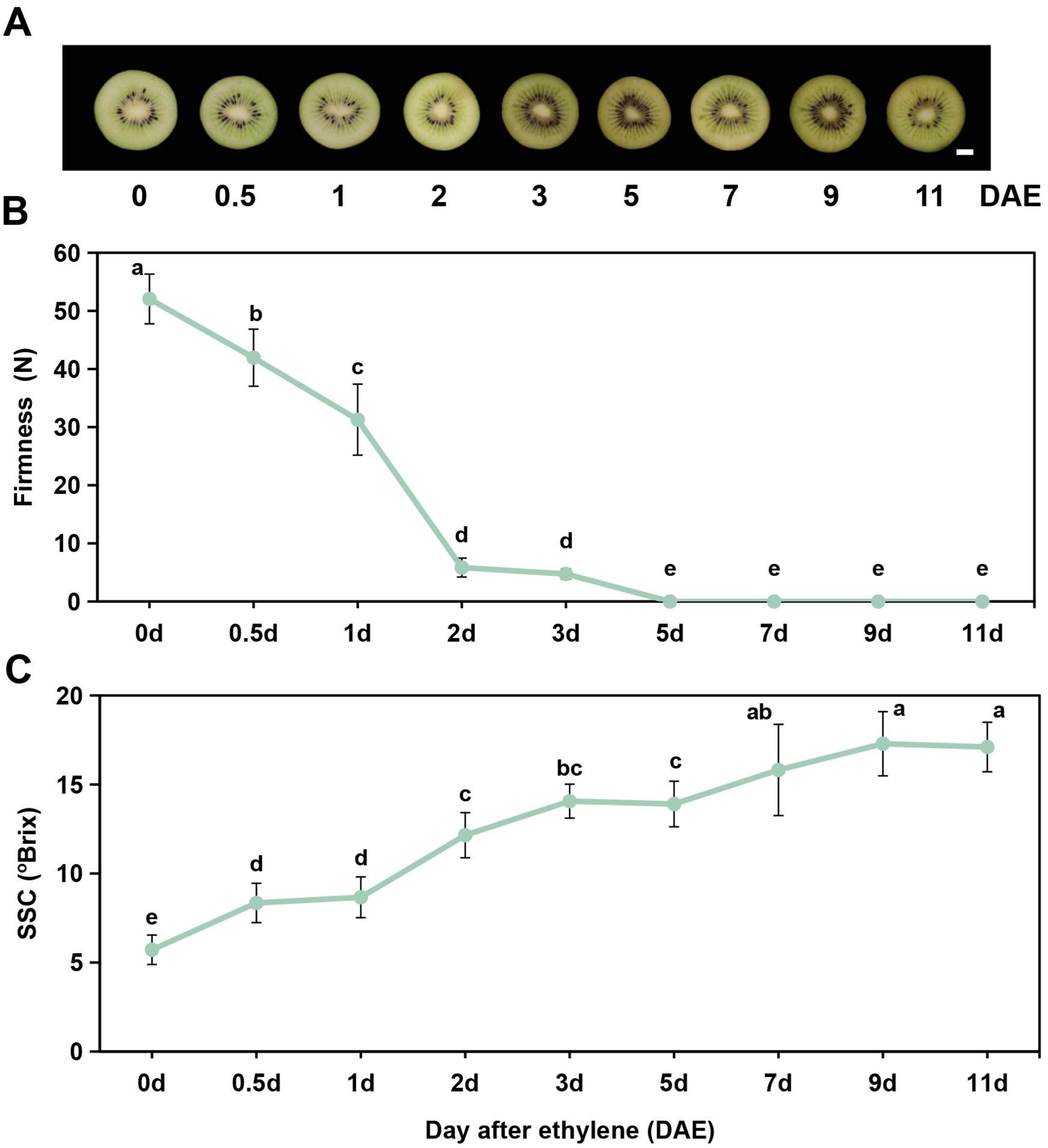

Supplementary Fig. 2 Changes in appearance, firmness and soluble solids content in 'Jintang No.3' kiwifruit treated with ethylene.
Alterations in appearance (A), firmness (B), and soluble solids content (C) of 'Jintang No.3' kiwifruit in 0–11 d following ethylene treatment (DAE). Scale bar represents 1 cm. Data are mean ± SE (n ≥ 3). Statistically significant differences (*P*<0.05) are denoted by lowercase letters.

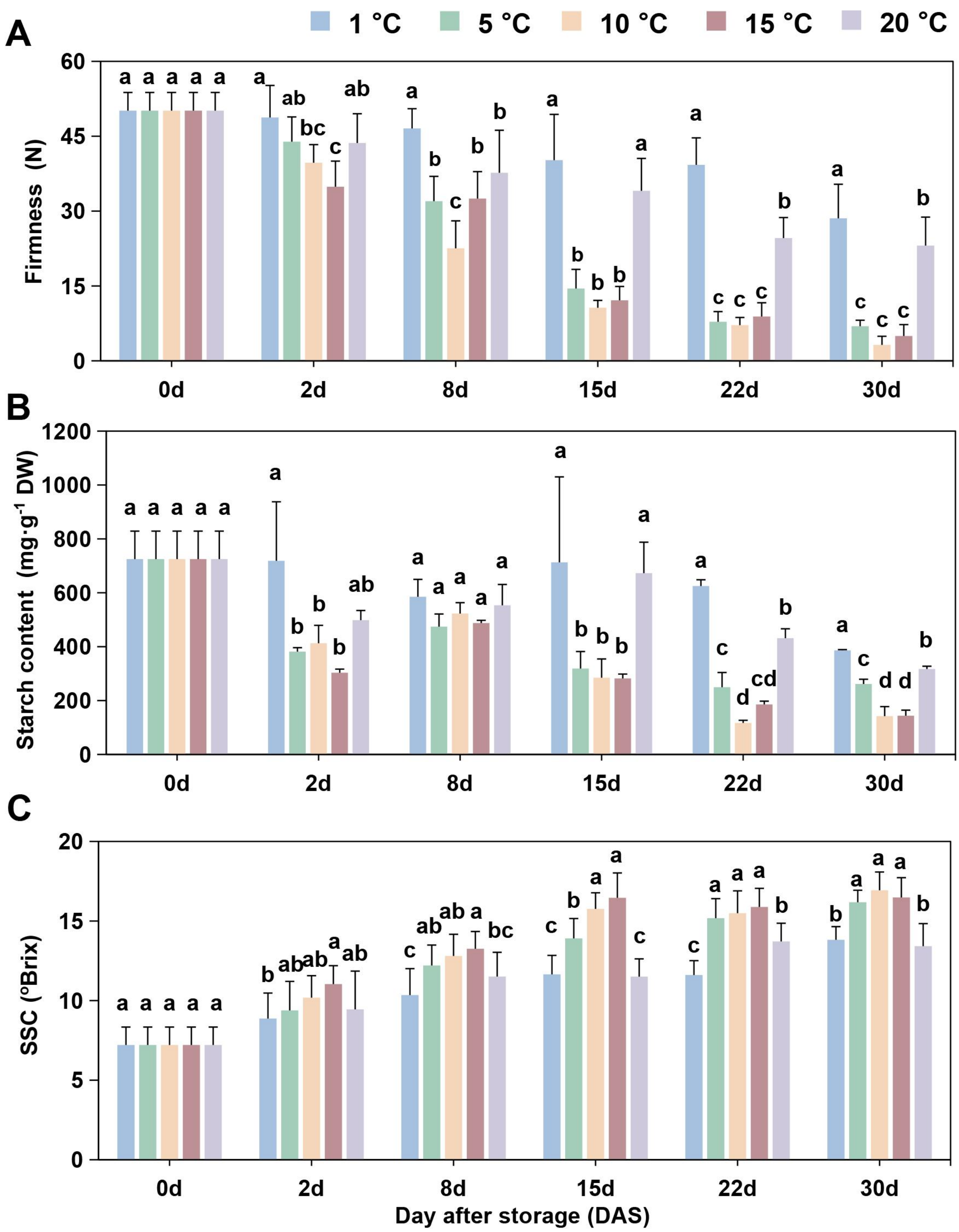

Supplementary Fig. 3 Changes in firmness, starch content and soluble solids content 'Hongyang' kiwifruit during storage at different temperatures.

Variations in firmness (A), starch content (B), and soluble solids content (C) of 'Hongyang' kiwifruit during storage at 1°C, 5°C, 10°C, 15°C, and 20°C. Different colors represent the respective storage temperatures. Data are mean ± SE (n ≥ 3). Statistically significant differences (*P*<0.05) are denoted by lowercase letters.

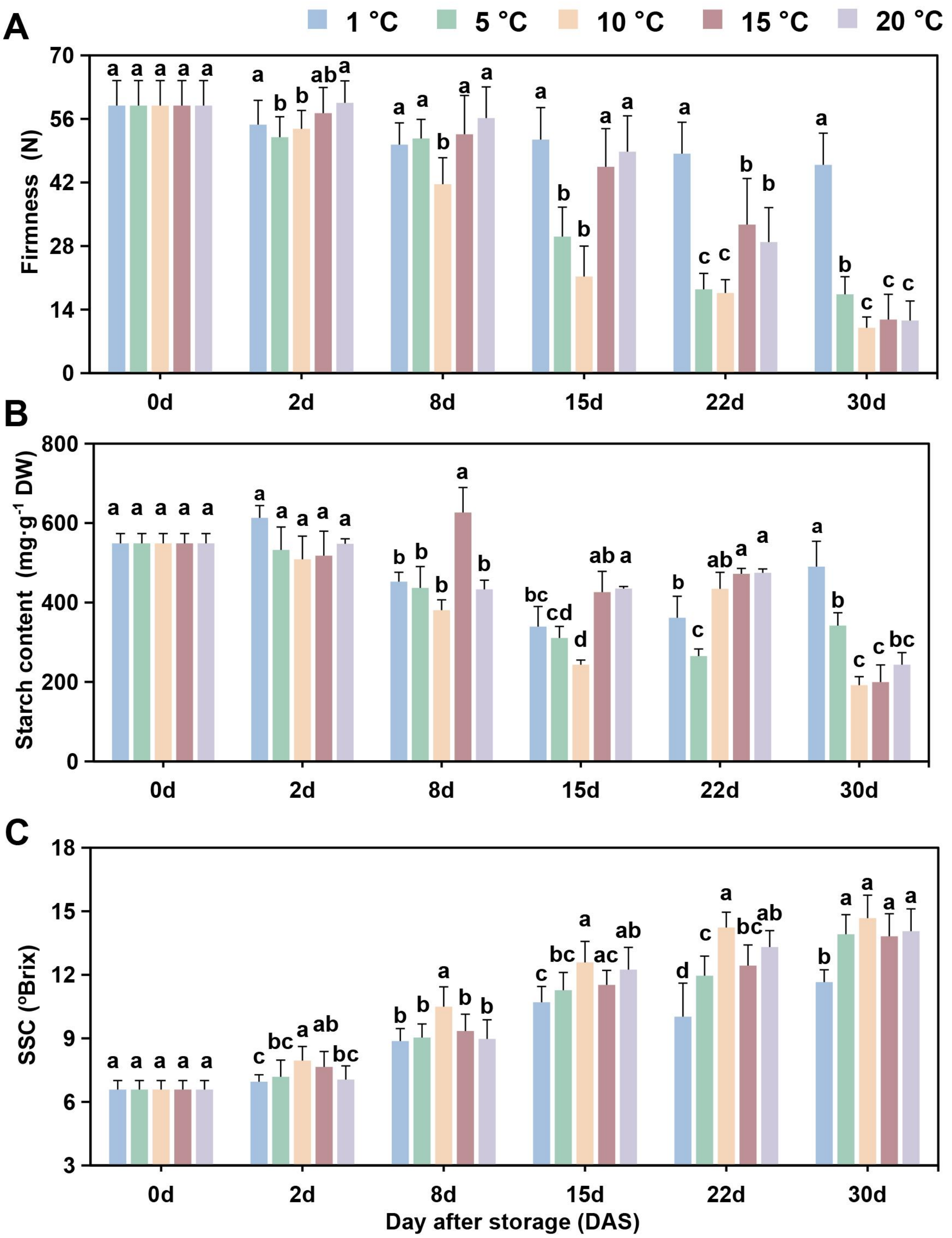

Supplementary Fig. 4 Changes in firmness, starch content and soluble solids content in 'Cuixiang' kiwifruit during storage at different temperatures.
Variations in starch content (A), soluble solids content (B), and firmness (C) of 'Cuixiang' kiwifruit during storage at 1°C, 5°C, 10°C, 15°C, and 20°C. Different colors represent the respective storage temperatures. Data are mean ± SE (n ≥ 3). Statistically significant differences (*P*<0.05) are denoted by lowercase letters.

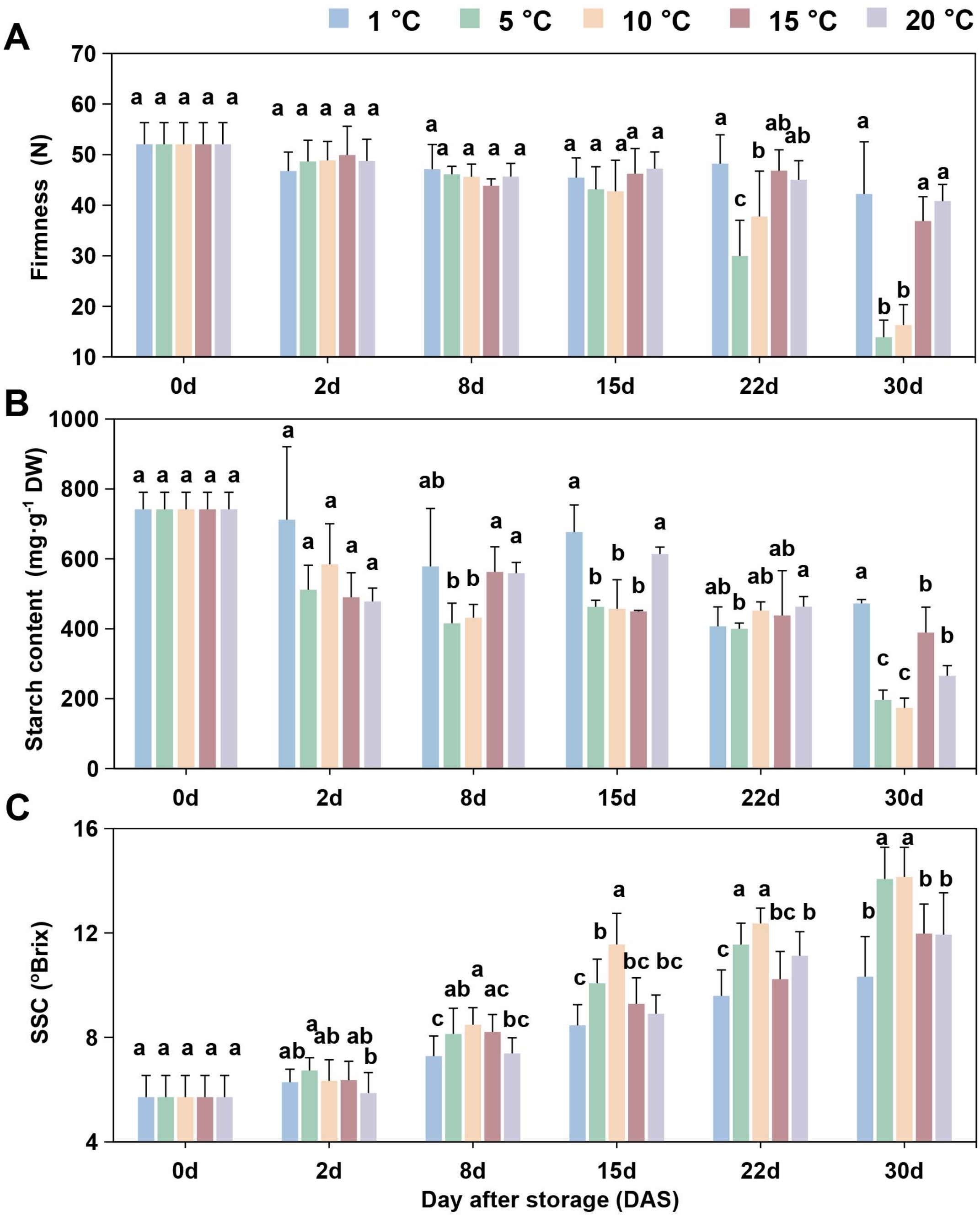

Supplementary Fig. 5 Changes in firmness, starch content, and soluble solids content in 'Jintang No.3' kiwifruit during storage at different temperatures. Changes in firmness (A), starch content (B), and soluble solids content (C) of 'Jintang No.3' kiwifruit during storage at 1°C, 5°C, 10°C, 15°C, and 20°C. Different colors represent the respective storage temperatures. Data are mean ± SE (n ≥ 3). Significant differences (*P*<0.05) are denoted by lowercase letters.

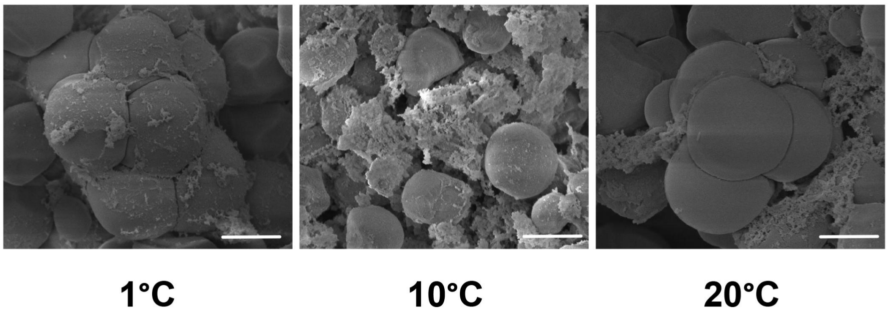

Supplementary Fig. 6 The ultrastructure of starch grain in kiwifruit under different temperature during storage

Starch grains were imaged by scanning electron microscopy at 22 day after storage (DAS) following storage at 1°C, 10°C, and 20°C in 'Hongyang' fruit. Scale = 5 μm.

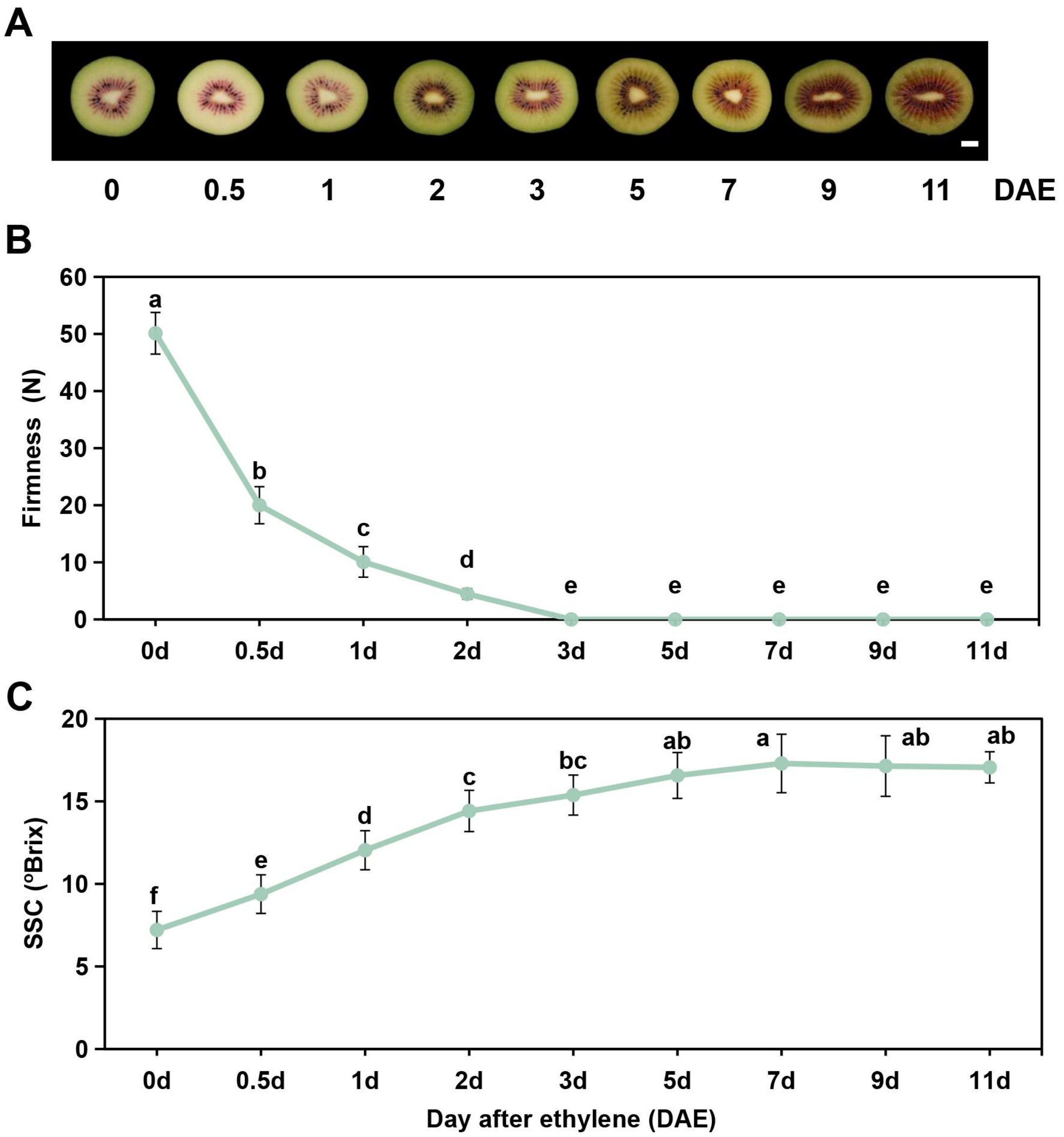

Supplementary Fig. 7 Changes in appearance, firmness and soluble solids content in 'Hongyang' kiwifruit treated with ethylene.

Changes in appearance (A), soluble solids content (B), and firmness (C) of 'Hongyang' kiwifruit in 0–11 d after ethylene treatment (DAE). Data are mean ± SE (n ≥ 3). Scale bar represents 1 cm. Significant differences (*P*<0.05) are denoted by lowercase letters.

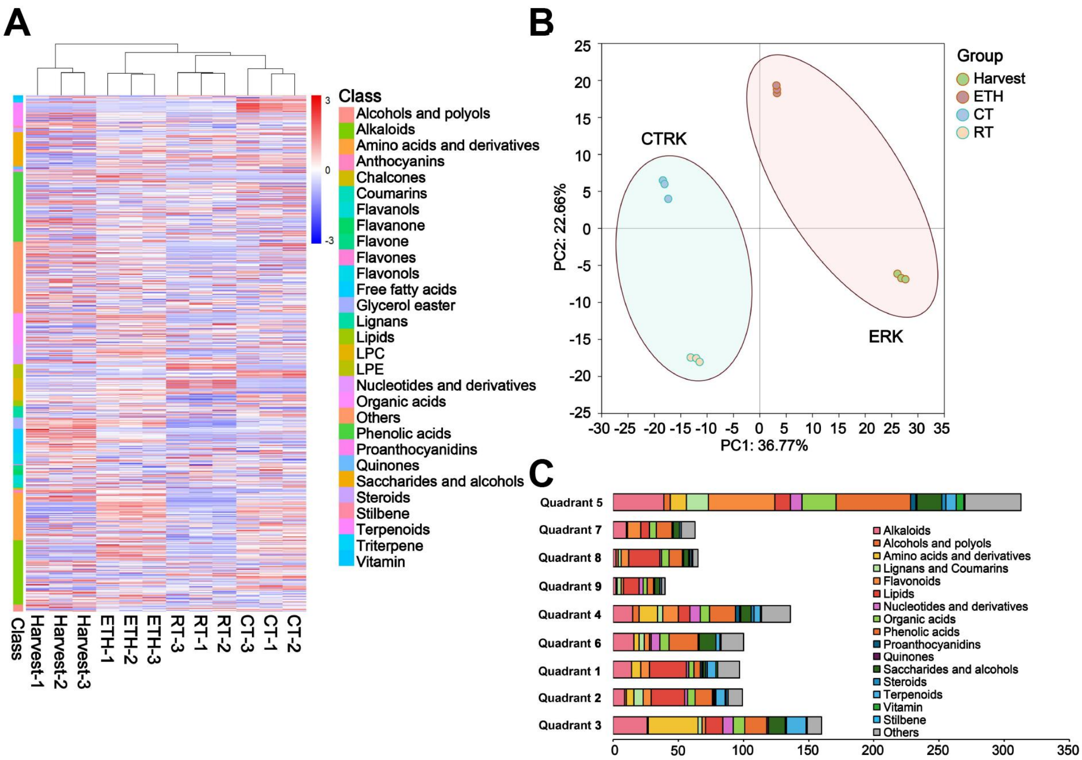

Supplementary Fig. 8 Metabolome analysis of cool-temperature and ethylene treated kiwifruits.

A) A summary of the metabolome data set for cool-temperature (CT) induced ripened kiwifruits (CTRKs) and ethylene-induced ripened kiwifruits (ERKs). Cluster analysis of metabolomes with Z-scores standardized to −3 to 3.

B) Principal component analysis (PCA) of metabolomes in cool-temperature induced ripened kiwifruits (CTRKs) and ethylene-induced ripened kiwifruits (ERKs).

C) Statistics of metabolite class categories in the nine quadrants, with color marks indicating various enrichments in metabolic pathways. A total of 1,073 metabolites were statistically analyzed, with a coefficient of variation >0.15 and average abundance >1. The nine quadrants (1−9) represent different accumulation patterns of metabolites under cool temperature and ethylene treatment, as shown in Fig. 1C. 1, increased at CT but decreased under ethylene; 2, increased at CT and stable under ethylene; 3, increased at both CT and ethylene; 4, stable at CT but decreased under ethylene; 5, unclassified; 6, stable at CT but increased under ethylene; 7, decreased at both CT and ethylene; 8, decreased at CT but stable under ethylene; 9, decreased at CT but increased under ethylene. The X-axis displays the number of metabolites in each quadrant, with 97, 99, 160, 136, 313, 100, 63, 65, and 40 metabolites in quadrants 1-9, respectively. Lipids were mainly enriched in quadrants 1, 2, 8, and 9, accounting for 28.9%, 26.3%, 37.0%, and 30.0%, respectively. Quadrant 3 was primarily enriched with amino acids and derivatives, making up 23.75%. Phenolic acids were predominantly found in quadrants 4, 5, 6, and 7, with respective proportions of 14.7%, 18.2%, 22.0%, and 19.1%.

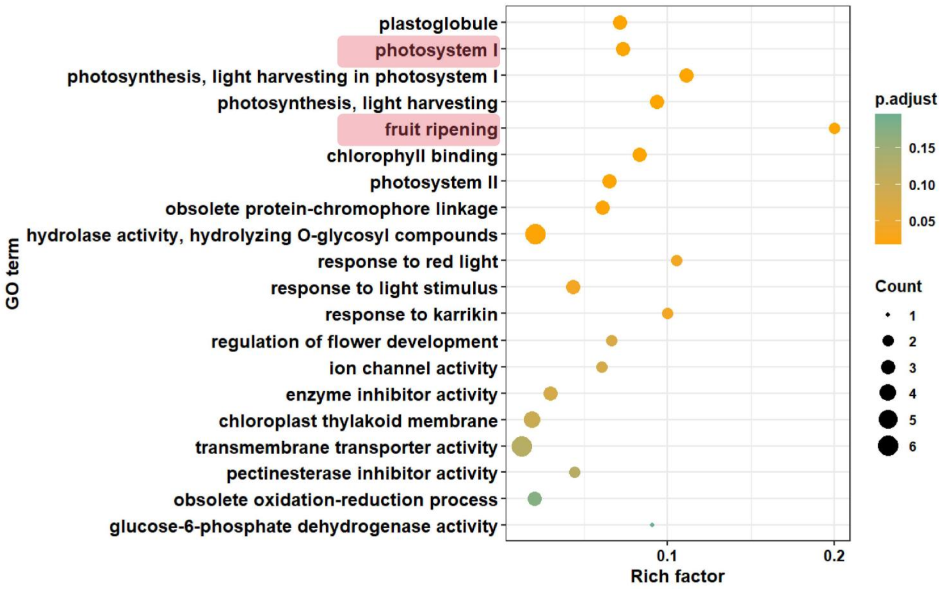

Supplementary Fig. 9 Gene ontology analysis.

Gene ontology (GO) analysis of genes specifically induced by cool temperature (CT) in 'Jingtang No.3' and 'Hongyang' kiwifruit. A total of 133 genes were analyzed. The X-axis shows the proportion of differentially expressed genes in each pathway relative to the total number of genes. The Y-axis represents the GO terms, which include molecular function, cellular component, and biological process. The color indicates the *P*-value, and the size of the dots reflects the number of differentially expressed genes in each category. Fruit ripening and photosynthesis, including processes like light harvesting in photosystem I and light harvesting in photosynthesis, displayed the highest enrichment, with factor values of 0.20 and 0.21, respectively.

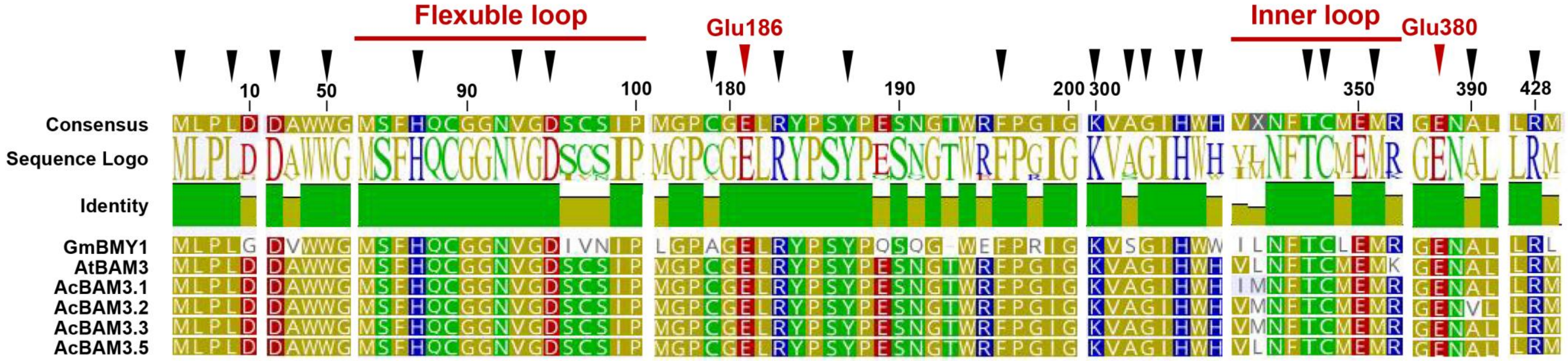

Supplementary Fig. 10 Alignment of core glucosyl hydrolase domains in β-amylase genes. The alignment of AcBAM3.1, AcBAM3.2, AcBAM3.3, and AcBAM3.5 proteins, along with the Arabidopsis AtBAM3 protein and the soybean GmBMY1 protein was performed using the ClustalW sequence alignment program and analyzed with Geneious (R11 version). Color shading indicates conserved substitutions, while unshaded residues are not conserved. The bar graph illustrates sequence conservation, with green bars indicating high conservation and short brown bars indicating low conservation. Black arrowheads denote substrate binding residues, and red arrowheads indicate the catalytic residues Glu186 and Glu380. Red solid lines above the sequences mark the residues forming the flexible and inner loops

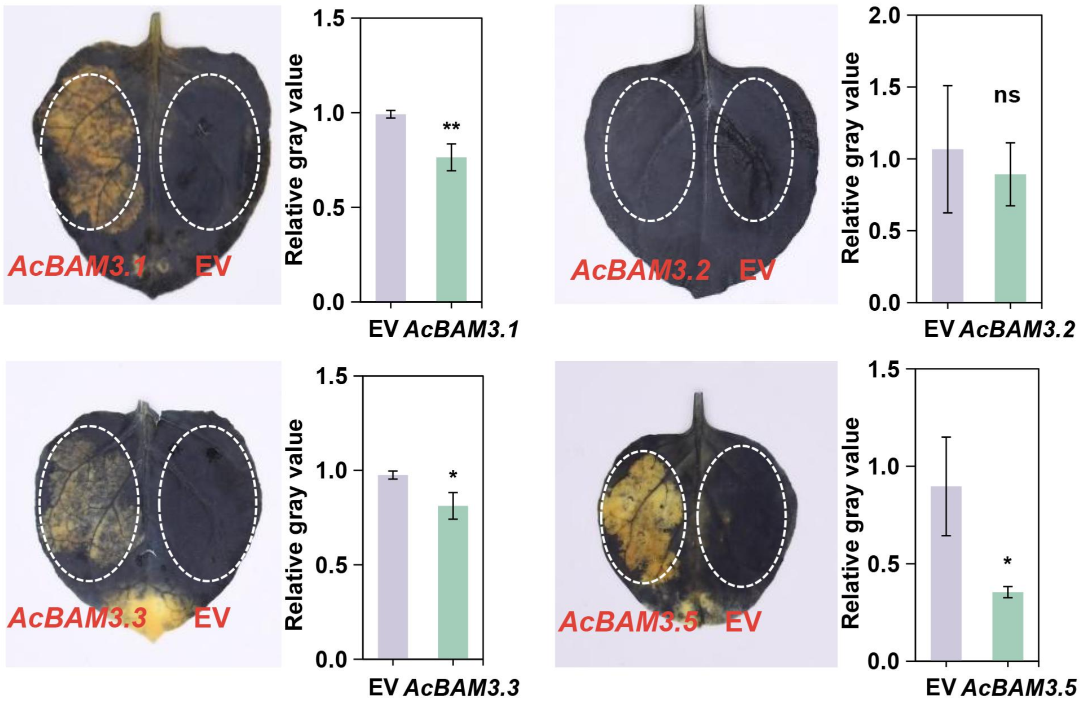

Supplementary Fig. 11 I-KI staining analysis of *N. benthamiana* leaves, with *AcBAM3* genes driven by the 35S promoter (left) and control infiltrated with empty vector (EV; right).

Starch degradation areas are circled with white dashed lines, and starch content is represented as grayscale value. ImageJ was used to calculate the grayscale value of the infiltrated area. Data are means ± SE (n = 3). Asterisks indicate significant differences between dehydrated samples and control at the same time point by Student's *t*-test (*, $P<0.05$; **, $P<0.01$); ns = not significant.

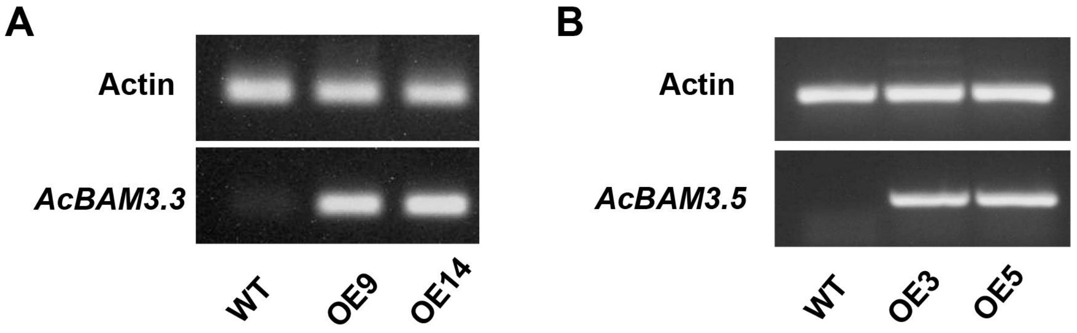

Supplementary Fig. 12 Semi-quantitative expression analysis of *AcBAM3.3*-OE (A) and *AcBAM3.5*-OE (B) tomato fruits. Primers were shown in Spplementary Table 2.

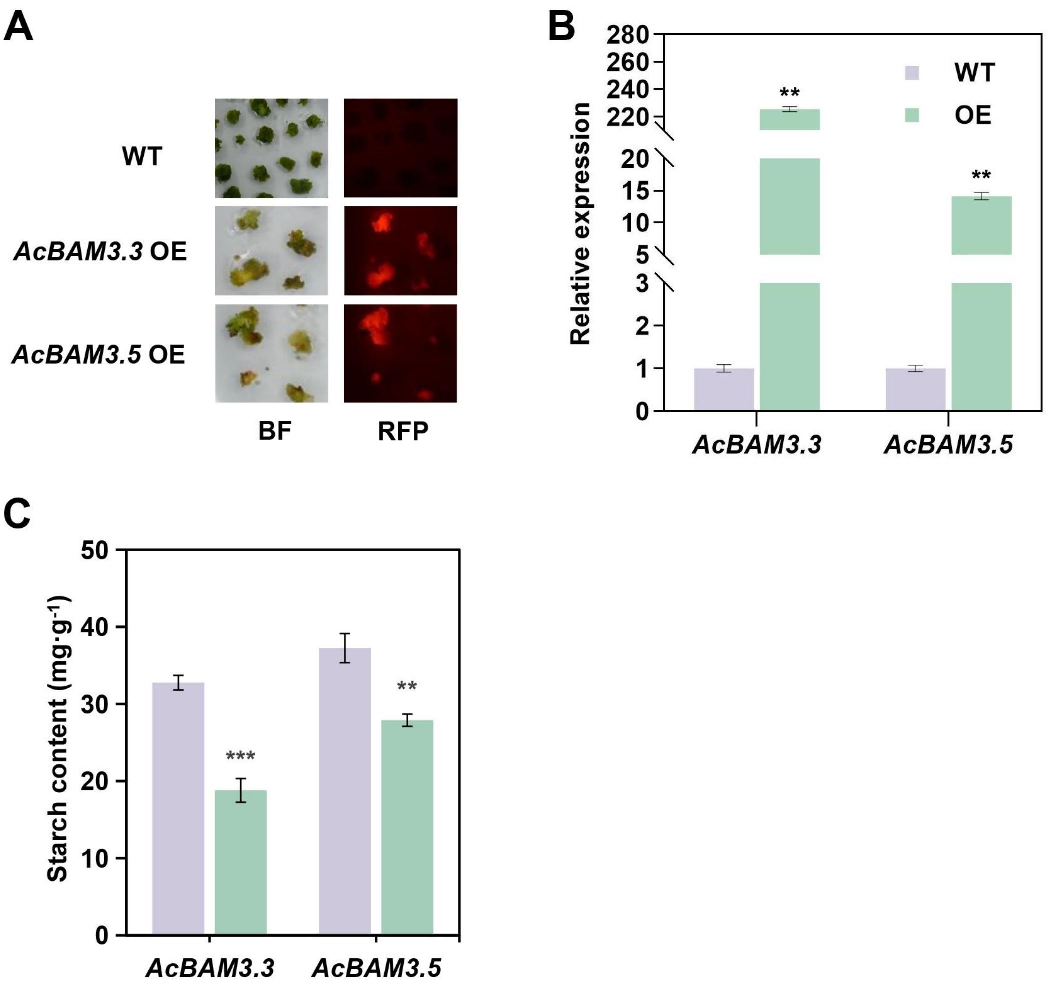

Supplementary Fig. 13 Analysis of starch degradation in kiwifruit callus overexpressing of *AcBAM3.3* and *AcBAM3.5*.

(A) Phenotypic appearance of wild-type (WT), *AcBAM3.3*-overexpressing (OE), and *AcBAM3.5*-OE callus in brightfield (BF) and red fluorescence light source (RFP) views.

(B) Expression analysis of *AcBAM3.3* and *AcBAM3.5* in WT, *AcBAM3.3*-OE, and *AcBAM3.5*-OE callus. Primers are shown in Supplementary Table 2.

(C) Starch content in WT, *AcBAM3.3*-OE, and *AcBAM3.5*-OE callus.

Data are mean ± SE of three replicates. Asterisks indicate significant differences as determined by Student's *t*-test (**, $P<0.01$; ***, $P<0.001$).

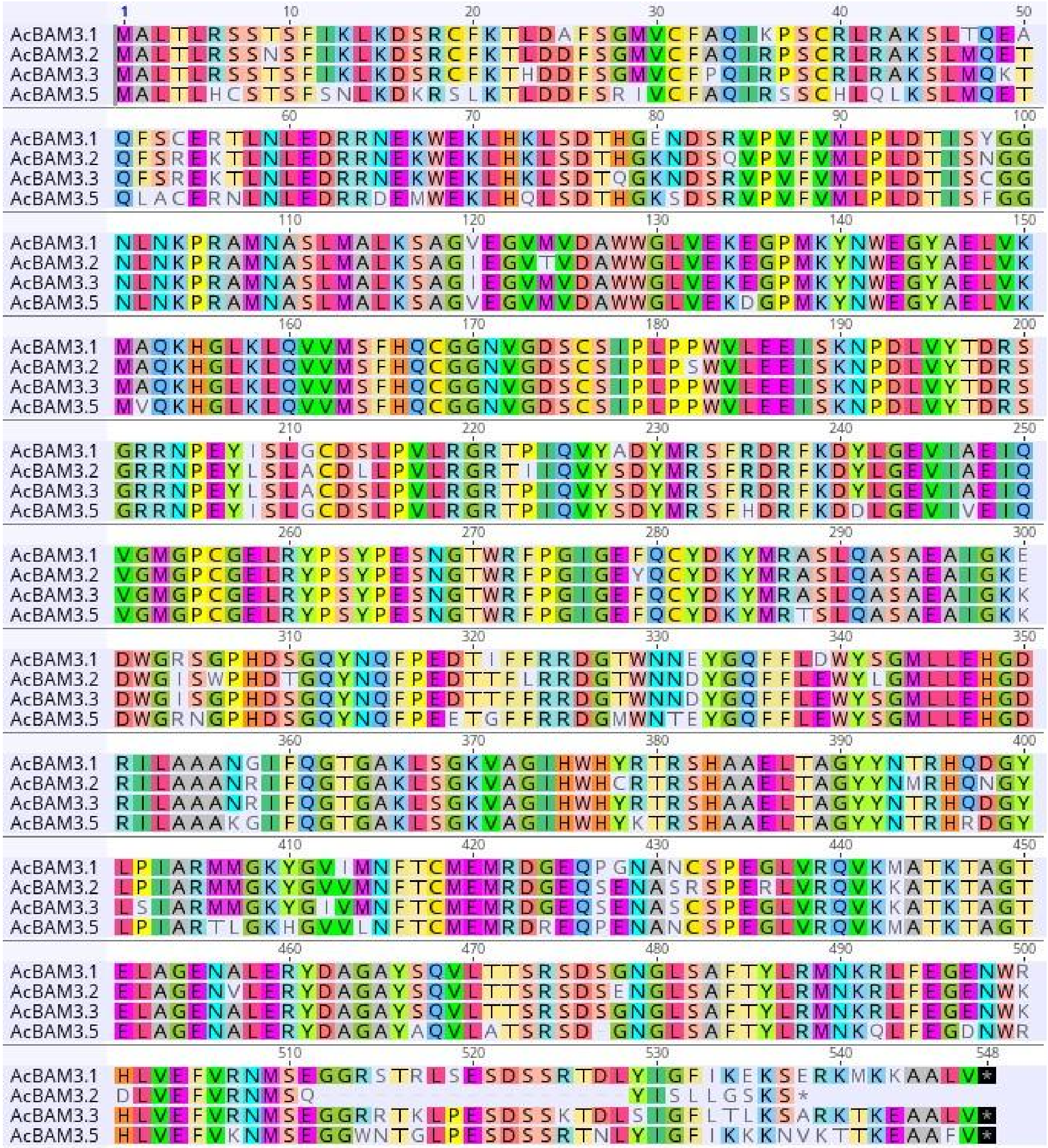

Supplementary Fig. 14 Amino acid alignment of AcBAM3s.
Amino acid sequences were aligned in Geneious (V2019.0.3).

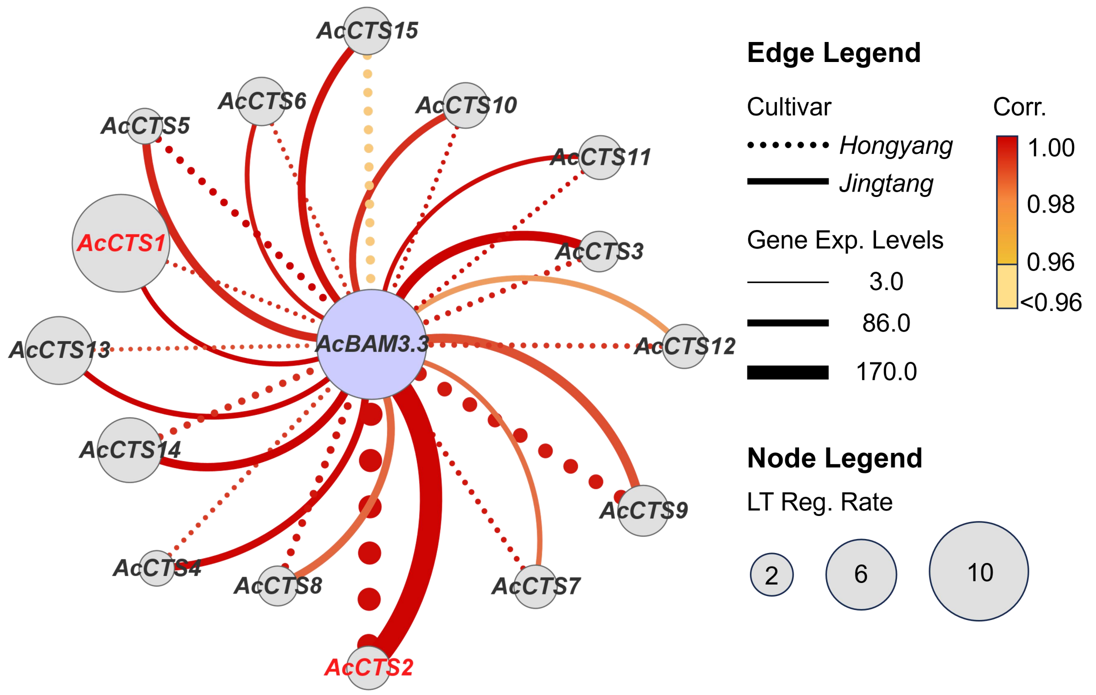

Supplementary Fig. 15 Correlation between *AcBAM3.3* expression and cool temperature-specific (CTS) transcription factors. Threshold: log2FC of CT/ room temperature (RT)>2, R>0.96. AcCTS1 (AcTINY2), Acc12510; AcCTS2 (AcSIG5.1), Acc05562; AcCTS3 (AcbZIP1), Acc23746; AcCTS4 (AcbZIP1), Acc12208; AcCTS5 (AcDBB1), Acc04028; AcCTS6 (AcDof1), Acc06345; AcCTS7 (AcDof2), Acc21930; AcCTS8 (AcDof3), Acc29872; AcCTS9 (AcERF1), Acc02810; AcCTS10 (AcERF2), Acc30992; AcCTS11 (AcMYB1), Acc29660; AcCTS12 (AcMYB2), Acc05338; AcCTS13 (AcNAC1), Acc33448; AcCTS14 (AcNAC2), Acc32998; AcCTS15 (AcNAC3), Acc08011. More detailed information was shown in Data Set S2 and S3.

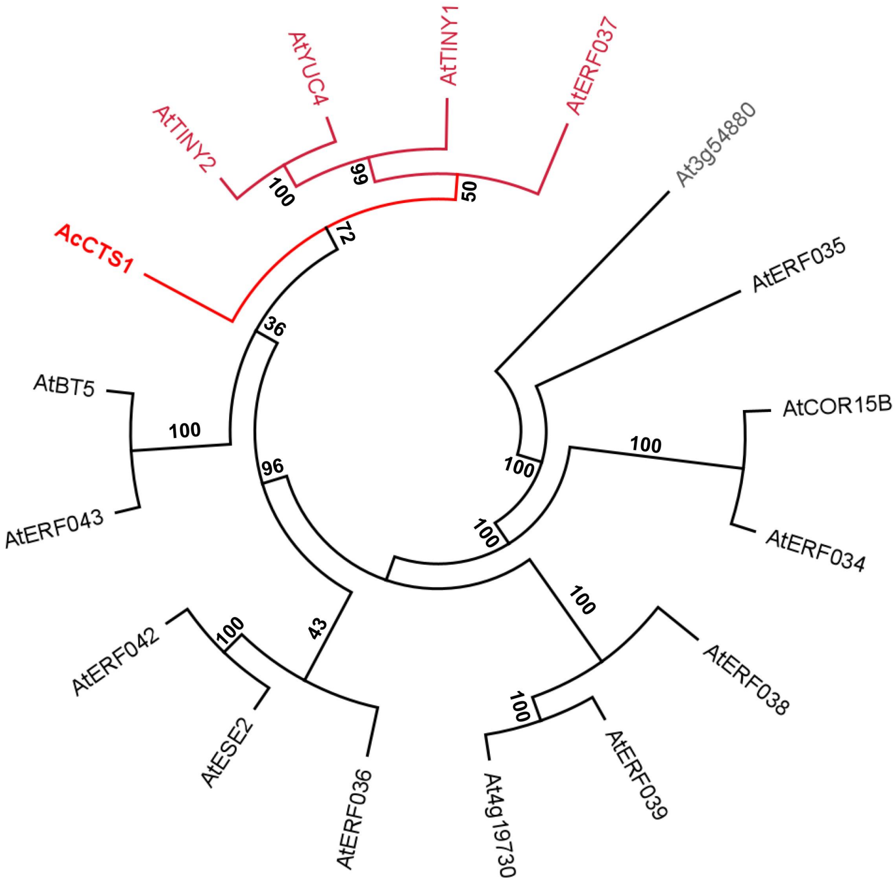

Supplementary Fig. 16 Phylogenetic analysis of amino acid sequences of AcCTS1 and ERFs from *Arabidopsis thaliana*. AcCTS1 and AtTINY2 showed the highest amino acid identity (48.7%). Sequence alignments were conducted using BLASTp in NCBI, with the non-redundant protein sequences (nr) database, limited to Arabidopsis. The remaining settings were left as defaults. Proteins with a BLAST score >130 were chosen for phylogenetic analysis. The phylogenetic tree was constructed using the neighbor-joining method of the MEGA7 software. Bootstrap analysis of 1,000 trials provided a reliable estimate of the topology of phylogenetic tree. The branch number showed the consensus support (%). AcCTS1, Acc12510; AtTINY1, AT5G25810; AtTINY2, AT5G11590; AtERF034, AT2G44940; AtERF035, AT3G60490; AtERF036, AT3G16280; AtERF037, AT1G77200; AtERF038, AT2G35700; AtERF039, AT4G16750; AtERF042, AT2G25820; AtERF043, AT4G32800; AtYUC4, AT5G11320; AtBT5, AT4G37610; AtCOR15B, AT2G42530; AtESE2, AT2G21670.

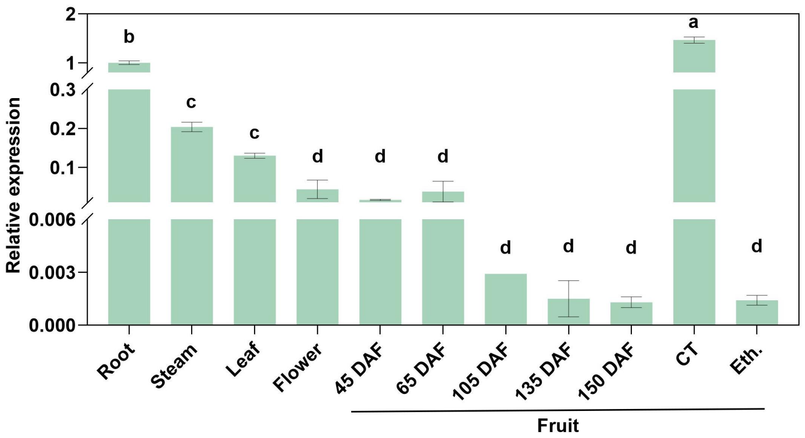

Supplementary Fig. 17 Expression profile of *AcCTS1* in kiwifruit. *AcCTS1* expression was analyzed across different tissues (Root; Stem; Leaf; Flower), fruit developmental stages (45, 65, 105, 135, 150 day after flowering, DAF), and postharvest treatments, including ethylene (Eth.) for 3 days, cool temperature (CT, 5 °C), and room temperature (RT, 20°C) at 15 day after storage (DAS). Data represent mean ± SE (n = 3). Different lowercase letters indicate significant differences ($P < 0.05$).

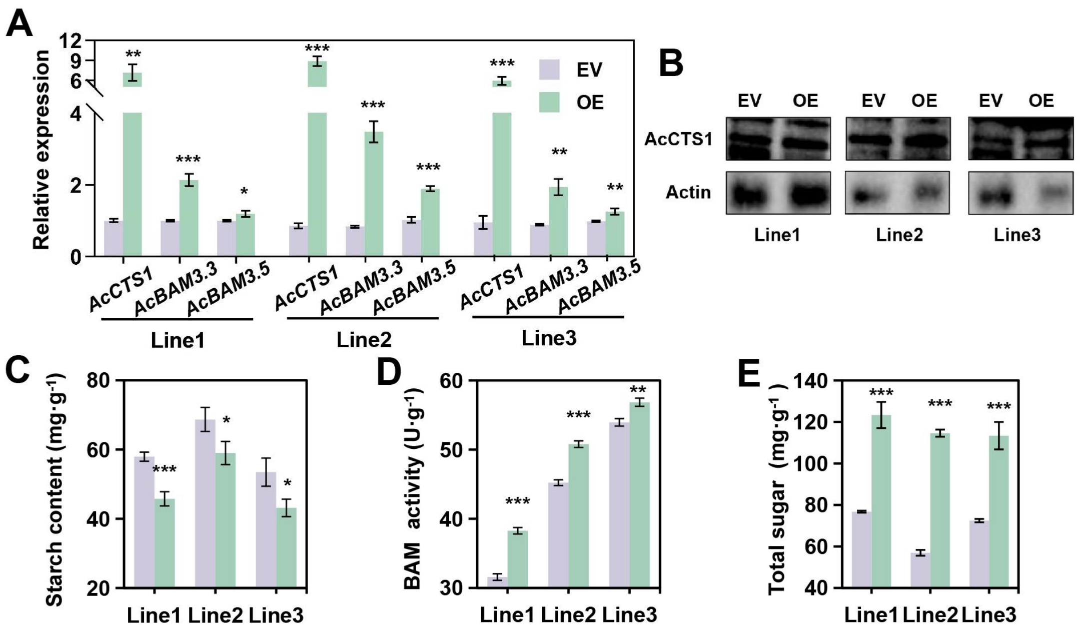

Supplementary Fig. 18 Transient overexpression of *AcCTS1* in kiwifruits enhances CT-induced starch degradation. A) Expression of *AcCTS1*, *AcBAM3.3*, and *AcBAM3.5* at the injection site of kiwifruit. B) Immunoblotting analysis of kiwifruit injected with empty vector and *AcCTS1*-OE. Actin was used as a loading control. C – E) Changes in starch content, BAM activity, and total sugar content in the injection site of kiwifruit. Data are means ± SE from three biological replicates. Asterisks indicate significant differences as determined by Student's *t*-test (*, $P<0.05$; **, $P<0.01$; ***, $P<0.001$).

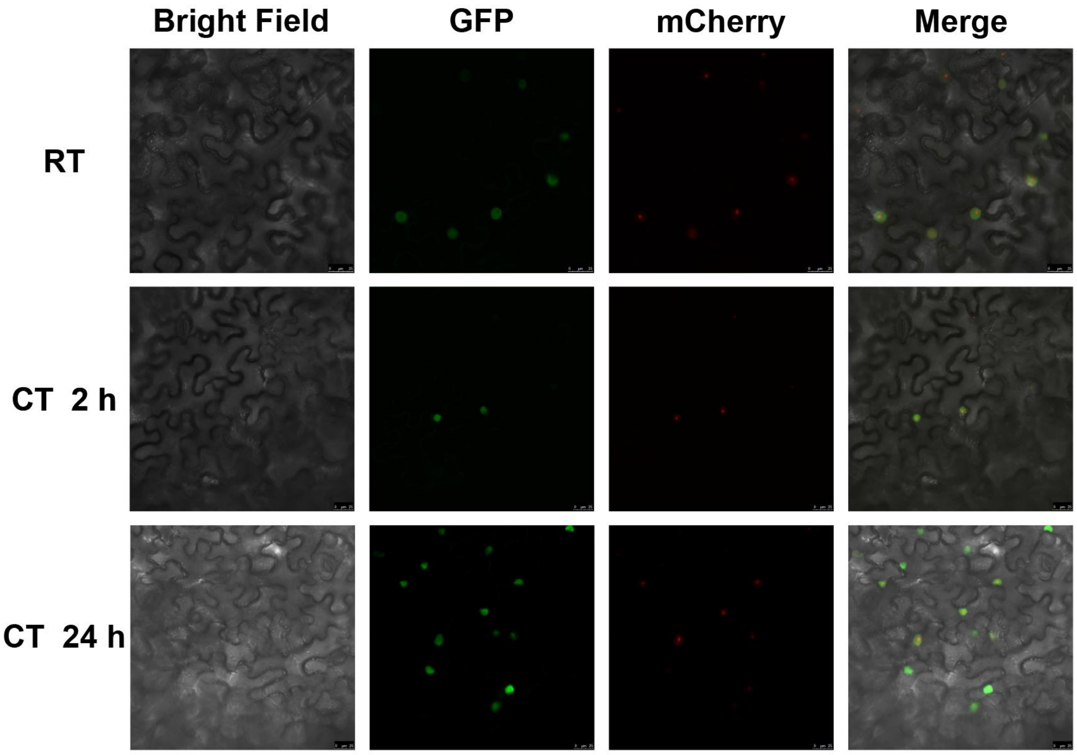

Supplementary Fig. 19 Subcellular localization of AcCTS1. Confocal microscopy images of *N. benthamiana* epidermal cells co-expressing AcCTS1-GFP (green) and the nuclear marker mCherry (red). From left to right: brightfield image, GFP channel, mCherry channel, and merged view. Scale bars are indicated in the bottom right of each panel.

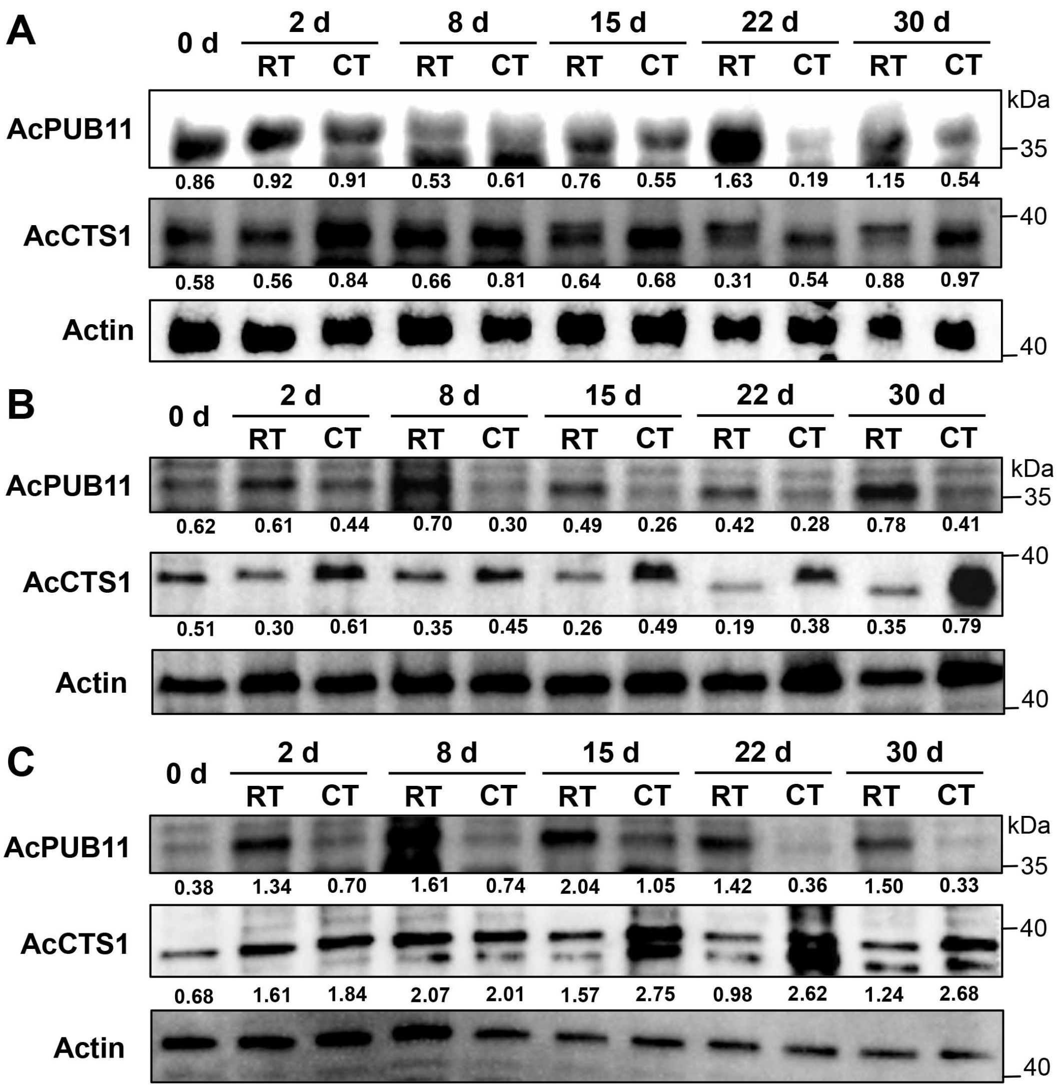

Supplementary Fig. 20 Changes in the abundance of AcPUB11 and AcCTS1 at RT (20°C) and CT (5°C) were assessed using western blot in 'Jintang No.3' (A), 'Hongyang' (B), and 'Jianxiang' (C). The anti-AcPUB11 and anti-AcCTS1 antibodies were used separately, with Actin serving as the loading control. Molecular weight markers (kDa) are shown on the right for each western blot.

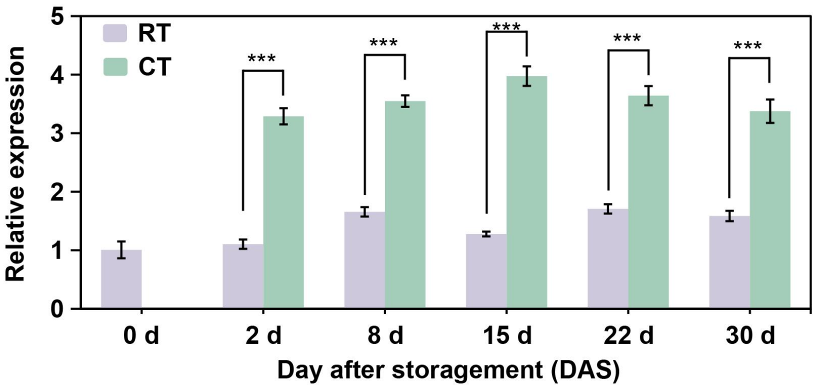

Supplementary Fig. 21 Expression analysis of *AcPUB11* at room temperature (RT) and cool temperature (CT). Relative expression levels of *AcPUB11* mRNA in kiwifruit outer pericarp during storage at RT (20°C) and CT (5°C), as determined by RT-qPCR. Data are mean ± SE (n = 3). Asterisks indicate a significant difference as determined by Student's *t*-test ($P < 0.001$).

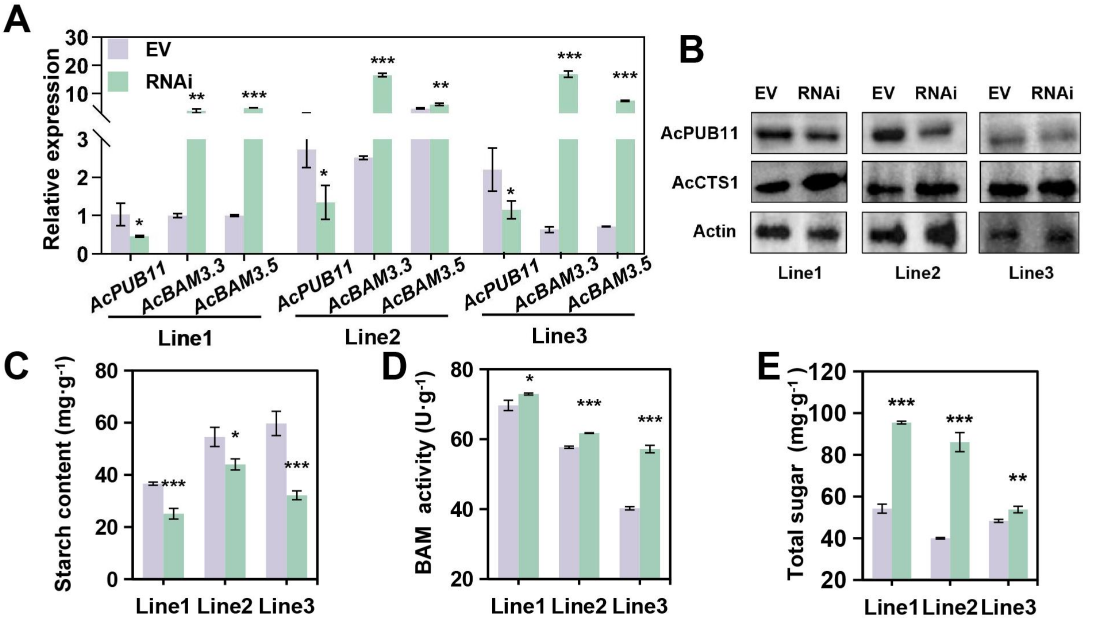

Supplementary Fig. 22 Transient silencing of *AcPUB11* in kiwifruit promotes CT-induced starch degradation. A) Expression of *AcPUB11*, *AcBAM3.3*, and *AcBAM3.5* at the injection site of kiwifruit. B) Immunoblotting analysis of kiwifruit injected with empty vector (EV) and *AcPUB11*-RNAi. Actin was used as a loading control. C–E) Changes in starch content, BAM activity, and total sugar content in the injection site of kiwifruit. Data are means ± SE from three biological replicates. Asterisks indicate significant differences as determined by Student's *t*-test (*, $P<0.05$; **, $P<0.01$; ***, $P<0.001$).

Supplementary Table 1. Genes identified by yeast two-hybrid screening using AcCTS1 as bait

Yeast Clone No.	Gene ID	BLAST identity (%)	Description
2	Acc32202.1	98.91	Kiwellin
3	Acc32202.1	98.29	Kiwellin
4	Acc10675.1	100	YABBY 2
5	0 hits found	\	\
6	Acc17723.1	99.81	Inactive exonuclease EXD1
7	Acc04863.1	95.9	Endochitinase A
8	Acc18864.1	100	Metallothionein-like protein type 3
11	0 hits found	\	\
13	Acc00338.1	97.95	Endochitinase EP3
20	Acc03574.1	99.67	60S ribosomal protein L15
21	Acc21654.1	98.56	Polyubiquitin
22	Acc33269.1	100	40S ribosomal protein S8
25	Acc33269.1	100	40S ribosomal protein S8
26	Acc33269.1	100	40S ribosomal protein S8
27	Acc09233.1	98.91	Plant U-box protein 11
32	Acc00338.1	94.74	Endochitinase EP3
33	Acc20571.1	97.75	Polyubiquitin
34	Acc18864.1	100	Metallothionein-like protein type 3
35	Acc32202.1	99.07	Kiwellin
36	Acc06504.1	96.34	Thebaine 6-O-demethylase
37	Acc33269.1	100	40S ribosomal protein S8
39	Acc21654.1	98.56	Polyubiquitin
44	Acc11113.1	98.8	GTP-binding protein SAR1A
46	Acc27868.1	98.98	Expansin-A8
47	Acc23900.1	99.83	Deubiquitinating enzyme 36
50	Acc23900.1	99.83	Deubiquitinating enzyme 36
56	Acc14525.1	97.07	RING-type E3 ubiquitin transferase
58	Acc32202.1	99.07	Kiwellin
59	Acc11295.1	99.45	Zinc finger protein ZAT10

* Only successfully sequenced yeast clones are listed.

Supplementary Table 2. Primers used in this study

Vector or Gene	Sequence
pKlic1.0-AcCTS1-F	actagttccagggcgcccgggATGAGCACTGAAAGCTGCTCAA
pKlic1.0-AcCTS1-R	atcatcgacccgacgcccgggTCAATAATCCCACAACACAGCC
pKlic1.0-AcCTS2-F	actagttccagggcgcccgggATGGGAGTTGTAACTGTTTCTAGC
pKlic1.0-AcCTS2-R	atcatcgacccgacgcccgggTTAGAAAACGTAACGGCGAAGATAA
pGreen0800II-LUC-AcBAM3.3 promoter-F	ggtggagatcgaattccatgg GGAATGAGCATCCAAATTCATG
pGreen0800II-LUC-AcBAM3.3 promoter-R	tgtttttggcgtcttccatggATGATGTTTCTTCTTGGTTTTGTGA
pGreen0800II-LUC-AcBAM3.5 promoter-F	ggtggagatcgaattccatggTGGCTGAGTTTGTGGGGTTT
pGreen0800II-LUC-AcBAM3.5 promoter-R	tgtttttggcgtcttccatgg ATTCAATTCTTCTGGGTTTTCTGAA
pAbAi-AcBAM3.3 promoter-F	cttgaattcgagctcggtaccGGAATGAGCATCCAAATTCATG
pAbAi-AcBAM3.3 promoter-R	atacagagcacatgcctcgagATGATGTTTCTTCTTGGTTTTGTGA
pGADT7-AcCTS1-F	gccatggaggccagtgaattcATGAGCACTGAAAGCTGCTCAA
pGADT7-AcCTS1-R	cagctcgagctcgatggatccTCAATAATCCCACAACACAGCC
pGADT7-AcPUB11-F	gccatggaggccagtgaattcTCATGGAAGTGAAGCATCGGACG
pGADT7-AcPUB11-R	cagctcgagctcgatggatccTTATGAAGAGCGACTTGATAAAGAA
pK7GW35s-AcBAM3.1-F	gtacaaaaaagcaggcttcATGGCTTTGACGCTACGTT
pK7GW35s-AcBAM3.1-R	tgtacaagaaagctgggtccccCACTAGAGCGGCCTTCTTC
pK7GW35s-AcBAM3.2-F	gtacaaaaaagcaggcttcATGGCTTTGACGCTACGTT
pK7GW35s-AcBAM3.2-R	tgtacaagaaagctgggtccccTGATTTTGATCCCAGTAAACTAATA
pK7GW35s-AcBAM3.3-F	gtacaaaaaagcaggcttcATGGCTTTGACGCTACGCTC
pK7GW35s-AcBAM3.3-R	tgtacaagaaagctgggtccccCACTAGAGCAGCCTCCTTC
pK7GW35s-AcBAM3.5-F	gtacaaaaaagcaggcttcATGGCTTTAACATTACATTGTTCGA
pK7GW35s-AcBAM3.5-R	tgtacaagaaagctgggtccccCACAAAAGCAGCCTCCTTG
pK7GW35s-AcCTS1-F	gtacaaaaaagcaggcttcATGAGCACTGAAAGCTGCTCAA
pK7GW35s-AcCTS1-R	tgtacaagaaagctgggtccccATAATCCCACAACACAGCCCC
ph7lic -AcPUB11-F （GFP-AcPUB11）	attacgccgaggTCATGGAAGTGAAGCATCGGACG
ph7lic -AcPUB11-R （GFP-AcPUB11）	tagggaagaggTTATGAAGAGCGACTTGATAAAGAA
pHELLSGATE 8-AcCTS1-F	gtacaaaaaagcaggcttcATGAGCACTGAAAGCTGCTCAA
pHELLSGATE 8-AcCTS1-R	tgtacaagaaagctgggtccccTTCGGGGAAGTTGAGCACAG
pHELLSGATE 8-AcPUB11-F	gtacaaaaaagcaggcttcATGGAAGTGAAGCATCGGACG
pHELLSGATE 8-AcPUB11-R	tgtacaagaaagctgggtccccGGGGTTGAGGAGGGCGTG
pGBKT7-AcCTS1-F	atggccatggaggccgaattcATGAGCACTGAAAGCTGCTCAA
pGBKT7-AcCTS1-R	ccgctgcaggtcgacggatccTCAATAATCCCACAACACAGCC
pGEX-4T-1-AcPUB11-F	atcggatctggttccgcgtggatccATGGAAGTGAAGCATCGGACG
pGEX-4T-1-AcPUB11-R	gctcgagtcgacccgggaattcTTATGAAGAGCGACTTGATAAAGAA
pMAL-C6T-AcCTS1-F	tccagatgctgatgggcggccgcATGAGCACTGAAAGCTGCTCAA
pMAL-C6T-AcCTS1-R	acctgcagggaattcggatccTCAATAATCCCACAACACAGCC
ph7lic-AcCTS1-F (HA-AcCTS1)	attacgccgaggTCATGAGCACTGAAAGCTGCTCAA
ph7lic-AcCTS1-R (HA-AcCTS1)	tagggaagaggTCAATAATCCCACAACACAGCC
ph7lic-GUS-F（GFP-GUS）	attacgccgaggTCATGTTACGTCCTGTAGAAACCC
ph7lic-GUS-R（GFP-GUS）	tagggaagaggTCATTGTTTGCCTCCCTGCT
pMDC43-nYFP-AcCTS1-F	gtacaaaaaagcaggcttcATGAGCACTGAAAGCTGCTCAA
pMDC43-nYFP-AcCTS1-R	tgtacaagaaagctgggtccccTCAATAATCCCACAACACAGCC
pMDC43-cYFP-AcPUB11-F	gtacaaaaaagcaggcttcATGGAAGTGAAGCATCGGACG
pMDC43-cYFP-AcPUB11-R	tgtacaagaaagctgggtccccTTATGAAGAGCGACTTGATAAAGAA
pCDFD-AcCTS1-F	gagggaaggatttcagaattcATGAGCACTGAAAGCTGCTCAA
pCDFD-AcCTS1-R	aacgtcgtatgggtaaggcctATAATCCCACAACACAGCCCC
pACYCD-AcPUB11-F	gcatcaccatcatcaccaagccagggatccATGGAAGTGAAGCATCGGA
pACYCD-AcPUB11-R	cctcggagatgagcttctgctcaggcctTGAAGAGCGACTTGATAAAGAA TCA
BAM3.3 promoter-F (FAM labeled, EMSA)	AAAACTACACTTGCCGACTACAAATCCTCT
BAM3.3 promoter-F (Cold probe,EMSA)	AAAACTACACTTGCCGACTACAAATCCTCT
BAM3.3 promoter-R (EMSA)	AGAGGATTTGTAGTCGGCAAGTGTAGTTTT
BAM3.3 promoter-F (Mutant probe, EMSA)	AAAACTACACTTAAAAAATACAAATCCTCT
BAM3.3 promoter-R (Mutant probe, EMSA)	AGAGGATTTGTATTTTTTAAGTGTAGTTTT
BAM3.5 promoter-F (FAM labeled, EMSA)	TTCAGAAATAGCTCCGACGGCCGTTCTCAC
BAM3.5 promoter-F (Cold probe,EMSA)	TTCAGAAATAGCTCCGACGGCCGTTCTCAC
BAM3.5 promoter-R (EMSA)	GTGAGAACGGCCGTCGGAGCTATTTCTGAA

Vector or Gene	Sequence
BAM3.5 promoter-F (Mutant probe, EMSA)	TTCAGAAATAGCTAAAAAGGCCGTTCTCAC
BAM3.5 promoter-R (Mutant probe, EMSA)	GTGAGAACGGCCTTTTTAGCTATTTCTGAA
AcCTS1-F (Hi-TOM)	CTGAAAGCTGCTCAAACTCG
AcCTS1-R (Hi-TOM)	GATCCGTTTGATTTTCGTCAGG
AcPUB11-F (Hi-TOM)	AGACAGCGAATTGGAGATGG
AcPUB11-R (Hi-TOM)	ATATGAGACGAAGCTCGCAG
Actin-F (RT-qPC)	TGCATGAGCGATCAAGTTTCAAG
Actin-R (RT-qPC)	TGTCCCATGTCTGGTTGATGACT
AcCTS1-F (RT-qPC)	CGAGTTACGAATCCAACGAGTC
AcCTS1-R (RT-qPC)	CAGCCCCAAAACTGCTTTG
AcPUB11-F (RT-qPC)	GTTCCTGCTTTGTTCTCGTTG
AcPUB11-R (RT-qPC)	ATAACCCCAATACCCGAAACC
AcBAM3.3-F (RT-qPC)	CCGAATACCTATCCTTGGCTTG
AcBAM3.3-R (RT-qPC)	GAATTTCCGCAATAACCTCGC
AcBAM3.5-F (RT-qPC)	GGAACTGGAGCTAAACTATCTGG
AcBAM3.5-R (RT-qPC)	TCCATGTTTACCCAGTGTACG
