# Supplementary material for: A cool temperature–induced ubiquitination-controlled transcription factor promotes starch degradation and ripening in kiwifruit
Source: Plant Commun. 2026 Jan 21;7(7):101736. doi: 10.1016/j.xplc.2026.101736 (PMC13370233; doi:10.1016/j.xplc.2026.101736)
Supplement: Document S2. Article plus supplemental information [file mmc5.pdf]

Plant Communications
Research article

CellPress
Partner Journal

A cool temperature–induced ubiquitination-controlled transcription factor promotes starch degradation and ripening in kiwifruit

Ang Li[1], Yunhe Meng[1], Xiaoya Chen[1], Zhebin Zeng[1], Zhidan Zhao[1], Tiantian Li[1], Gang Ding[1], Ross G. Atkinson[2], Yue Huang[3], Yunjiang Cheng[1], Xiuxin Deng[1] and Yunliu Zeng[1,*]

[1]National Key Laboratory for Germplasm Innovation & Utilization of Horticultural Crops, Joint International Research Laboratory of Germplasm Innovation & Utilization of Horticultural Crops, National R&D Centre for Citrus Preservation, College of Horticulture and Forestry Science, Huazhong Agricultural University, Wuhan 430070, China

[2]The New Zealand Institute for Bioeconomy Science Ltd. (formerly Plant and Food Research Ltd.), Private Bag, Auckland 92169, New Zealand

[3]School of Horticulture, Anhui Agricultural University, Hefei 230036, China

***Correspondence: Yunliu Zeng (zengyl@mail.hzau.edu.cn)**

https://doi.org/10.1016/j.xplc.2026.101736

ABSTRACT

Ripening of kiwifruit (*Actinidia* spp.) is highly sensitive to ethylene, but reliance on exogenous ethylene often results in over-softening, greatly reducing shelf life. Here, we discovered a pathway induced by cool temperature (CT; 5°C–10°C) that directly orchestrates starch-to-sugar conversion in kiwifruit under conditions in which ethylene perception is inhibited by 1-methylcyclopropene. Through transcriptomic and metabolomic profiling, we identified *AcBAM3.3*, a β-amylase gene that is specifically induced by CT but not by ambient temperature. A CT-inducible ERF transcription factor, *AcCTS1* (CT-specific factor 1), was found to directly bind the promoters of *AcBAM3.3* and *AcBAM3.5* and activate their transcription, as confirmed by dual-luciferase, electrophoretic mobility shift, and yeast one-hybrid assays. We also identified an E3 ubiquitin ligase, AcPUB11, which targets AcCTS1 for 26S proteasomal degradation, repressing starch degradation at room temperature. Under CT, reduced AcPUB11 abundance allows for AcCTS1 accumulation, driving *AcBAM3.3* and *AcBAM3.5* expression and promoting ripening. Functional characterization via overexpression, RNAi, and CRISPR–Cas9 in both callus and fruit confirmed the AcPUB11–AcCTS1–AcBAM3s module as the central regulator of CT-induced starch metabolism. Our findings define a ubiquitination-controlled transcriptional regulatory module that mediates fruit adaptation to cool environments, providing a mechanistic foundation for temperature-controlled starch degradation during ripening.

Key words: starch metabolism, cool temperature, ubiquitination, transcription factor, fruit ripening, *Actinidia* spp

Li A., Meng Y., Chen X., Zeng Z., Zhao Z., Li T., Ding G., Atkinson R.G., Huang Y., Cheng Y., Deng X., and Zeng Y. (2026). A cool temperature–induced ubiquitination-controlled transcription factor promotes starch degradation and ripening in kiwifruit. Plant Comm. **7**, 101736.

INTRODUCTION

Starch is a key determinant of fruit flavor; its degradation is closely linked to sugar accumulation and fruit softening, making it an important quality attribute for fruit growers and consumers. Starch metabolism is primarily regulated by ethylene, together with environmental factors such as temperature, light, and nutrients (Dale and Bradshaw, 2003; Centeno et al., 2011; Lim et al., 2022). Ethylene plays a crucial role in inducing the ripening of climacteric horticultural fruits, significantly affecting the postharvest ripening of fruits such as kiwifruit, bananas, and mangoes. However, because these fruits are highly sensitive to ethylene, it can lead to rapid over-ripening, significantly reducing shelf life and causing substantial postharvest losses (Zhang et al., 2018; Wei et al., 2023a). Kiwifruit (*Actinidia* spp.) is an atypical climacteric fruit that accumulates high levels of starch (up to 40% of its dry matter at harvest; Li and Zhu, 2018) and requires further starch degradation during ripening for edibility. Interestingly, starch degradation occurs in the absence of detectable ethylene during the early stages of ripening. Recent studies have suggested that specific temperatures can bypass classic ethylene signaling to promote fruit ripening (Asiche et al., 2018), but the molecular mechanisms that underlie ethylene-independent ripening, including starch degradation, remain unclear.

© 2026 The Author(s). Published by Elsevier Inc. on behalf of CAS Center for Excellence in Molecular Plant Sciences, Chinese Academy of Sciences, and Chinese Society for Plant Biology.
This is an open access article under the CC BY license (http://creativecommons.org/licenses/by/4.0/).

Temperature significantly influences fruit ripening. Cold temperatures (0°C–2°C) delay ripening during long-term storage by inhibiting softening, limiting starch-degrading enzyme activity, and reducing respiratory rate (Sanchez-Ballesta et al., 2020). Fruits, especially ethylene-sensitive horticultural crops, typically ripen as temperature increases. For instance, storage of tomatoes at 5°C significantly delays ripening and starch degradation, whereas storage at 25°C accelerates the production of flavor compounds and ethylene (Pék et al., 2010). Similar phenomena have been observed in apples and pears (Li et al., 2021, 2023). However, the simple dichotomy of freezing vs. high temperatures is insufficient to describe fruit temperature responses, as fruits respond differently across specific temperature ranges. Cool temperature (CT)-induced ripening refers to the phenomenon in which certain fruits actually ripen faster when exposed to CTs, in contrast to the typical expectation that lower temperatures slow ripening. For example, 4°C has been shown to induce changes in the sweetness of potatoes (Zhang et al., 2014), lowering tuber quality. Conversely, degreening of citrus fruits is promoted at 15°C (Mitalo et al., 2020), increasing fruit quality. Two major metabolic changes induced by CT ripening have been identified: starch degradation and sugar accumulation. Comparative transcriptomic analyses of kiwifruit stored at 5°C or 20°C/22°C revealed significant upregulation of several genes involved in starch degradation at 5°C without detectable ethylene, and this upregulation was correlated with decreasing firmness and increasing soluble sugar content (Mworia et al., 2012; Asiche et al., 2018; Mitalo et al., 2019). However, the molecular regulation of genes involved in starch degradation and ripening under CT remains largely unknown.

Unlike the transcriptional regulators involved in ethylene signaling, those that govern temperature-induced ripening are poorly characterized. Responses to freezing and chilling stress are known to involve C-repeat binding factor (CBF) and dehydration-responsive element-binding 1 (DREB1) (Shi et al., 2018; Zhao et al., 2023), but less is known about the regulatory factors that mediate ripening at cool, non-stressful temperatures. Ethylene response factors (ERFs) are key regulators of ripening in climacteric fruits, typically acting downstream of ethylene; for example, several ERFs integrate ethylene signals to control ripening in tomato and apple (Li et al., 2016; Deng et al., 2022). However, it is unknown whether ERFs or other transcription factors (TFs) can be activated by CT to directly orchestrate ripening processes independently of ethylene. Identification of such ethylene-independent, temperature-responsive transcriptional networks is essential for understanding the full spectrum of ripening regulation.

A range of TFs that modulate starch degradation in fruits in response to ethylene have also been reported. For instance, the ethylene-induced apple TF MdWRKY32 is involved in starch–sugar metabolism, binding to the *MdBAM5* (β-amylase [BAM]) promoter and activating its expression during storage (Li et al., 2021). The transcriptional regulatory network for starch degradation in banana is well characterized. MabHLH6 and MabZIP21 act as positive regulators of starch degradation by directly targeting the promoters of multiple genes involved in starch degradation (Xiao et al., 2018; Xu et al., 2024). In addition, MaMYB3, which targets the promoters of *MabHLH6* and *bHLH10*, has been identified as a negative regulator of starch degradation (Fan et al., 2018). Compared with those in banana, the transcriptional mechanisms that control starch degradation in kiwifruit are less well understood. Kiwifruit AdDof3 promotes starch degradation by activating the key starch degradation gene *AdBAM3L* in response to ethylene (Zhang et al., 2018), but whether signals other than ethylene are involved remains unclear. Proteasomal degradation of the MYB TF MaMYB60 under high-temperature stress, mediated by the E3 ligase MaBAH1, was recently shown to weaken its direct activation of chlorophyll catabolism genes, inhibiting chlorophyll degradation (Wei et al., 2023a). These studies indicate that specific temperatures may be another signal for fruit ripening, acting through posttranslational modifications such as histone modification and ubiquitination.

In this study, using a system in which ethylene is suppressed, we investigated the molecular mechanisms underlying CT-induced starch degradation during ripening in kiwifruit, a phenomenon that contrasts with typical behavior observed at other temperatures, such as room temperature (RT) and cold temperature, even when ethylene signaling is blocked. Our results show that CTs between 5°C and 10°C serve as a signal that triggers ripening and starch degradation in kiwifruit. Through multi-omics analyses and biochemical and molecular studies, we identified a CT-induced module involving AcPUB11, AcCTS1, and AcBAM3s that regulates starch degradation during ripening. These findings reveal a core temperature-sensing mechanism mediated by a ubiquitination-controlled TF, providing precise tools for the management of postharvest quality driven by CT.

RESULTS

Cool temperature induces ripening in kiwifruit

To investigate the effect of non-stressful temperatures on ripening, three kiwifruit cultivars were exposed to a range of temperatures: cold (1°C), CT (5°C, 10°C, and 15°C), and RT (20°C). The CTs represented the range of autumnal temperatures in major kiwifruit growing regions of the world. Healthy fruit without physical damage were selected, individually bagged, and treated with 1-methylcyclopropene (1-MCP) to prevent ethylene action. Ethylene production was measured prior to each physiological assessment, and only ethylene-free fruit were included in the CT experiments (Supplemental Figure 1). Exposure of kiwifruit to ethylene triggers rapid ripening, with soluble solid content (SSC) rising from 5.7°Bx to 12.2°Bx and firmness dropping by 46.3 N (52.1–5.8 N) within 2 days (Supplemental Figure 2). Any fruit that showed such accelerated ripening were removed from the CT experiments.

Yellow-fleshed ‘Jintang No. 3’ (JT) fruit exhibited greater softening after 30 days of CT treatment than fruit held at RT or cold temperature for 30 days (Figure 1A). Starch content was also significantly reduced (26%–35%) at CT compared with RT and cold temperature (Figure 1A). SSC, which represents the production of sugars from starch degradation, was ~25% higher at 5°C and 10°C than at 1°C and RT (Figure 1A). Similar trends were observed in red-fleshed ‘Hongyang’ (HY; Supplemental Figure 3) and green-fleshed ‘Cuixiang’ (Supplemental Figure 4) kiwifruit.

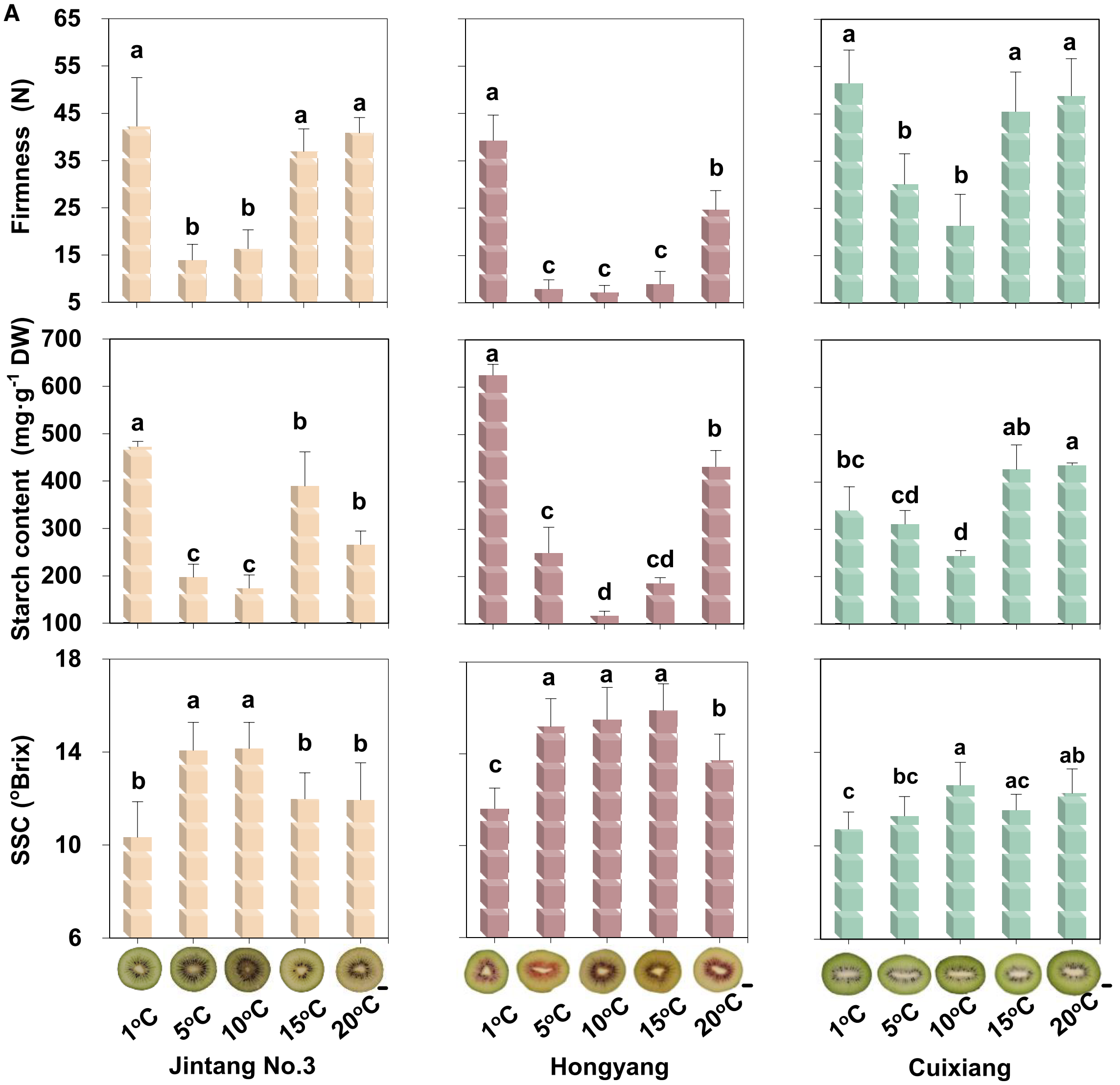

B

Cultivars	Rate of change in	Storage temperature 1°C	5°C	10°C	15°C	20°C	r (Starch/Firmness)
JT	Starch	9.0	18.2	18.9	11.8	15.9	0.81
	Firmness	0.3	1.3	1.2	0.5	0.4	
HY	Starch	4.5	21.6	27.6	24.5	13.3	0.98
	Firmness	0.5	1.9	2.0	1.9	1.2	
CX	Starch	14.0	15.9	20.4	8.2	7.6	0.81
	Firmness	0.5	1.9	2.5	0.9	0.7	

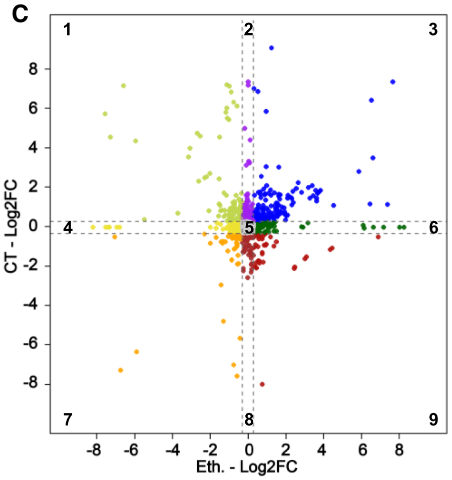

Figure 1. Physiological and metabolite changes during storage at different temperatures in 1-MCP-treated kiwifruit.
(A) Fruit firmness, starch content, and soluble solid content (SSC; degrees Brix) in JT after 30 days of storage, HY after 22 days of storage, and 'Cuixiang' after 15 days of storage at 1°C, 5°C, 10°C, 15°C, and 20°C. Detailed physiological data are provided in Supplemental Figures 3–5. Data are means ± SE ($n = 3$). Significant differences ($P < 0.05$) are indicated by lowercase letters.

(legend continued on next page)

The structure of starch granules appeared looser in fruit stored at 10°C than in those stored at 1°C and RT, as observed under a scanning electron microscope (Supplemental Figure 6). Moreover, fruit stored at 5°C and 10°C exhibited a faster rate of decline in firmness and starch content than fruit stored at RT or 1°C, with the correlation between starch content and firmness greater than 0.8 in all 3 cultivars (Figure 1B). Taken together, these findings indicate that CTs of 5°C–10°C promote ripening and starch degradation in the absence of detectable ethylene production in all 3 kiwifruit cultivars compared with RT (20°C) and cold temperature (1°C).

To systematically compare the metabolic basis of ripening induced by CT versus ethylene, we performed widely targeted metabolomic profiling using high performance liquid chromatography (HPLC)–tandem mass spectrometry (MS/MS) (Supplemental Dataset 1) of HY fruit ripened under CT at 10°C (CTRK) and under ethylene treatment (ERK) (Supplemental Figure 7). Hierarchical clustering and principal component analysis showed that the metabolomes were highly correlated among the three biological replicates of each treatment and that the metabolites of CTRK and ERK were divided into two separate clusters (Supplemental Figures 8A and 8B). As shown in Figure 1C, metabolites associated with starch degradation during fruit ripening, such as D-sucrose and D-glucose, were identified in both CTRK and ERK (quadrant 3). One hundred metabolites were identified only in ERK (quadrant 6) and were mainly involved in phenolic acid metabolism (22.0%; Supplemental Figure 8C), and 99 metabolites were identified only in CTRK (quadrant 2) and were mainly involved in lipid metabolism (26.3%; Supplemental Figure 8C). The distinct metabolic profile of CT-mediated ripening thus supports the existence of an ethylene-independent pathway that regulates postharvest ripening and starch degradation in kiwifruit.

The β-amylase *AcBAM3.3* is involved in cool temperature-induced starch degradation

To characterize the molecular mechanisms that drive CT-induced starch degradation during kiwifruit ripening, we performed transcriptomic analysis of CTRK and ERK samples from JT (Supplemental Dataset 2) and HY (Supplemental Dataset 3). Nine-quadrant association analysis of differentially expressed genes (DEGs) was used to identify key genes expressed specifically in response to CT. As shown in Figure 2A and 2B, 523 genes in JT and 437 genes in HY were induced by CT rather than ethylene (quadrants 1 and 2), and 133 genes were specifically upregulated by CT in both cultivars (Figure 2C). These genes were highly enriched in the Gene Ontology terms "fruit ripening" and "photosynthesis" (Supplemental Figure 9). Of particular interest was the specific upregulation of *AcBAM3.3*, which encodes a BAM potentially involved in starch degradation. Three related BAM genes—*AcBAM3.1*, *AcBAM3.2*, and *AcBAM3.5*—were induced under both ethylene and CT treatments (Figure 2D). Multiple sequence alignments revealed that the four *AcBAM3* genes shared a conserved glycosyl hydrolase domain (Supplemental Figure 10) and were likely to be active BAMs.

To determine the functional role of these four AcBAM3s in starch degradation, we measured BAM activity in outer pericarp tissue of JT ripened at CT or RT. As shown in Figure 2E, BAM activity continuously increased under CT, with a 1.4-fold increase after 22 days, paralleling the pattern of starch degradation during fruit ripening. We next assessed the potential of the four *AcBAM3s to mediate starch degradation upon* transient overexpression (OE) in *Nicotiana benthamiana* (*N. benthamiana)* leaves. Iodine/potassium iodide staining revealed that OE of *AcBAM3.1*, *AcBAM3.3*, or *AcBAM3.5* resulted in increased starch degradation (16%–60%) compared with the empty vector (EV) control (Supplemental Figure 11). Further evidence for the function of *AcBAM3.3* and *AcBAM3.5* in starch degradation was obtained by their OE in 'Micro-Tom' tomato fruit (Figure 2F, Supplemental Figure 12) and kiwifruit calli (Supplemental Figure 13). *AcBAM3.1* was not overexpressed, as its encoded protein showed >92% amino acid identity to AcBAM3.3 (Supplemental Figure 14). These results showed that OE of *AcBAM3.3* and *AcBAM3.5* reduced starch content to 25%–52% of that in the wild-type (WT) control.

Identification of *AcCTS1*, a transcriptional activator of *AcBAM3.3/3.5*

To identify upstream regulators of *AcBAM3.3* expression, we analyzed the 133 genes that were specifically upregulated by CT in both JT and HY. Fifteen TFs showed a strong correlation with *AcBAM3.3* transcript levels (log2FC of CT/RT > 2, R > 0.96; Supplemental Figure 15). Based on their specific expression patterns, they were named CT-specific (CTS) TFs. *AcCTS1* showed the greatest fold increase between CT and RT in both cultivars (9.4-fold in JT and 5.6-fold in HY), and *AcCTS2* showed the highest expression at CT in both cultivars. The transcriptional effect of *AcCTS1* and *AcCTS2* on the 1918-bp *AcBAM3.3* promoter was tested in transient dual-luciferase transactivation assays. The firefly luciferase (LUC) reporter gene was placed under the control of the *AcBAM3.3* promoter and co-infiltrated with constructs overexpressing either *AcCTS1* or *AcCTS2* as effectors. The activity of the *AcBAM3.3* promoter was significantly enhanced in the presence of *AcCTS1*, with LUC/*Renilla* (REN) ratios increasing by approximately 3.0-fold compared with the control (Figure 3A). No activation was observed with *AcCTS2*.

To test whether AcCTS1 directly interacts with the *AcBAM3.3* promoter, we performed a yeast one-hybrid (Y1H) assay using the *AcBAM3.3* promoter as bait and the full-length coding sequence of *AcCTS1* as prey. As shown in Figure 3B, no basal activity was detected in yeast cells co-transformed with the

(B) Rate of decline in starch content (mg g^{-1}/day) and firmness (N/day) for fruit stored at different temperatures. The correlation coefficient (r) between the rates of decline in starch content and firmness is given.

(C) A nine-quadrant map showing metabolite accumulation affected by ethylene or CT in HY, with each dot representing a metabolite. The numbers indicate the accumulation patterns of metabolites across different quadrants: 1, higher at CT, lower under ethylene; 2, higher at CT, stable under ethylene; 3, higher both at CT and under ethylene; 4, stable at CT, lower under ethylene; 5, unclassified; 6, stable at CT, higher under ethylene; 7, lower both at CT and under ethylene; 8, lower at CT, stable under ethylene; 9, lower at CT, higher under ethylene.

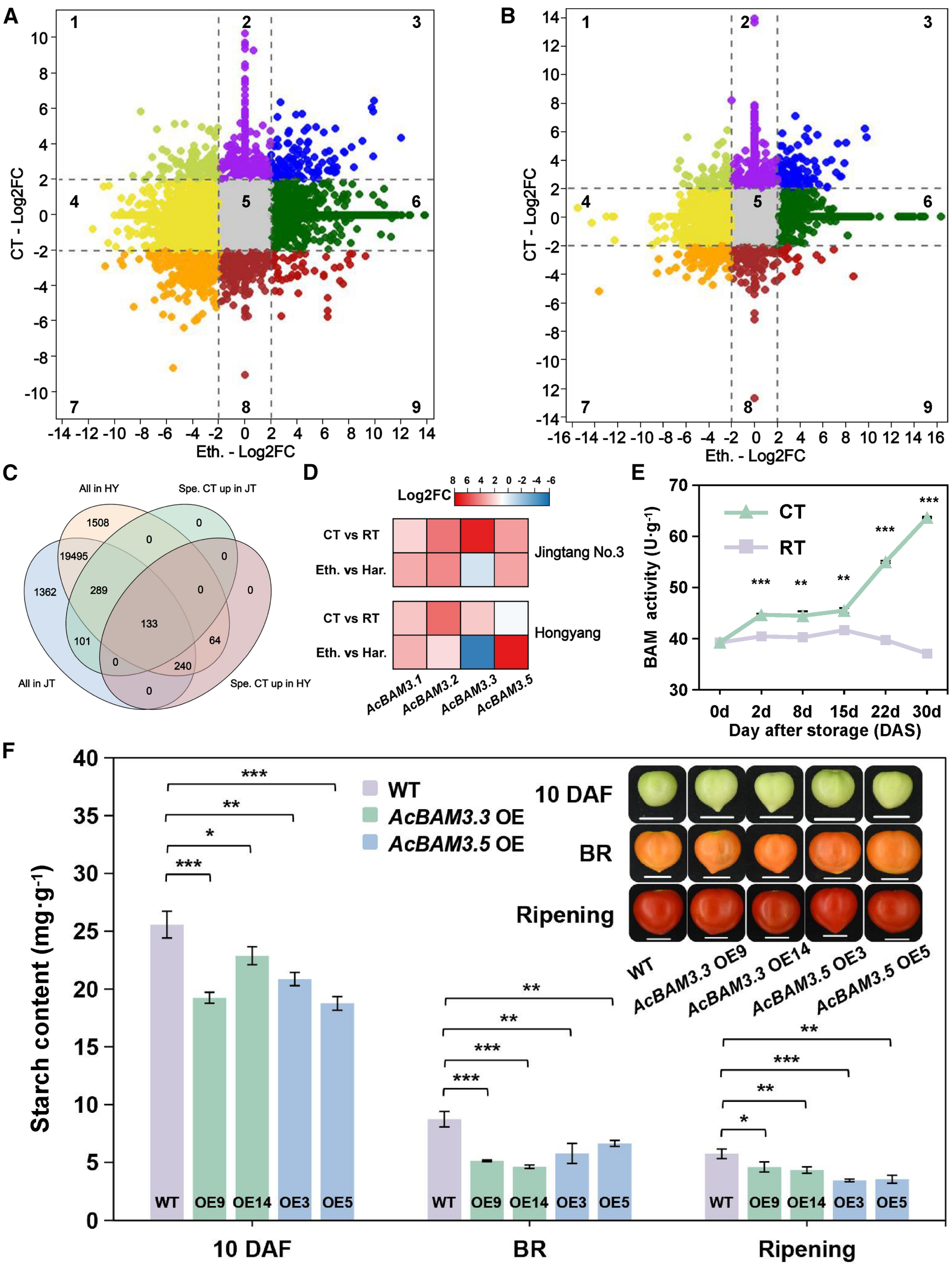

Figure 2. Transcriptome data and gene expression related to starch metabolism during kiwifruit ripening under cool temperature and ethylene treatment.

(A and B) Nine-quadrant association analysis of differentially expressed genes (DEGs) in 'Jintang No. 3' (JT) and 'Hongyang' (HY), respectively. The analysis cross-compares two independent sets of DEGs: cool temperature (CT) vs. room temperature (RT) and ethylene-treated vs. untreated control. Each dot represents a gene. Quadrants: 1, increased at CT but decreased under ethylene; 2, increased at CT and stable under ethylene; 3, increased both at CT and under ethylene; 4, stable at CT but decreased under ethylene; 5, unclassified; 6, stable at CT but increased under ethylene; 7, decreased both at CT and under ethylene; 8, decreased at CT but stable under ethylene; 9, decreased at CT but increased under ethylene.

(C) Venn diagram of CT-induced genes in JT and HY. As shown in quadrants 1 and 2 in **(A) and (B)**, these genes are upregulated at CT and either decrease or remain stable after ethylene treatment. Spe., specifically.

(D) Expression of four *AcBAM3* (BAM) genes at CT or RT. *Z* scores are standardized from −9 to 9. Har., harvest; Eth., ethylene treated.

(E) BAM activity in the outer pericarp of kiwifruit stored at CT or RT for 0–30 days.

(F) Starch degradation in 'Micro-Tom' tomatoes overexpressing *AcBAM3.3* and *AcBAM3.5*. DAF, days after flowering; BR, breaker. Top right: phenotypic appearance of wild-type (WT), *AcBAM3.3* overexpression (OE), and *AcBAM3.5* OE fruits; the scale bar represents 1 cm. Data are means ± SE of three replicates. Asterisks indicate significant differences as determined by Student's *t*-test (*$P < 0.05$, **$P < 0.01$, ***$P < 0.001$).

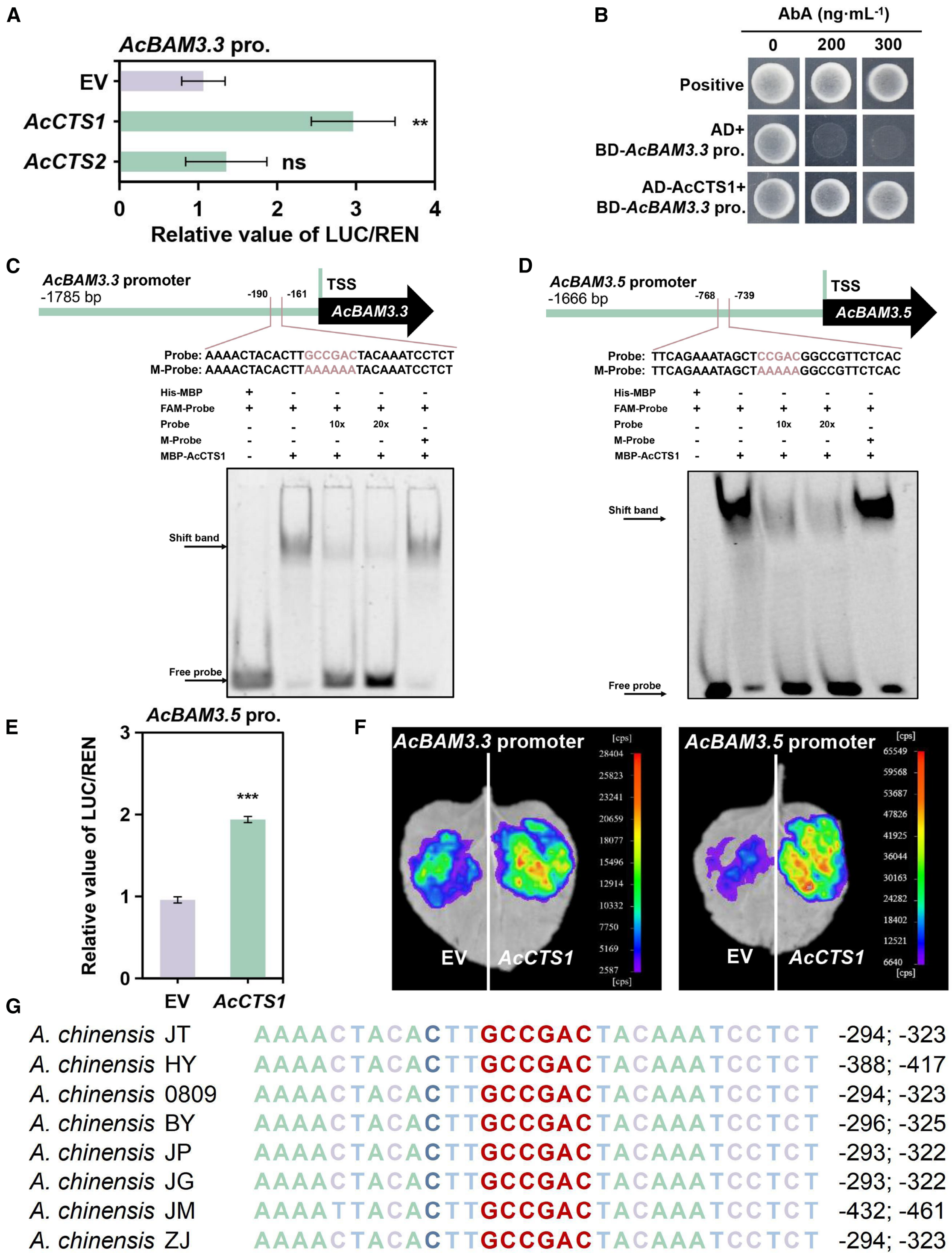

Figure 3. AcCTS1 activates the expression of *AcBAM3.3* and *AcBAM3.5*.

(A) Transactivation of the *AcBAM3.3* promoter by *AcCTS1* and *AcCTS2*. LUC/REN from the empty vector (EV) plus promoter–reporter was set to 1. Data represent means ± SE from three biological replicates. Student's *t*-test, $P < 0.01$. ns, not significant.

(B) Physical interaction of AcCTS1 with the *AcBAM3.3* promoter in a yeast one-hybrid (Y1H) assay. Growth of yeast cells co-transformed with different combinations of prey and bait on SD/−Ura/−Leu medium with 0, 200, and 300 ng ml^{-1} AbA is shown. pGADT7-AcCTS1 (AD-AcCTS1) was used as prey, and the pAbAi-*AcBAM3.3* promoter (BD-*AcBAM3.3* pro.) plasmid was used as bait. p53 was used as a positive control.

(C and D) Electrophoretic mobility shift assays (EMSAs) showing the *in vitro* binding of recombinant AcCTS1 to the promoters of *AcBAM3.3* and *AcBAM3.5*, respectively. The MBP-AcCTS1 fusion protein was incubated with synthesized FAM-labeled (FAM-Probe) or mutated probes (M-Probe), as

(legend continued on next page)

EV and the *AcBAM3.3* promoter in the presence of 200 and 300 ng ml^{-1} aureobasidin A (AbA). However, yeast cells expressing *AcCTS1* and the *AcBAM3.3* promoter grew well on medium containing 200 and 300 ng ml^{-1} AbA, indicating that *AcCTS1* directly interacts with the *AcBAM3.3* promoter.

The closest *Arabidopsis thaliana* homolog of *AcCTS1* (48.7% amino acid identity) was identified as *AtTINY2*, a member of the dehydration response element-binding protein subfamily of the APETALA2/ERF superfamily (Supplemental Figure 16). Analysis of *AcCTS1* expression revealed high constitutive levels in roots but minimal expression in stems, leaves, flowers, and throughout fruit development. In postharvest fruit, *AcCTS1* was strongly induced by CT but not by ethylene (Supplemental Figure 17), identifying it as a CTS transcriptional activator during ripening. This expression pattern was fully consistent with our transcriptomic data. To investigate whether AcCTS1 directly binds to specific DRE sites in the *AcBAM3.3* promoter, we performed electrophoretic mobility shift assays (EMSAs). Purified recombinant His-MBP-AcCTS1 directly bound to DRE-containing fragments derived from the *AcBAM3.3* promoter, causing clear mobility shifts, and the shifted bands weakened upon addition of increasing amounts of unlabeled WT probes but not mutated probes (Figure 3C).

EMSAs also demonstrated that AcCTS1 directly binds to the DRE motif of the *AcBAM3.5* promoter (Figure 3D). As shown in Figure 3E, the activity of the *AcBAM3.5* promoter increased 2.0-fold in the presence of *AcCTS1*, with a considerably higher LUC/REN ratio compared with that of the EV control. The transcriptional activation effect of AcCTS1 on the *AcBAM3.3/3.5* promoters was confirmed by LUC fluorescence imaging, in which fluorescence was observed after co-infiltration of the AcCTS1 effector and promoter reporters (Figure 3F). The AcCTS1 binding motif GCCGAC in the *AcBAM3.3* promoter was found to be conserved in different *Actinidia chinensis* (*A. chinensis)* cultivars (Figure 3G), suggesting that CT-responsive transcriptional regulation of *AcBAM3.3* may also occur in these genotypes.

AcCTS1 enhances cool temperature-induced starch degradation in kiwifruit

To investigate the role of AcCTS1 in starch degradation, we generated kiwifruit callus lines with stable OE or CRISPR–Cas9--mediated knockout of AcCTS1 (Figure 4A). Compared with WT calli, the OE lines showed 7.2-fold and 2.1-fold increases in *AcBAM3.3* and *AcBAM3.5* transcript levels, respectively (Figure 4B), accompanied by a 46% reduction in starch content ($P < 0.001$; Figure 4C). Using a CRISPR–Cas9 approach, we obtained two independent *cts1* mutant lines (#8 and #11), each harboring frameshift mutations in the *AcCTS1* open reading frame (Figure 4D). These mutations markedly reduced AcCTS1 protein accumulation (Figure 4E). In WT calli under CT, *AcBAM3.3* and *AcBAM3.5* expression levels increased by 5.1-fold and 1.2-fold, respectively ($P < 0.05$), but this response was largely abolished in the *cts1* mutants (Figure 4F). Correspondingly, starch content decreased by 25.0% in the WT (from 17.1 ± 1.3 to 12.9 ± 1.4 mg/g fresh weight) but remained unchanged in *cts1* lines (Figure 4G), supporting a critical role for *AcCTS1* in CT-mediated starch breakdown.

To confirm these findings in fruit, *AcCTS1* was transiently silenced or overexpressed in JT kiwifruit stored at CT (5°C; Figure 4H). RNAi-mediated silencing of *AcCTS1* led to marked reductions in AcCTS1 protein levels and expression of *AcBAM3.3* and *AcBAM3.5* at the RNAi-*AcCTS1* infiltration site (Figure 4I and 4J). This was accompanied by a significantly higher starch content ($P < 0.05$) compared with that of the EV control (Figure 4K). In addition, BAM enzyme activity and total sugar content were significantly reduced at the RNAi-*AcCTS1* infiltration site ($P < 0.01$; Figure 4L and 4M), indicating suppression of starch degradation and sugar accumulation. By contrast, OE of *AcCTS1* at CT resulted in upregulation of *AcBAM3.3/3.5*, enhancing starch degradation at the injection site, as evidenced by increased BAM activity and total sugar content (Supplemental Figure 18). Collectively, these results establish AcCTS1 as a central positive regulator of CT-induced starch degradation in kiwifruit.

The E3 ligase AcPUB11 interacts with and ubiquitinates AcCTS1 for proteasomal degradation

To identify regulators of AcCTS1 in response to CT, we performed a yeast two-hybrid (Y2H) screen using a cDNA library from the outer pericarp of CT-induced JT fruit (Supplemental Table 1). We identified an E3 ubiquitin ligase homologous to *Arabidopsis* AtPUB11 and designated it AcPUB11 (Acc09233). Yeast cells co-transformed with AcCTS1 and AcPUB11 turned blue in the presence of the chromogenic substrate 5-bromo-4-chloro-3-indolyl α-D-galactopyranoside (X-α-Gal), as did the positive control, whereas the negative controls did not (Figure 5A), indicating that AcCTS1 interacts with AcPUB11 in yeast cells. This interaction was further supported by a LUC complementation imaging (LCI) assay *in planta*. Luciferase activity was detected in *N. benthamiana* leaves co-expressing AcCTS1-nLUC and cLUC-AcPUB11 (Figure 5B), but no LUC activity was detected in the negative control combinations. Bimolecular fluorescence complementation (BiFC) assays confirmed this interaction in the nucleus (Figure 5C). AcCTS1 was observed to localize exclusively to the nucleus at both RT and CT in *N. benthamiana* leaves (Supplemental Figure 19), demonstrating that its nuclear localization is constitutive rather than temperature dependent. Glutathione S-transferase (GST) pull-down and

indicated. Unlabeled probes were used as competitors. −, absence; +, presence. The position of each probe relative to the transcription start site (TSS) is indicated above the panels.

(E) Transactivation of the *AcBAM3.5* promoter by AcCTS1. LUC/REN from the EV plus promoter–reporter was set to 1. Data represent means ± SE from three biological replicates. Student's *t*-test, $P < 0.001$.

(F) LUC bioluminescence imaging of *N. benthamiana* leaves co-transformed with 35S::AcCTS1 and pGreenII0800-LUC reporters driven by the *AcBAM3.3* and *AcBAM3.5* promoters.

(G) DRE element alignment of the *AcBAM3.3* promoter in the *A. chinensis* genome. JT, HY, 0809, BY, JP, JG, JM, and ZJ represent the 'Jintang No. 3,' 'Hongyang,' 'Hongyang 0809,' 'Biyu,' 'Jinpai,' 'Hort16a,' 'Jinmi,' and 'Zhejiang' cultivars or species. The numbers on the right represent the positions on the promoters. The sequence was downloaded from https://kiwifruitgenome.atcgn.com/.

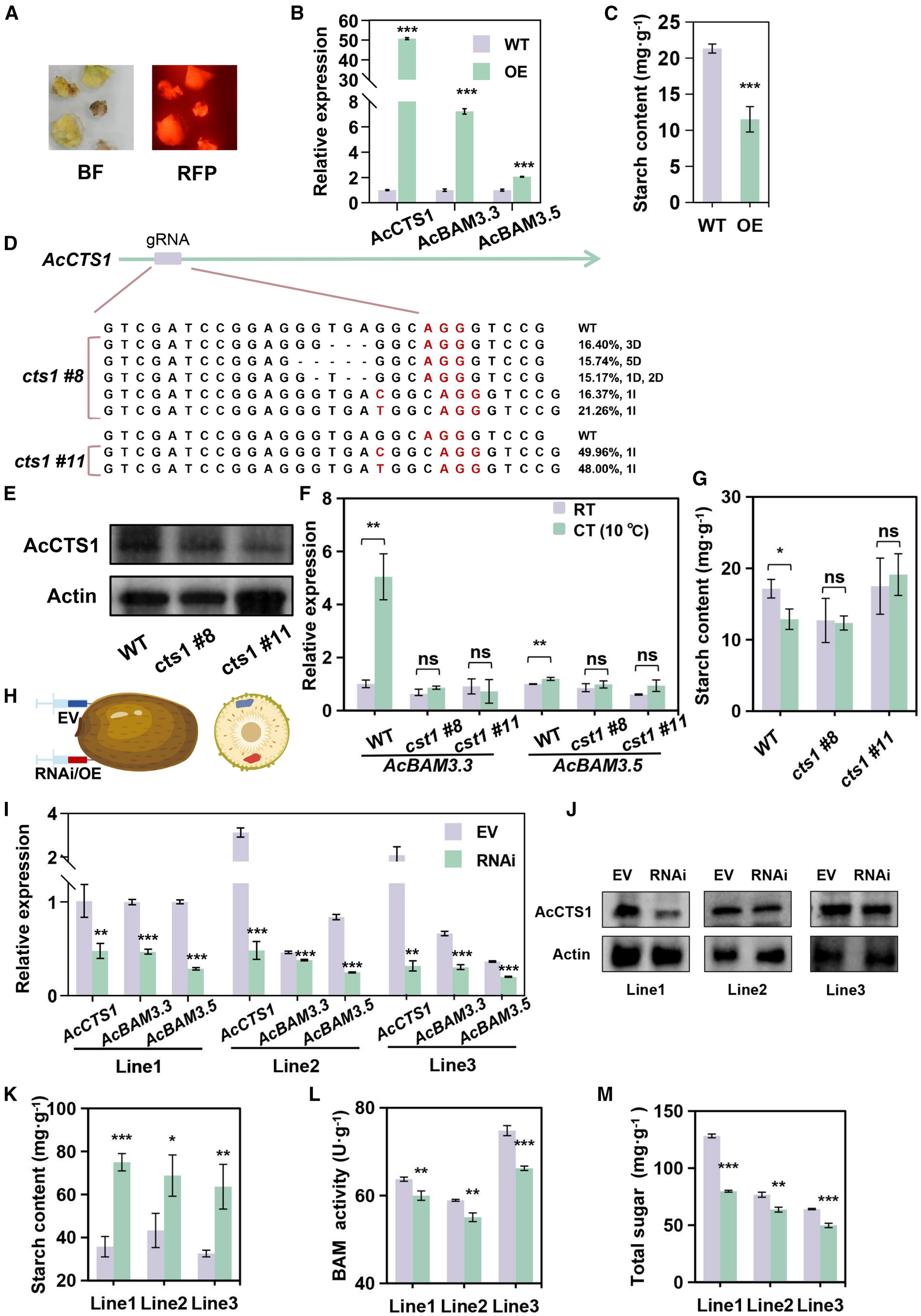

Figure 4. AcCTS1 controls cool temperature-induced starch degradation in kiwifruit.
(A–C) OE of AcCTS1 in HY calli promotes starch degradation.
(A) Morphology of WT and *AcCTS1*-OE calli under bright-field (BF) and red fluorescent protein (RFP) light sources.
(B) Expression analysis of *AcCTS1*, *AcBAM3.3*, and *AcBAM3.5* in WT and *AcCTS1*-OE calli.

(legend continued on next page)

co-immunoprecipitation (coIP) assays also confirmed the interaction *in vitro*. In the GST pull-down assay, recombinant GST-AcPUB11, but not GST alone, was pulled down by maltose-binding protein (MBP)-AcCTS1 (Figure 5D). In the coIP assay, GFP-AcPUB11 immunoprecipitated hemagglutinin (HA)-AcCTS1, but not GFP-GUS, when an anti-GFP antibody was used for IP (Figure 5E). Together, these results demonstrate the interaction of AcCTS1 with AcPUB11 *in vitro* and in *vivo*.

Given the role of E3 ligases in protein degradation, we quantitatively analyzed AcPUB11 and AcCTS1 protein levels by western blotting using fruit ripened at CT (5°C) or RT. A reduction in AcPUB11 abundance and an increase in AcCTS1 protein levels were observed in the outer pericarp of kiwifruit ripened at CT compared with RT, indicating a negative correlation between AcPUB11 and AcCTS1 protein levels (Supplemental Figure 20A). Consistent with the observations in JT, storage at CT also led to a reduction in AcPUB11 protein levels and a concurrent increase in AcCTS1 in HY and Jianxiang (Supplemental Figure 20B and 20C). This reproducible pattern across three genetically distinct cultivars suggests that posttranslational regulation within the AcPUB11–AcCTS1 module may be a consistent and reliable feature of the CT response in commercial kiwifruit. To determine whether the reduced abundance of AcPUB11 protein at CT is regulated at the transcriptional level, we analyzed *AcPUB11* mRNA expression. In contrast to the protein accumulation pattern, RT–qPCR analysis revealed that *AcPUB11* transcript levels were significantly upregulated, showing a 2- to 3-fold increase in kiwifruit stored at CT compared with RT (Supplemental Figure 21). This clear divergence between transcript and protein levels indicates that the downregulation of AcPUB11 at CT is governed primarily by posttranscriptional and/or posttranslational mechanisms rather than by transcriptional repression. This result suggests that AcPUB11-mediated proteasomal degradation of AcCTS1 is attenuated under CT.

AcPUB11 ubiquitination of AcCTS1 was assessed by an *in vitro* ubiquitination assay using purified recombinant AcCTS1-HA and AcPUB11-Myc. Ubiquitination of AcCTS1-HA was detected in the presence of AcPUB11-Myc, ubiquitin, an E1 ubiquitin-activating enzyme, and an E2 ubiquitin-conjugating enzyme, as shown by higher-molecular-weight bands that were not observed in the absence of AcPUB11-Myc (Figure 5F). We also examined the ubiquitination of AcCTS1 by AcPUB11 *in vivo* by co-expressing HA-AcCTS1 and GFP-AcPUB11 constructs in *N. benthamiana* leaves. As shown in Figure 5G, polyubiquitinated HA-AcCTS1 was present in significantly higher quantities when HA-AcCTS1 was co-expressed with GFP-AcPUB11 than with the GFP-GUS control vector only, indicating that AcPUB11 can promote AcCTS1 ubiquitination.

To further examine whether AcPUB11 mediates degradation of AcCTS1 via the 26S proteasome pathway, we transiently co-expressed HA-AcCTS1 and AcPUB11 in *N. benthamiana* leaves. Immunoblotting with an anti-HA antibody revealed that AcCTS1 abundance declined markedly in the presence of AcPUB11 compared with the GUS control, and this decline was inhibited by MG132, a 26S proteasome inhibitor (Figure 5H). These results demonstrate that AcPUB11 ubiquitinates AcCTS1 and facilitates its degradation via the 26S proteasome pathway.

AcPUB11 attenuates AcCTS1-mediated activation of *AcBAM3.3/3.5* and starch degradation

Given that AcPUB11 mediated the ubiquitination and proteasomal degradation of AcCTS1, we hypothesized that AcPUB11 might interfere with the AcCTS1-induced transactivation of *AcBAM3.3* and *AcBAM3.5*. To test this possibility, we performed transient OE assays in *N. benthamiana* leaves using a dual-luciferase reporter system. Expression of *AcCTS1* significantly activated LUC activity driven by the *AcBAM3.3* and *AcBAM3.5* promoters; however, this activation was markedly suppressed in the presence of AcPUB11 (Figure 6A). The repression by AcPUB11 was significantly reduced when MG132 was included in the reaction mixture (Figure 6A), suggesting proteasome-dependent degradation of AcCTS1. These results were further supported by LUC fluorescence imaging, which showed that AcPUB11 abolished AcCTS1-induced promoter activity and that this activity was recovered upon co-application of MG132 (Figure 6B).

To further investigate the role of AcPUB11 in regulating starch degradation, we generated *pub11* mutant lines in kiwifruit calli using CRISPR-Cas9. Two independent lines (#14 and #15) carried frameshift mutations in the *AcPUB11* open reading frame (Figure 6C) and exhibited significantly reduced AcPUB11 protein levels accompanied by increased AcCTS1 accumulation compared with the WT (Figure 6D). In *pub11* mutant lines, *AcBAM3.3* and *AcBAM3.5* transcript levels increased by at least 1.7- and 1.9-fold, respectively, compared with those in

(C) Starch content in WT and *AcCTS1*-OE calli.
(D–G) CRISPR-generated *cts1* mutants show delayed starch degradation under CT in HY calli.
(D) Schematic of target sites in *AcCTS1* and Hi-TOM sequencing of edited sites in the *cts1* mutant #8 and #11 lines. The target sites in the *AcCTS1* genomic sequence are shown, with the protospacer adjacent motif (PAM) sequence highlighted in red. Nucleotide deletions (D) are indicated by dashes (–), and insertions (I) are marked with the respective base letters in red. Percentages indicate the frequency of each specific mutant allele identified by high-throughput sequencing (Hi-TOM) of the pooled mutant lines.
(E) Immunoblot analysis of AcCTS1 in WT and *cts1* lines; actin was used as the loading control.
(F and G) *AcBAM3.3* and *AcBAM3.5* expression and starch content in the WT and two *cts1* mutant lines at RT and CT.
(H–M) Transient silencing of *AcCTS1* inhibits CT-induced starch degradation in kiwifruit.
(H) Schematic of transient transgenic injection in kiwifruit.
(I) Expression of *AcCTS1*, *AcBAM3.3*, and *AcBAM3.5* at the injection site in kiwifruit.
(J) Immunoblot of EV- and AcCTS1-RNAi-injected fruit; actin was used as the loading control.
(K–M) Quantification of starch content, β-amylase (BAM) activity, and total sugar content at the injection sites. Data represent means ± SE from three biological replicates. Asterisks indicate significant differences as determined by Student's *t*-test (*$P < 0.05$, **$P < 0.01$, ***$P < 0.001$).

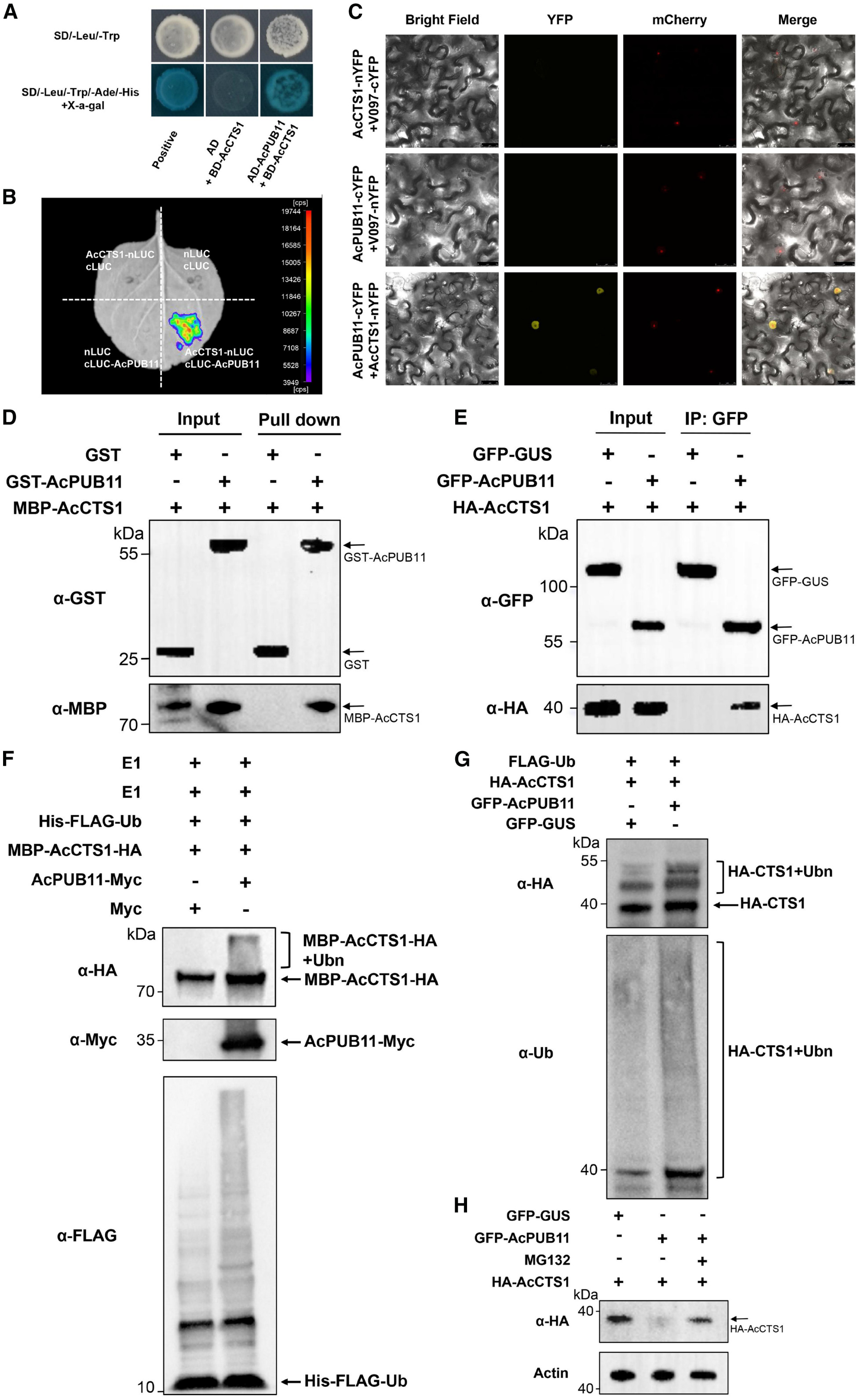

Figure 5. AcPUB11 interacts with and ubiquitinates AcCTS1 for proteasomal degradation.

(A) Yeast two-hybrid (Y2H) assay showing the interaction between AcPUB11 and AcCTS1. Yeast cells cotransformed with BD-AcCTS1 and AD-AcPUB11 were grown on SD medium (lacking Leu, Trp, Ade, and His) in the presence of the chromogenic substrate 5-bromo-4-chloro-3-indolyl α-D-galactopyranoside (X-α-Gal).

(legend continued on next page)

the WT ($P < 0.05$; Figure 6E), and starch content decreased by 20.4% and 19.4% in lines #14 and #15, respectively ($P < 0.05$; Figure 6F), confirming a repressive role for AcPUB11 in starch degradation.

We next performed transient OE and silencing of *AcPUB11* in kiwifruit stored at CT (5°C) to evaluate *AcPUB11*function *in vivo* (Figure 6G). OE of *AcPUB11* led to increased transcript and protein levels, which suppressed *AcBAM3.3/3.5* expression, increased starch content, and reduced BAM activity and total sugar content at the injection site (Figure 6H–6L). Conversely, RNAi-mediated silencing of *AcPUB11* reduced AcPUB11 abundance, increased AcCTS1 protein levels and *AcBAM3.3/3.5* expression, and enhanced starch degradation, as indicated by lower starch content and higher BAM activity and total sugar content (Supplemental Figure 22). These results demonstrate that AcPUB11 attenuates AcCTS1-mediated activation of *AcBAM3.3/3.5* and inhibits CT-induced starch degradation through proteasomal degradation of AcCTS1.

DISCUSSION

Cool temperature-dependent kiwifruit ripening behavior: A shared “after-harvest” and “on-the-vine” phenomenon

Kiwifruit ripening after harvest occurs in four phases (Atkinson et al., 2011). Entry into phase 1 ripening is associated with the initiation of starch degradation and has often been attributed to basal levels of system I (wound) ethylene production associated with fruit harvest (Jabbar and East, 2016). Here, we show that significant ripening occurs in kiwifruit after harvest in a system designed to preclude wound or pathogen-induced ethylene production. Moreover, we show that changes in softening, starch degradation, and SSC occur more rapidly at CTs (5°C–10°C) than at RT (20°C) or cold temperature (1°C). Critically, these changes occurred under conditions in which ethylene perception was blocked by 1-MCP, demonstrating the existence of an ethylene-independent ripening pathway. These CT-induced ripening changes were observed in JT, HY, and ‘Cuixiang’ (CX) kiwifruit and confirm previous observations in other kiwifruit cultivars (e.g., Asiche et al., 2017; Burdon et al., 2017; Gunaseelan et al., 2019). Comprehensive metabolomic and transcriptomic studies revealed that CT-induced ripening pathways operate in kiwifruit (Figures 1 and 2). Notably, a BAM gene (*AcBAM3.3*) that could account for CTS-induced starch degradation was identified and characterized.

Kiwifruit ripening on the vine also occurs in the absence of detectable ethylene production (e.g., Murakami et al., 2015; Burdon et al., 2017). On-vine ripening has been proposed to occur in response to environmental cues, such as changes in daylength, light quality, and cooling temperatures in autumn (Ngcobo et al., 2022; Qiu et al., 2024). Our results show that fruits ripen after harvest specifically in response to CTs—the same temperatures that kiwifruit vines would sense during autumn in the field. Understanding how fruit perceive and respond to these temperatures is important for fruit growth and the management of fruit production in response to global climate change. In particular, warmer autumns may reduce fruit flavor owing to lower sugar accumulation and increase the risk of pests and diseases, as the active period of pathogen infection is extended. The AcPUB11–AcCTS1–AcBAM3s module identified in this study provides a rational genetic basis on which kiwifruit with greater resilience to climate change could be selected.

AcCTS1 is a novel cool temperature-dependent ERF involved in the modulation of starch degradation

Transcriptomic correlation analysis identified 15 CTS TFs in kiwifruit, including members of the NAC, bZIP, and MYB families (Supplemental Figure 15). AcCTS1, the TF most highly positively correlated with starch degradation and *AcBAM3.3* expression, was shown to bind to the promoters of *AcBAM3.3* and *AcBAM3.5* (Figure 3) and trigger starch degradation in response to CT. AcCTS1 is an ERF-family TF that shows the highest identity to TINY ERFs, which have previously been associated with resistance to abiotic stresses such as drought, cold, and salt (Sun et al., 2008; Coego et al., 2014; Shi et al., 2022). Notably, *AcCTS1* specifically responds to CT but not to ethylene, expanding our understanding of ERFs associated with starch degradation by revealing a regulator dedicated to the

(B) Luciferase complementation imaging assay in *N. benthamiana* leaves showing the interaction between AcPUB11 and AcCTS1. AcCTS1-nLUC was co-expressed with cLUC-AcPUB11; AcCTS1-nLUC/cLUC, nLUC/cLUC-AcPUB11, and nLUC/cLUC were used as negative controls. Luciferase activity was recorded with a charge-coupled device camera. Representative images of *N. benthamiana* leaves at 60 h after infiltration are shown.

(C) Bimolecular fluorescence complementation (BiFC) assay of the interaction between AcPUB11 and AcCTS1. AcCTS1-nYFP and cYFP-AcPUB11 were co-expressed in *N. benthamiana* leaves, with mCherry as a nuclear signal. AcCTS1-nYFP/cYFP-V097, V097-nYFP/cYFP-AcPUB11, and nYFP/cYFP were used as negative controls. Images at different emission wavelengths, the bright field, and the overlay are shown. V097 is a 60-bp multiple cloning fragment. Scale bars are indicated.

(D) *In vitro* GST pull-down assay showing the interaction between AcPUB11 and AcCTS1. Recombinant MBP-AcCTS1 was incubated with GST-AcPUB11 or GST, and the bound proteins were detected by immunoblotting using anti-GST or anti-MBP antibodies.

(E) *In vivo* coIP assay showing the interaction between AcPUB11 and AcCTS1. GFP-GUS and HA-CTS1 and GFP-AcPUB11 and HA-CTS1 were transiently expressed in *N. benthamiana* leaves. Proteins were immunoprecipitated using GFP-tagged magnetic beads. Input and IP fractions were analyzed by immunoblotting with anti-GFP and anti-HA antibodies. Molecular weight markers (kDa) are shown on the left of each blot.

(F) *In vitro* ubiquitination assay showing that AcPUB11 ubiquitinates AcCTS1. The activity of recombinant AcPUB11-Myc in AcCTS1-HA ubiquitination was tested in the presence and absence of AcPUB11-Myc. Ubiquitinated AcCTS1-HA was detected by immunoblotting with an anti-HA antibody.

(G) *In vivo* ubiquitination of AcCTS1 mediated by AcPUB11. Total protein was extracted from *N. benthamiana* leaves transiently expressing FLAG-ubiquitin (Ub), HA-AcCTS1, and GFP-AcPUB11 or GFP-GUS (negative control). Ubiquitinated AcCTS1 was detected using anti-HA and anti-ubiquitin antibodies.

(H) Proteasome-mediated degradation assay of AcCTS1 mediated by AcPUB11 in plant cells. HA-AcCTS1 was co-expressed with GFP-AcPUB11 or GFP-GUS in *N. benthamiana* leaves in the presence or absence of MG132. The abundance of AcCTS1 was analyzed by immunoblotting with an anti-HA antibody. Actin served as the loading control. Molecular weight markers (kDa) are shown on the left of each blot.

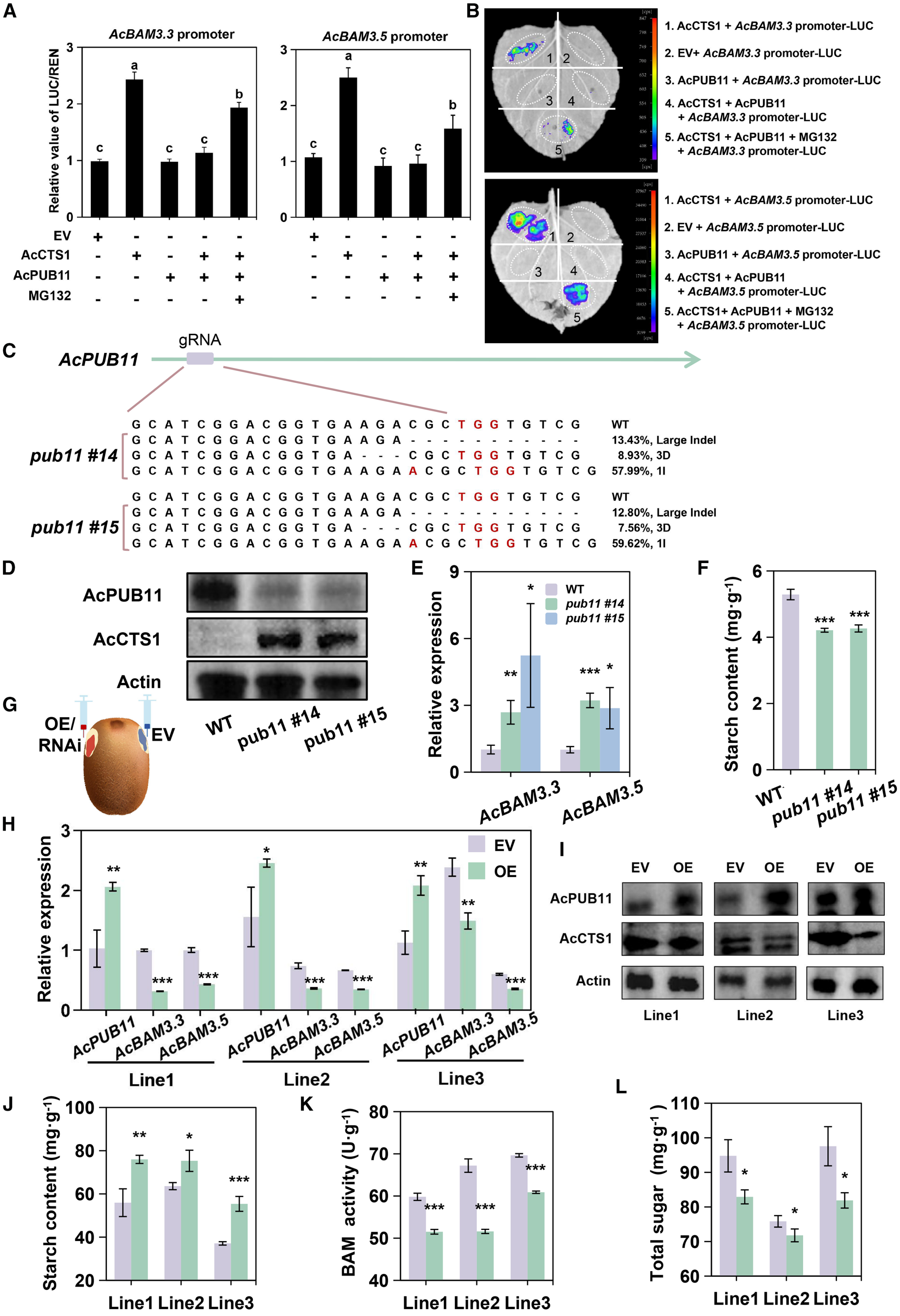

Figure 6. Attenuation of AcCTS1-induced starch degradation by AcPUB11.

(A and B) Dual LUC activation assay **(A)** and LUC bioluminescence imaging **(B)** show that AcPUB11 suppresses AcCTS1-mediated transactivation of the *AcBAM3.3* and *AcBAM3.5* promoters. LUC driven by *AcBAM3* promoters (AcBAM3.3pro:LUC and AcBAM3.5pro:LUC) and REN driven by the CaMV 35S promoter (as an internal control) in the same vector were co-expressed with effector plasmids expressing AcCTS1 or AcPUB11 in *N. benthamiana* leaves.

(legend continued on next page)

ethylene-independent pathway. Many TFs are involved in starch degradation in response to ethylene in banana; these include ERFs (Song et al., 2025a, 2025b; Jiang et al., 2025), as well as basic-helix–loop–helix (bHLH), EIL, MYBs, ARFs, MADSs, and NACs (Xiao et al., 2018; Song et al., 2019; Miao et al., 2020; Jiang et al., 2021; Liu et al., 2021; Zhu et al., 2021; Wei et al., 2023b). ERFs have been shown to modulate the transcription of various ripening-related genes, including those involved in ethylene biosynthesis, fruit softening, chlorophyll catabolism, and carotenoid synthesis (Xiao et al., 2013; Han et al., 2016; Yin et al., 2016; Dang et al., 2021; Cui et al., 2024). Other AcCTS ERFs, as well as members of other TF families (Supplemental Figure 15), may be involved in regulating these ripening processes in kiwifruit.

Posttranslational modifications influence cool temperature-specific starch degradation

Ubiquitination is a critical posttranslational modification that affects the stability or abundance of TFs; it is involved in regulating various fruit quality traits, including color formation. For example, the apple E3 ligase MdMIL1 catalyzes the degradation of MdMYB308L, negatively regulating anthocyanin accumulation (An et al., 2020a), and the banana E3 ligase MaBAH1 ubiquitinates MaMYB60, attenuating MaMYB60-induced transactivation of chlorophyll catabolic genes (Wei et al., 2023a). However, the role of E3 ligases in starch degradation regulation in fruit remains largely unknown. Our study shows that AcPUB11 attenuates AcCTS1 activation of *AcBAM3.3*/*3.5* promoters, with MG132 treatment partially rescuing the activation of *AcBAM3s* by AcCTS1 (Figure 6A and 6B). Transient OE and silencing of AcPUB11 demonstrated its negative regulation of *AcBAM3* expression and starch degradation in the outer pericarp of kiwifruit (Figure 6H–6L and Supplemental Figure 22). Ubiquitination is also involved in plant stress responses; it has been extensively studied under stress conditions, mainly drought and salinity, but less is known about its role in response to temperature stress. Plants often adapt to stress through ubiquitination of proteins, promoting plant growth and fruit development. For example, the grapevine E3 ligase VviPUB19 regulates the stability of inducer of CBF expression (ICE) and CBF TFs to affect cold tolerance (Wang et al., 2024), and the tomato E3 ligase SlCHIP plays a key role in heat stress responses by targeting misfolded proteins produced during heat stress for degradation (Zhang et al., 2021). Our study reveals that the E3 ubiquitin ligase AcPUB11 regulates starch degradation in response to the CT-induced TF AcCTS1, a mechanism that differs from previously reported temperature stress responses (An et al., 2020b; Wei et al., 2023a; Wang et al., 2024).

In practice, CT-induced ripening holds considerable potential for industrial applications because it promotes ripening independently of ethylene, extending the edible window and reducing the risk of over-ripening during distribution. Several strategies can further shorten the CT-based ripening period to meet commercial timelines. For example, initiating CT treatment at a lower initial firmness can substantially reduce the time required to reach the ready-to-eat stage. Alternatively, a fluctuating temperature strategy (25°C for 12 h followed by 10°C) can accelerate softening to approximately 8 days while maintaining high eating quality (Chen et al., 2025). These findings demonstrate that CT-based ripening can be translated into a practical and controllable postharvest technology for ready-to-eat kiwifruit.

A model depicting the molecular basis of cool temperature-induced starch degradation under ethylene inhibition in kiwifruit

Our findings reveal a temperature-responsive, ubiquitination-controlled transcriptional regulatory module that governs ethylene-independent starch degradation in kiwifruit (Figure 7). This model delineates the regulatory mechanism that operates when ethylene signaling is suppressed. At RT (20°C), AcPUB11 interacts with and ubiquitinates the CTS factor AcCTS1, leading to its proteasomal degradation and repression of *AcBAM3.3* and *AcBAM3.5* expression, resulting in minimal starch degradation. At CT (5°C–10°C), reduced AcPUB11 abundance leads to an increase in AcCTS1 protein levels. This enhances the AcCTS1-mediated activation of *AcBAM3.3* and *AcBAM3.5*, promoting starch degradation and increasing sugar content. This AcPUB11–AcCTS1–AcBAM3s regulatory module provides a molecular explanation for kiwifruit ripening under CT conditions.

METHODS

Plant materials

Yellow-fleshed JT and 'Jianxiang' (*A. chinensis* var. *chinensis* Planch.) were collected from Enshi, Hubei Province; red-fleshed HY (*A. chinensis* var. *chinensis*) from Yuxi, Yunnan Province; and green-fleshed 'Cuixiang' (*A. chinensis* var. *deliciosa* A. Chev.) from Xi'an, Shaanxi Province.

The LUC/REN ratio of the EV + AcBAM3spro:LUC reporter was set to 1. Data are means ± SE from three biological replicates. Different lowercase letters indicate significant differences as determined by one-way ANOVA followed by Tukey's test ($P < 0.05$).

(C–F) *pub11* mutant HY calli show enhanced starch degradation.

(C) Schematic of target sites in the *AcPUB11* genomic sequence and Hi-TOM sequencing of edited sites in *pub11* mutant lines #14 and #15. The target sites in the *AcPUB11* genomic sequence are shown, with the PAM sequence highlighted in red. Nucleotide deletions (D) are denoted by dashes (–) and insertions (I) by the corresponding base letters in red. Percentages represent the editing efficiency, defined as the proportion of sequencing reads that contained insertions or deletions at the target site for each independent *pub11* mutant line.

(D) Immunoblot analysis of AcPUB11 and AcCTS1 in WT and *pub11* mutants; actin was used as the loading control.

(E and F) *AcBAM3.3* and *AcBAM3.5* expression and starch content in the WT and *pub11* mutants.

(G–L) Transient OE of *AcPUB11* in kiwifruit at CT inhibits starch degradation.

(G) Schematic of transient *AcPUB11* OE/RNAi in kiwifruit.

(H) Expression of *AcPUB11*, *AcBAM3.3*, and *AcBAM3.5* at the injection sites in kiwifruit.

(I) Immunoblot analysis of AcPUB11-OE and EV controls. Actin was used as the loading control.

(J–L) Quantification of starch content, BAM activity, and total sugar content at the injection sites. Data are means ± SE from three biological replicates. Asterisks indicate significant differences as determined by Student's *t*-test (*$P < 0.05$, **$P < 0.01$, ***$P < 0.001$).

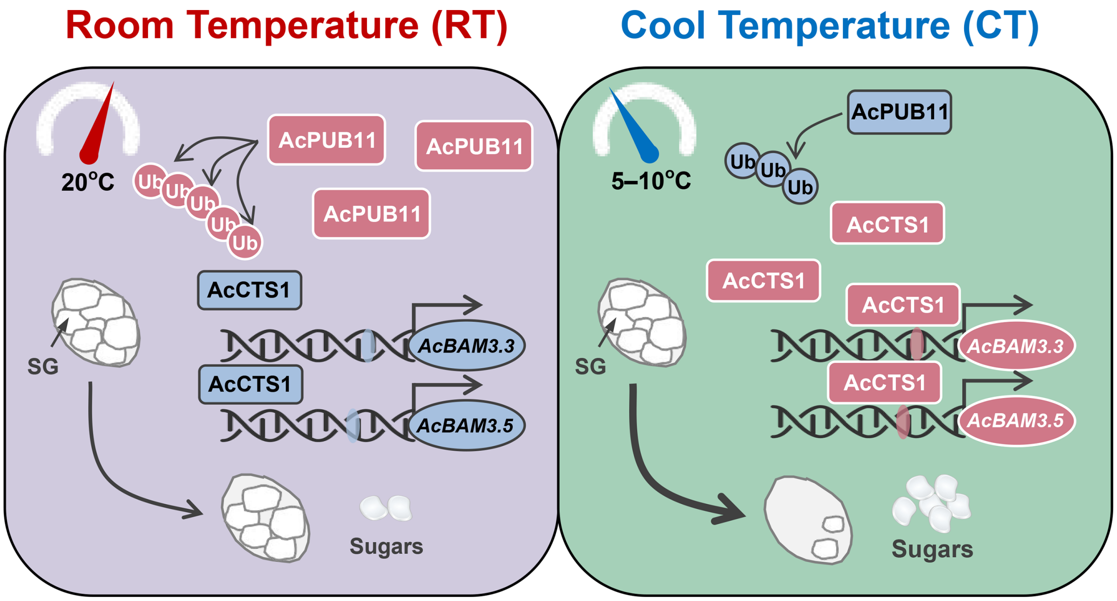

Figure 7. Proposed regulatory network of cool temperature-induced starch degradation in kiwifruit.
At RT, AcPUB11 interacts with and ubiquitinates AcCTS1, leading to the proteasomal degradation of AcCTS1; this represses AcCTS1-mediated induction of *AcBAM3.3* and *AcBAM3.5*, resulting in stable starch content in the fruit. When kiwifruit are ripened at CT, reduced temperature decreases AcPUB11 abundance, thereby increasing AcCTS1 abundance. Consequently, AcCTS1 directly targets and activates the expression of *AcBAM3.3* and *AcBAM3.5*, leading to starch degradation and a subsequent increase in fruit sugar content. SG, starch granule.

All fruit samples were collected ~150 days after flowering, with an SSC of ~6.5°Bx. Healthy fruit of uniform size that showed no physical damage were soaked for 2 min in a fungicide solution of 100 μl l^{-1} imazalil and 100 μl l^{-1} prochloraz for sterilization. Afterward, fruit were dried to remove surface moisture.

Temperature and ethylene treatments

For temperature treatment, 500 fruit were individually placed in 0.01-mm-thick plastic bags containing a 0.5 × 0.5-cm paper sheet impregnated with 1-MCP (SmartFresh, Philadelphia, PA, USA) at a concentration of 0.75 μl l^{-1}. Fruit were then randomly divided into 5 groups and stored at constant temperatures of 1°C, 5°C, 10°C, 15°C, and 20°C (254LMIR-154 incubator with a rotating fan; Sanyo, Osaka, Japan). Ethylene production was monitored using a GC7890B gas chromatograph (Agilent Technologies, Santa Clara, CA, USA) as described previously (Hu et al., 2024) prior to each physiological measurement. Fruits were grouped into sets of three for measurement, and groups with detectable ethylene were immediately removed. Physiological measurements were taken at 0, 2, 8, 15, 22, and 30 days, with 4–6 fruits sampled for each temperature at each time point. This experiment was repeated three times.

For ethylene treatment, fruit were placed in a 400-l gas-tight box equipped with a fan for air circulation. Ethylene gas was injected into the box at a concentration of 10 μl l^{-1} for 12 h. Fruit were then stored at 20°C, and physiological indices were measured 0.5, 1, 2, 3, 5, 7, 9, and 11 days post-treatment. This experiment was performed 3 times with at least 10 fruits sampled per time point.

Fruit physiological measurements

A 1-mm slice of kiwifruit skin was peeled before firmness was measured using a digital fruit firmness tester (GY-4, China Aipu Metrology Instrument, Quzhou, Zhejiang, China) with a 7.9-mm-diameter probe (Zhao et al., 2021). Two measurements were taken per fruit. SSC was measured using a handheld refractometer (ATAGO, Tokyo, Japan) and expressed as degrees Brix, with three measurements per fruit. Outer pericarp tissue was ground to a fine powder using liquid nitrogen and stored at −80°C for further analysis. Commercial test kits were used to measure starch content (AKSU015M, Beijing Boxbio Science & Technology, China), total sugar content (AKSU003M, Beijing Boxbio Science & Technology), and amylase activity (R32600, Shanghai Uuanye Bio-Technology, China) according to the manufacturer's instructions. All measurements used three biological replicates.

RNA extraction, cDNA synthesis, RT–qPCR, and immunoblot analysis

Total RNA was extracted from the outer pericarp of kiwifruit using the HiPure HP Plant RNA Mini Kit (Magen, Guangzhou, China). The quality of the extracted RNA was assessed using a NanoDrop spectrophotometer (Thermo Fisher Scientific, Waltham, MA, USA), and RNA integrity was analyzed by 1% agarose gel electrophoresis. cDNA was synthesized by reverse transcription using the HiScript III 1st Strand cDNA Synthesis Kit (Vazyme, Nanjing, China). RT–qPCR was performed on a Roche LightCycler 480 instrument using ChamQ Universal SYBR qPCR Master Mix (Vazyme) following the manufacturer's instructions. The expression of kiwifruit actin was used as the reference. The primers used are listed in Supplemental Table 2.

For immunoblot analysis, anti-AcCTS1 and anti-AcPUB11 polyclonal antibodies were affinity purified from rabbit antisera by AtaGenix Biotechnology (Wuhan, China). Total proteins were extracted using radio immunoprecipitation assay lysis buffer (high) (Solarbio, Beijing, China) according to the manufacturer's instructions. Total proteins were denatured at 95°C for 10 min by addition of 1× SDS loading buffer and then separated by SDS–PAGE (ACE, Changzhou, China). After electrophoresis, proteins were electrotransferred onto a 0.45-μm nitrocellulose membrane (Thermo Fisher Scientific, USA) using a Trans-Blot SD semi-dry electrophoretic transfer system (Bio-Rad, USA). Immunoblot analysis was performed using anti-AcCTS1 or anti-AcPUB11 antibodies. Polyclonal antibodies against AcCTS1 and AcPUB11 were generated in rabbits by AtaGenix Laboratories. For each antibody, the full-length recombinant protein was expressed in

Escherichia coli (*E. coli*) and purified to serve as the immunogen. The resulting antisera were then affinity purified against the respective recombinant antigens to ensure specificity.

RNA sequencing and metabolome analysis

RNA sequencing was performed by Majorbio Biotechnology (Shanghai, China) with three biological replicates per condition. Libraries were generated from outer pericarp tissues of JT and HY kiwifruit using the NEBNext Ultra RNA Library Prep Kit (Illumina) according to the manufacturer's protocols. The analyzed samples included fruit from JT and HY at harvest (day 0) and after 2 days of ethylene treatment; JT fruit stored at 20°C and 5°C for 8 days; and HY fruit stored at 10°C and 20°C for 15 days. Sequencing was performed on the Illumina NovaSeq 6000 platform. Raw paired-end reads were quality controlled and adapter trimmed using fastp (v.0.23.4) with default parameters. Cleaned reads were aligned to the kiwifruit Red5 genome (Pilkington et al., 2018) using HISAT2 (v.2.2.1). Transcript abundance was quantified using RSEM (RNA-Seq by Expectation-Maximization, v1.3.3). Differential expression analysis was performed using DESeq2 (v.1.30.1) in R, with genes satisfying $|\log_2(\text{fold change})| > 1$ and false discovery rate–adjusted $P < 0.05$ considered to be differentially expressed. The metabolome analysis was performed as described in Zeng et al. (2025). Outer pericarp tissues of HY were collected at harvest (day 0), after 15 days of storage at 10°C or 20°C, and at 2 days after ethylene treatment. These tissues were used for metabolome analysis via widely targeted high performance liquid chromatography (HPLC)–tandem mass spectrometry (MS/MS).

Dual-luciferase transient expression assays

The full-length coding sequences (CDSs) of *AcCTS* TFs were cloned into the pKlic1.0 vector to generate effectors. The 1785-bp *AcBAM3.3* and 1666-bp *AcBAM3.5* promoters were cloned into the pGreenII0800-LUC vector to generate reporters. The primers used are listed in Supplemental Table 2. *N. benthamiana* leaves were co-infiltrated with *Agrobacterium tumefaciens* (*A. tumefaciens*) GV3101 containing effector and reporter constructs and cultured for 2–3 days. LUC and REN activities were measured using the Dual-Luciferase Reporter Assay Kit (Promega, Madison, WI, USA) on a microplate reader (Tecan, Männedorf, Switzerland) following the manufacturer's instructions. LUC images were captured using an *in vivo* Plant Imaging System (Berthold, Stuttgart, Germany) with IndiGO software. Three biological replicates were used for each assay.

Y1H assays

The *AcBAM3.3* promoter was cloned into the pAbAi vector linearized with *Bbs*I and then transformed into the Y1H Gold yeast strain using a yeast transformation kit (Coolaber, SK2400, China). pGADT7 and pGADT7-AcCTS1 were then transformed into pAbAi-AcBAM3.3 promoter strains. Yeast cells were incubated on synthetic defined medium lacking Leu (SD/–Leu) with 0, 100, and 200 ng ml^{-1} AbA for 3–5 days at 30°C. Both positive (pGADT7-p53 + p53-AbAi) and negative controls were processed in parallel. The primers used in these experiments are listed in Supplemental Table 2.

EMSAs

The full-length CDS of *AcCTS1* was inserted into the pMal-C6T vector by double digestion with *Not*I and *Bam*HI. The primers used are listed in Supplemental Table 2. The construct was transformed into *E. coli* strain BL21 (DE3). The recombinant His-MBP-AcCTS1 protein was induced with 0.5 mM isopropyl β-D-thiogalactoside (IPTG) for 18 h at 16°C and purified using nickel-nitrilotriacetic acid agarose (Smart-Lifesciences, Changzhou, China) according to the manufacturer's instructions. Probes containing DRE *cis*-acting elements, labeled and unlabeled with 6-FAM at their 5′ ends, as well as mutated elements, were synthesized by Sangon Biotech (Shanghai, China). The 6-FAM-labeled probes were incubated with recombinant His-MBP-AcCTS1 in binding buffer (50 μg ml^{-1} BSA, 1 mM benzamidine, 10 ng μl^{-1} Poly (deoxyinosinic-deoxycytidylic acid), 0.5 mM PMSF, and 0.5 mM DTT) for 40 min at RT. The free and bound probes were then separated by native acrylamide gel electrophoresis. Unlabeled probes were used as competitors, and His-MBP served as a negative control.

Transient analysis in *N. benthamiana* leaves and 'Jintang No. 3' fruit

Full-length CDSs of *AcBAM3.1/3.2/3.3/3.5* were individually cloned into the pK7GW35 vector to generate OE constructs and then transformed into *A. tumefaciens* strain GV3101. An injection mix was prepared at a ratio of OE construct to p19 of 0.8:0.1. OE constructs were injected into the dorsal end of the leaf, separated by the main vein, and a control mix of EV and p19 was injected into the opposite end. After infiltration (2–3 days), the injected leaves were carefully removed and placed in the dark at ∼25°C to hydrate overnight. Leaves were immersed in boiling water for 30 s and then decolorized in 25 ml of 95% ethanol for 2–5 h. Afterward, 0.5 ml of iodine/potassium iodide solution (5 g iodine and 10 g potassium iodide dissolved in 10 ml water) was added and left to stain for 2–5 h. After staining, the leaves were rinsed with water 3 times, then soaked in water for 24 h. Photographic analysis was performed to quantify starch content.

Transient OE in kiwifruit was performed as described by Zhang et al. (2018). Full-length CDSs of *AcCTS1* and *AcPUB11* were individually cloned into the pK7GW35 vector to construct OE vectors. CDS fragments (300 bp) downstream of the *AcCTS1* and *AcPUB11* start codons (ATG) were inserted into the pHELLSGATE 8 vector to generate transient silencing vectors (RNAi). All constructs were electroporated into *A. tumefaciens* strain GV3101. The injection mix was prepared at a ratio of OE/RNAi to p19 of 0.8:0.1, with red ink added at 1/50 of the total volume. The mixture was injected into fruit, with the same proportion of EV, p19, and blue ink used as a control. Mature JT fruits (120 days after flowering) were harvested at 8:00 am, and the wound site was cured under ventilated conditions at 20°C for 5–10 h. The needle of a 1-ml syringe was carefully inserted along the longitudinal diameter, ∼3 cm into the outer pericarp of each fruit, and the syringe was emptied. Each fruit was injected at 4 sites (two with OE/RNAi and two with EV mix), with 300 μl injected per site. Three biological replicates were performed, each using at least 12 fruits. Tissue infiltrated with ink was collected 2.5–3 days post injection, quickly frozen with liquid nitrogen, and stored at −80°C for further analysis.

Transgenic kiwifruit calli and tomato

AcBAM3.3, *AcBAM3.5*, and *AcCTS1* were each cloned into the pK7GW35S vector under the control of the CaMV 35S promoter. pK7GW35S also contains a red fluorescent protein marker driven by CaMV 35S. CRISPR–Cas9 genome-editing constructs of *AcCTS1* and *AcPUB11* were produced as described by Zhang et al. (2017). Mutations were detected using the primer pairs listed in Supplemental Table 2. The PCR products were submitted for genotyping analysis using a Hi-TOM sequencing platform (http://www.hi-tom.net/hi-tom/) to determine mutation efficiency. The constructs were introduced into *A. tumefaciens* strain EHA105 and stably transformed into HY as described previously (Souleyre et al., 2022). Murashige and Skoog medium was used as the base and supplemented with 1 mg ml^{-1} auxin (2,4-D), 30 g l^{-1} sucrose, and 5.5 g l^{-1} phytagel, with the pH adjusted to 5.8. Cultures were incubated at 22°C ± 2°C in the dark for over 30 days. Calli with red fluorescence were observed using a handheld fluorescent light source (LUYOR-3415RG, USA), then collected, frozen in liquid nitrogen, and stored at −80°C for further analysis.

A. tumefaciens GV3101–mediated transformation of 'Micro-Tom' tomato (*Solanum lycopersicum* L.) was performed as described previously (Wang et al., 2005).

Screening of the Y2H cDNA library

Total RNA was extracted from the outer pericarp of kiwifruit after 8 days of temperature treatment and used to construct a Y2H cDNA library by

Oebiotech Biomedical Science and Technology (Shanghai, China). The full-length *AcCTS1* CDS was cloned into the pGBKT7 vector after double digestion with *Eco*RI and *Bam*HI. The construct was transformed into the Y2H Gold yeast strain using a yeast transformation kit (Coolaber, SK2400, China) to obtain bait. Bait and yeast library working solutions were co-incubated in 2× yeast peptone dextrose adenine medium at 30°C with shaking at 50 rpm for 20–24 h. The mixture was centrifuged at 3000 rpm for 5 min, and the supernatant was discarded. The pellet was then re-suspended in 10 ml of 0.9% NaCl and cultured on SD/−Leu/−Trp/−Ade/−His medium at 30°C for 3–5 days. Single colonies were selected and identified by DNA sequencing (Tsingke Biotech, Wuhan, China). The CDS of the candidate protein *AcPUB11* was cloned into the *Eco*RI and *Xho*I sites of the pGADT7 vector to construct a prey vector. Both bait and prey were co-transformed into the Y2H Gold yeast strain as described for the Y1H assays. After selection on SD/−Trp/−Leu medium, positive clones were suspended in 0.9% NaCl at optical density 600 = 0.2 and incubated on SD/−His/−Leu/−Trp/−Ade + X-α-gal medium for 3–5 days at 30°C.

Pull-down, coIP, BiFC, and LCI assays

The full-length CDS of *AcPUB11* was inserted into the pGEX-4T-1 vector by double digestion with *Eco*RI and *Bam*HI to produce the GST-AcPUB11 fusion protein. The construct was transformed into *E. coli* strain BL21 (DE3). Production of the GST-AcPUB11 fusion protein was induced with 0.5 mM IPTG for 18 h at 16°C, followed by purification using glutathione–Sepharose resin (Solarbio) according to the manufacturer's protocol. GST-AcPUB11 and MBP-AcCTS1 were combined in equal proportions. Pull-down buffer (0.5 ml of 20 mM [pH 8.0] Tris–HCl, 100 mM NaCl, 0.5 mM EDTA, and 0.5% Nonidet-40) was added, and MBP was used as a control. Glutathione–Sepharose resin (50 μl, Solarbio) was supplied, followed by incubation with rotation for 2 h at 4°C. The glutathione–Sepharose resin was washed 5 times with 1× PBS buffer (137 mM NaCl, 2.7 mM KCl, 10 mM Na_2HPO_4, and 2 mM KH_2PO_4 [pH 7.4]). The bound proteins were analyzed by immunoblot analysis with anti-GFP (ABclonal, China) or anti-HA (ABclonal) antibody and a goat anti-mouse immunoglobulin G (IgG) secondary antibody (ABclonal).

CoIP assays were performed as described by Qi et al. (2024). The full-length CDSs of *AcCTS1* and *AcPUB11* were inserted into the ph7lic vector after *Stu*I digestion to obtain HA-AcCTS1 and GFP-AcPUB11 fusion proteins. HA-AcCTS1 and GFP-AcPUB11 were transiently expressed in *N. benthamiana* leaves via *A. tumefaciens* GV3101. GFP-GUS was used as a control. After 2–3 days of culture, leaves were quick-frozen in liquid nitrogen and ground into powder, and total protein was extracted using radio immunoprecipitation assay lysis buffer (high) (Solarbio) according to the manufacturer's instructions. Total protein was incubated with anti-GFP magnetic beads (AlpalifeBio, Shenzhen, China) to immunoprecipitate either GFP-AcPUB11 or GFP-GUS. The immunoprecipitate was analyzed by immunoblotting with an anti-HA antibody (ABclonal).

The full-length CDS of *AcCTS1* was inserted into the pMDC43-nYFP vector, and the full-length CDS of *AcPUB11* was inserted into the pMDC43-cYFP vector. A segment of the polymorphic fragment sequence (V097) was cloned into both pMDC43-nYFP and pMDC43-cYFP as negative controls, and mCherry was used as a nuclear localization marker. *N. benthamiana* leaves were co-infiltrated with *A. tumefaciens* GV3101 carrying these constructs. After infiltration (2–3 days), YFP and mCherry fluorescence signals were observed using confocal microscopy (Leica Microsystems, Wetzlar, Germany) with the YFP filter (excitation/band pass: 514 nm; 520–551 nm) and the mCherry filter (excitation/band pass: 552 nm; 590–630 nm).

For LCI assays, the full-length CDS of *AcCTS1* was inserted into the JW-771-nLUC vector, and the full-length CDS of *AcPUB11* was inserted into the JW-772-cLUC vector. *N. benthamiana* leaves were co-infiltrated with *A. tumefaciens* GV3101 carrying constructs and EVs. After infiltration (2–3 days), LUC images were captured as described for the dual-luciferase assays.

Ubiquitination assays *in vitro* and *in vivo*

The full-length CDS of *AcCTS1* was cloned into pCDFDuet at the *Eco*RI and *Bam*HI restriction sites. The full-length CDS of *AcPUB11* was inserted into pACYCDuet at the *Eco*RI and *Stu*I restriction sites. The ubiquitination assay was performed in bacteria using a reconstituted system as described previously by Han et al. (2017). In brief, the two resulting plasmids and a pET-28a-FLAG-UBQ plasmid were co-transformed into *E. coli* BL21 (DE3). A positive strain containing the 3 plasmids was cultured at 37°C. After the optical density at 600 nm reached 0.6, recombinant proteins were induced with 0.5 mM IPTG for 12 h at 28°C, followed by overnight storage at 4°C. Bacteria (300 μl) were harvested and centrifuged at 12 000 *g* for 5 min. The bacterial pellets were analyzed by immunoblotting using anti-HA, anti-Myc, or anti-FLAG antibodies (ABclonal), followed by a goat anti-mouse IgG secondary antibody (ABclonal).

For ubiquitination assays *in vivo*, the FLAG-Ub sequence was amplified from the pET-28a-FLAG-UBQ vector and inserted into the pK7lic1.0 vector at the *Sma*I restriction site. *N. benthamiana* leaves were co-infiltrated with *A. tumefaciens* GV3101 containing HA-AcCTS1, GFP-AcPUB11, and FLAG-Ub. GFP-GUS was used as a negative control. After infiltration for 2–3 days, total protein was extracted as described for the coIP assays and analyzed by immunoblotting using anti-HA or anti-Ub antibodies (ABclonal), followed by a goat anti-mouse IgG secondary antibody (ABclonal).

In vivo protein degradation assay

HA-AcCTS1 was co-expressed with AcPUB11 in *N. benthamiana* leaves. MG132 was added as another combination following *A. tumefaciens* strain GV3101–mediated infiltration. HA-AcCTS1, co-expressed with the GUS reporter gene, was used as a control. After 2–3 days, total proteins were isolated from transiently infiltrated *N. benthamiana* leaves, separated by SDS–PAGE as described for immunoblot analysis, and immunoblotted with an anti-HA antibody (ABclonal).

Statistical analysis

Data are shown as mean ± SE from at least three independent biological replicates. Statistical differences between samples were analyzed by Student's *t*-test (*$P < 0.05$, **$P < 0.01$, and ***$P < 0.001$). GraphPad Prism 8.0 and Microsoft Excel 2007 were used for data analyses. Nine-quadrant maps were created using R software (v.3.5.1).

DATA AND CODE AVAILABILITY

Sequence data for all kiwifruit genes referenced in this article are available in the Kiwifruit Genome Database under the following accession numbers: AcBAM3.1 (Acc15874), AcBAM3.2 (Acc28818), AcBAM3.5 (Acc28966), AcCTS1 (Acc12510), AcCTS2 (Acc20268), and AcPUB11 (Acc09233). Sequence data for AcBAM3.3 (PQ932602), *Arabidopsis* AtBAM3 (CAB58423), and soybean GmBMY1 (P10538) are available in the NCBI database.

FUNDING

This work was supported by the National Key Research and Development Program of China (2021YFD1200202-08), the National Natural Science Foundation of China (32272779 and 32573103), the China Postdoctoral Science Foundation (2024M751043), the Hubei Province Postdoctoral Innovative Talent Training Project (2024HBBHCXA046), the Hubei Key Research and Development Program (2023BBB064), the China Agriculture Research System (CARS-26), the Hubei Provincial International Science and Technology Cooperation Project (2025EHA056), the Open Project Program of the Jiangxi Provincial Key Laboratory of Plantation and High-Value Utilization of Specialty

Fruit Tree and Tea (GCSZ202401), and the Fundamental Research Funds for the Central Universities.

ACKNOWLEDGMENTS

We would like to express our gratitude to Prof. Pengwei Wang and his team members—Dr. Zhen Tian, Dr. Erlin Gao, and Dr. Ye Guo—at Huazhong Agricultural University for technical support with the ubiquitination, BiFC, and RNAi assays. We also acknowledge Prof. Zhiyong Pan (Huazhong Agricultural University) and Dr. Fuxi Bai (Hubei Academy of Agricultural Sciences) for generously providing the pK7GW35S plasmid. In addition, we appreciate the insightful suggestions offered by Dr. Simona Nardozza (New Zealand Bioeconomy Science Institute, formerly Plant & Food Research). No conflict of interest is declared.

AUTHOR CONTRIBUTIONS

Y.Z. and A.L. conceived the project and designed the research. A.L., Y.M., X.C., Z. Zhao, T.L., and G.D. performed the experiments. A.L., Z. Zeng, and Y.H. analyzed the data. A.L., R.G.A., and Y.Z. wrote and revised the manuscript. Y.C. and X.D. provided advice on the manuscript. All authors discussed the results and approved the final manuscript.

SUPPLEMENTAL INFORMATION

Supplemental information is available at *Plant Communications Online*.

Received: August 19, 2025
Revised: December 6, 2025
Accepted: January 19, 2026
Published: January 21, 2026

REFERENCES

An, J.P., Wang, X.F., Zhang, X.W., Xu, H.F., Bi, S.Q., You, C.X., and Hao, Y.J. (2020a). An apple MYB transcription factor regulates cold tolerance and anthocyanin accumulation and undergoes MIEL1-mediated degradation. Plant Biotechnol. J. **18**:337–353.

An, J., Wang, X., and Hao, Y. (2020b). BTB/TAZ protein MdBT2 integrates multiple hormonal and environmental signals to regulate anthocyanin biosynthesis in apple. J. Integr. Plant Biol. **62**:1643–1646.

Asiche, W.O., Mitalo, O.W., Kasahara, Y., Tosa, Y., Mworia, E.G., Ushijima, K., Nakano, R., and Kubo, Y. (2017). Effect of storage temperature on fruit ripening in three kiwifruit cultivars. Hort. J. **86**:403–410.

Asiche, W.O., Mitalo, O.W., Kasahara, Y., Tosa, Y., Mworia, E.G., Owino, W.O., Ushijima, K., Nakano, R., Yano, K., and Kubo, Y. (2018). Comparative transcriptome analysis reveals distinct ethylene–independent regulation of ripening in response to low temperature in kiwifruit. BMC Plant Biol. **18**:47.

Atkinson, R.G., Gunaseelan, K., Wang, M.Y., Luo, L., Wang, T., Norling, C.L., Johnston, S.L., Maddumage, R., Schröder, R., and Schaffer, R.J. (2011). Dissecting the role of climacteric ethylene in kiwifruit (*Actinidia chinensis*) ripening using a 1-aminocyclopropane-1-carboxylic acid oxidase knockdown line. J. Exp. Bot. **62**:3821–3835.

Burdon, J., Pidakala, P., Martin, P., and Billing, D. (2017). Softening of 'Hayward' kiwifruit on the vine and in storage: The effects of temperature. Sci. Hortic. **220**:176–182.

Centeno, D.C., Osorio, S., Nunes-Nesi, A., Bertolo, A.L.F., Carneiro, R.T., Araújo, W.L., Steinhauser, M.C., Michalska, J., Rohrmann, J., Geigenberger, P., et al. (2011). Malate plays a crucial role in starch metabolism, ripening, and soluble solid content of tomato fruit and affects postharvest softening. Plant Cell **23**:162–184.

Chen, X., He, B., Zhang, W., et al. (2025). Fluctuating temperature and 1-methylcyclopropene (1-MCP) treatment for controlling the ripening and supply of ready-to-eat kiwifruit. Food Qual. Saf. **10**:fyaf073.

Coego, A., Brizuela, E., Castillejo, P., Ruíz, S., Koncz, C., del Pozo, J.C., Piñeiro, M., Jarillo, J.A., Paz-Ares, J., and León, J.; TRANSPLANTA Consortium (2014). The TRANSPLANTA collection of *Arabidopsis* lines: a resource for functional analysis of transcription factors based on their conditional overexpression. Plant J. **77**:944–953.

Cui, Q., Li, X., Hu, S., Yang, D., Abozeid, A., Yang, Z., Jiang, J., Ren, Z., Li, D., Li, D., et al. (2024). The critical role of phenylpropanoid biosynthesis pathway in Lily resistance against gray mold. Int. J. Mol. Sci. **25**:11068.

Dale, M.F.B., and Bradshaw, J.E. (2003). Progress in improving processing attributes in potato. Trends Plant Sci. **8**:310–312.

Dang, Q., Sha, H., Nie, J., Wang, Y., Yuan, Y., and Jia, D. (2021). An apple (*Malus domestica*) AP2/ERF transcription factor modulates carotenoid accumulation. Hortic. Res. **8**:223.

Deng, H., Chen, Y., Liu, Z., Liu, Z., Shu, P., Wang, R., Hao, Y., Su, D., Pirrello, J., Liu, Y., et al. (2022). *SlERF.F12* modulates the transition to ripening in tomato fruit by recruiting the co-repressor TOPLESS and histone deacetylases to repress key ripening genes. Plant Cell **34**:1250–1272.

Fan, Z.Q., Ba, L.J., Shan, W., Xiao, Y.Y., Lu, W.J., Kuang, J.F., and Chen, J.Y. (2018). A banana R2R3-MYB transcription factor *MaMYB3* is involved in fruit ripening through modulation of starch degradation by repressing starch degradation-related genes and *MabHLH6*. Plant J. **96**:1191–1205.

Gunaseelan, K., McAtee, P.A., Nardozza, S., Pidakala, P., Wang, R., David, K., Burdon, J., and Schaffer, R.J. (2019). Copy number variants in kiwifruit ETHYLENE RESPONSE FACTOR/APETALA2 (ERF/AP2)-like genes show divergence in fruit ripening associated cold and ethylene responses in C-REPEAT/DRE BINDING FACTOR-like genes. PLoS One **14**:e0216120.

Han, Y.C., Kuang, J.F., Chen, J.Y., Liu, X.C., Xiao, Y.Y., Fu, C.C., Wang, J.N., Wu, K.Q., and Lu, W.J. (2016). Banana transcription factor *MaERF11* recruits histone deacetylase MaHDA1 and represses the expression of *MaACO1* and expansins during fruit ripening. Plant Physiol. **171**:1070–1084.

Han, Y., Sun, J., Yang, J., Tan, Z., Luo, J., and Lu, D. (2017). Reconstitution of the plant ubiquitination cascade in bacteria using a synthetic biology approach. Plant J. **91**:766–776.

Hu, Q., Zhang, H., Song, Y., Song, L., Zhu, L., Kuang, H., and Larkin, R.M. (2024). REDUCED CHLOROPLAST COVERAGE proteins are required for plastid proliferation and carotenoid accumulation in tomato. Plant Physiol. **196**:511–534.

Jabbar, A., and East, A.R. (2016). Quantifying the ethylene induced softening and low temperature breakdown of 'Hayward' kiwifruit in storage. Postharvest Biol. Technol. **113**:87–94.

Jiang, G., Zhang, D., Li, Z., Liang, H., Deng, R., Su, X., Jiang, Y., and Duan, X. (2021). Alternative splicing of *MaMYB16L* regulates starch degradation in banana fruit during ripening. J. Integr. Plant Biol. **63**:1341–1352.

Jiang, M., Yang, Y., Wei, W., Wu, C., Shan, W., Kuang, J., Chen, J., Wei, S., and Lu, W. (2025). Interaction of MaERF11 with the E3 ubiquitin ligase MaRFA1 is involved in the regulation of banana starch degradation during postharvest ripening. Hortic. Plant J. **11**:608–618.

Li, A., Chen, J., Lin, Q., Zhao, Y., Duan, Y., Wai, S.C., Song, C., and Bi, J. (2021). Transcription factor *MdWRKY32* participates in starch–sugar metabolism by binding to the *MdBam5* promoter in apples during postharvest storage. J. Agric. Food Chem. **69**:14906–14914.

Li, D., and Zhu, F. (2018). Starch structure in developing kiwifruit. Int. J. Biol. Macromol. **120**:1306–1314.

Li, J., Dai, X., Li, Q., Jiang, F., Xu, X., Guo, T., and Zhang, H. (2023). Low temperatures inhibit the pectin degradation of 'Docteur Jules Guyot' pear (*Pyrus communis* L.). Int. J. Biol. Macromol. **242**:124719.

Li, T., Jiang, Z., Zhang, L., Tan, D., Wei, Y., Yuan, H., Li, T., and Wang, A. (2016). Apple (*Malus domestica) MdERF2* negatively affects

ethylene biosynthesis during fruit ripening by suppressing *MdACS1* transcription. Plant J. **88**:735–748.

Lim, S.L., Flütsch, S., Liu, J., Distefano, L., Santelia, D., and Lim, B.L. (2022). *Arabidopsis* guard cell chloroplasts import cytosolic ATP for starch turnover and stomatal opening. Nat. Commun. **13**:652.

Liu, J., Liu, M., Jia, C., Zhang, J., Miao, H., Wang, J., Zhang, J., Wang, Z., Xu, B., Li, X., and Jin, Z. (2021). Elucidating the mechanism of MaGWD1-mediated starch degradation cooperatively regulated by *MaMADS36* and *MaMADS55* in banana. Postharvest Biol. Technol. **179**:111587.

Miao, H., Sun, P., Liu, Q., Liu, J., Jia, C., Zhao, D., Xu, B., and Jin, Z. (2020). Molecular identification of the key starch branching enzyme-encoding gene *SBE2.3* and its interacting transcription factors in banana fruits. Hortic. Res. **7**:101.

Mitalo, O.W., Tokiwa, S., Kondo, Y., Otsuki, T., Galis, I., Suezawa, K., Kataoka, I., Doan, A.T., Nakano, R., Ushijima, K., and Kubo, Y. (2019). Low temperature storage stimulates fruit softening and sugar accumulation without ethylene and aroma volatile production in kiwifruit. Front. Plant Sci. **10**:888.

Mitalo, O.W., Otsuki, T., Okada, R., Obitsu, S., Masuda, K., Hojo, Y., Matsuura, T., Mori, I.C., Abe, D., Asiche, W.O., et al. (2020). Low temperature modulates natural peel degreening in lemon fruit independently of endogenous ethylene. J. Exp. Bot. **71**:4778–4796.

Mworia, E.G., Yoshikawa, T., Salikon, N., Oda, C., Asiche, W.O., Yokotani, N., Abe, D., Ushijima, K., Nakano, R., and Kubo, Y. (2012). Low-temperature-modulated fruit ripening is independent of ethylene in 'Sanuki Gold' kiwifruit. J. Exp. Bot. **63**:963–971.

Ngcobo, B.L., Bertling, I., and Clulow, A.D. (2022). Artificial daylength enhancement (pre-sunrise and post-sunset) with blue and red led lights affects tomato plant development, yield, and fruit nutritional quality. Hortic. Environ. Biotechnol. **63**:847–856.

Pék, Z., Helyes, L., and Lugasi, A. (2010). Color changes and antioxidant content of vine and postharvest-ripened tomato fruits. Hortscience **45**:466–468.

Pilkington, S.M., Crowhurst, R., Hilario, E., Nardozza, S., Fraser, L., Peng, Y., Gunaseelan, K., Simpson, R., Tahir, J., Deroles, S.C., et al. (2018). A manually annotated *Actinidia chinensis* var. *chinensis* (kiwifruit) genome highlights the challenges associated with draft genomes and gene prediction in plants. BMC Genom. **19**:257.

Qi, Y., Wu, J., Yang, Z., Li, H., Liu, L., Wang, H., Sun, X., Wu, X., Nie, J., Zhou, J., et al. (2024). Chloroplast elongation factors break the growth–immunity trade-off by simultaneously promoting yield and defence. Nat. Plants **10**:1576–1591.

Qiu, J., Cai, C., Shen, M., Gu, X., Zheng, L., Sun, L., Teng, Y., Zou, L., and Yu, H. (2024). Responses of growth, yield and fruit quality of strawberry to elevated CO2, LED supplemental light, and their combination in autumn through spring greenhouse production. Plant Growth Regul. **102**:351–365.

Sanchez-Ballesta, M.T., Alvarez, I., Escribano, M.I., Merodio, C., and Romero, I. (2020). Effect of high CO2 levels and low temperature on stilbene biosynthesis pathway gene expression and stilbenes production in white, red and black table grape cultivars during postharvest storage. Plant Physiol. Biochem. **151**:334–341.

Murakami, S., Ikoma, Y., and Yano, M. (2015). Fruit ripening process in red kiwifruit cultivar 'Rainbow Red' (*Actinidia chinensis*) on vines. Environ. Control Biol. **53**:159–163.

Shi, W., Ma, Q., Yin, W., Liu, T., Song, Y., Chen, Y., Song, L., Sun, H., Hu, S., Liu, T., et al. (2022). The transcription factor *StTINY3* enhances cold-induced sweetening resistance by coordinating starch resynthesis and sucrose hydrolysis in potato. J. Exp. Bot. **73**:4968–4980.

Shi, Y., Ding, Y., and Yang, S. (2018). Molecular regulation of CBF signaling in cold acclimation. Trends Plant Sci. **23**:623–637.

Song, Z., Chen, H., Zhao, Y., Wang, L., Chen, W., Li, X., and Zhu, X. (2025a). The regulatory MaIAA1-like-MaERF003 module modulates the ripening of 'Fenjiao' bananas. Plant Physiol. **199**:kiaf595.

Song, Z., Qin, J., Zheng, Q., Ding, X., Chen, W., Lu, W., Li, X., and Zhu, X. (2019). The involvement of the banana F-box protein MaEBF1 in regulating chilling-inhibited starch degradation through interaction with a MaNAC67-like protein. Biomolecules **9**:552.

Song, Z., Li, W., Chen, H., Shi, J., Zhu, H., Chen, W., Lu, W., Li, X., and Zhu, X. (2025b). E3 ligase SINAT 5 mediates ERF113 stability and negatively governs the module of ABI5-like-ERF113 in regulating banana fruit ripening. New Phytol. **248**:1284–1303.

Souleyre, E.J.F., Nieuwenhuizen, N.J., Wang, M.Y., Winz, R.A., Matich, A.J., Ileperuma, N.R., Tang, H., Baldwin, S.J., Wang, T., List, B.W., et al. (2022). Alcohol acyl transferase genes at a high-flavor intensity locus contribute to ester biosynthesis in kiwifruit. Plant Physiol. **190**:1100–1116.

Sun, S., Yu, J.P., Chen, F., Zhao, T.J., Fang, X.H., Li, Y.Q., and Sui, S.F. (2008). TINY, a dehydration-responsive element (DRE)-binding protein-like transcription factor connecting the DRE-and ethylene-responsive element-mediated signaling pathways in *Arabidopsis*. J. Biol. Chem. **283**:6261–6271.

Wang, H., Jones, B., Li, Z., Frasse, P., Delalande, C., Regad, F., Chaabouni, S., Latché, A., Pech, J.C., and Bouzayen, M. (2005). The tomato Aux/IAA transcription factor *IAA9* is involved in fruit development and leaf morphogenesis. Plant Cell **17**:2676–2692.

Wang, L., Zhao, M., Zhang, X., Zhao, T., Huang, C., Tang, Y., Li, Y., and Zhang, C. (2025). The ubiquitin ligase VviPUB19 negatively regulates grape cold tolerance by affecting the stability of ICEs and CBFs. Hortic. Res. **12**:uhae297.

Wei, W., Yang, Y.Y., Lakshmanan, P., Kuang, J.F., Lu, W.J., Pang, X.Q., Chen, J.Y., and Shan, W. (2023a). Proteasomal degradation of MaMYB60 mediated by the E3 ligase MaBAH1 causes high temperature-induced repression of chlorophyll catabolism and green ripening in banana. Plant Cell **35**:1408–1428.

Wei, W., Yang, Y.Y., Chen, J.Y., Lakshmanan, P., Kuang, J.F., Lu, W.J., and Shan, W. (2023b). *MaNAC029* modulates ethylene biosynthesis and fruit quality and undergoes MaXB3-mediated proteasomal degradation during banana ripening. J. Adv. Res. **53**:33–47.

Xiao, Y.y., Chen, J.y., Kuang, J.f., Shan, W., Xie, H., Jiang, Y.m., and Lu, W.j. (2013). Banana ethylene response factors are involved in fruit ripening through their interactions with ethylene biosynthesis genes. J. Exp. Bot. **64**:2499–2510.

Xiao, Y.Y., Kuang, J.F., Qi, X.N., Ye, Y.J., Wu, Z.X., Chen, J.Y., and Lu, W.J. (2018). A comprehensive investigation of starch degradation process and identification of a transcriptional activator *MabHLH6* during banana fruit ripening. Plant Biotechnol. J. **16**:151–164.

Xu, H., Zhu, L., Lin, Z., Wei, W., Yang, Y., Si, J., Shan, W., Chen, J., Lu, W., Kuang, J., and Wu, C. (2024). Banana *MabZIP21* positively regulates *MaBAM4, MaBAM7* and *MaAMY3* expression to mediate starch degradation during postharvest ripening. Postharvest Biol. Technol. **211**:112835.

Yin, X.R., Xie, X.L., Xia, X.J., Yu, J.Q., Ferguson, I.B., Giovannoni, J.J., and Chen, K.S. (2016). Involvement of an ethylene response factor in chlorophyll degradation during citrus fruit degreening. Plant J. **86**:403–412.

Zeng, Z., Li, Y., Zhu, M., Wang, X., Wang, Y., Li, A., Chen, X., Han, Q., Nieuwenhuizen, N.J., Ampomah-Dwamena, C., et al. (2024). Kiwifruit spatiotemporal multiomics networks uncover key tissue-

specific regulatory processes throughout the life cycle. Plant Physiol. **197**:kiae567.

Zhang, A.d., Wang, W.Q., Tong, Y., Li, M.J., Grierson, D., Ferguson, I., Chen, K.S., and Yin, X.R. (2018). Transcriptome analysis identifies a zinc finger protein regulating starch degradation in kiwifruit. Plant Physiol. **178**:850–863.

Zhang, F., LeBlanc, C., Irish, V.F., and Jacob, Y. (2017). Rapid and efficient CRISPR/Cas9 gene editing in Citrus using the YAO promoter. Plant Cell Rep. **36**:1883–1887.

Zhang, H., Liu, J., Hou, J., Yao, Y., Lin, Y., Ou, Y., Song, B., and Xie, C. (2014). The potato amylase inhibitor gene *SbAI* regulates cold-induced sweetening in potato tubers by modulating amylase activity. Plant Biotechnol. J. **12**:984–993.

Zhang, Y., Lai, X., Yang, S., Ren, H., Yuan, J., Jin, H., Shi, C., Lai, Z., and Xia, G. (2021). Functional analysis of tomato CHIP ubiquitin E3 ligase in heat tolerance. Sci. Rep. **11**:1713.

Zhao, M., Tian, R., Sun, X., and Zhang, W.H. (2023). lncRNA *MtCIR2* positively regulates plant-freezing tolerance by modulating CBF/DREB1 gene clusters. Plant Cell Environ. **46**:2450–2469.

Zhao, Y., Song, C., Brummell, D.A., Qi, S., Lin, Q., and Duan, Y. (2021). Jasmonic acid treatment alleviates chilling injury in peach fruit by promoting sugar and ethylene metabolism. Food Chem. **338**:128005.

Zhu, L.S., Shan, W., Wu, C.j., Wei, W., Xu, H., Lu, W.j., Chen, J.y., Su, X.g., and Kuang, J.f. (2021). Ethylene-induced banana starch degradation mediated by an ethylene signaling component *MaEIL2*. Postharvest Biol. Technol. **181**:111648.

Plant Communications, Volume *7*

Supplemental information

A cool temperature–induced ubiquitination-controlled transcription factor promotes starch degradation and ripening in kiwifruit

Ang Li, Yunhe Meng, Xiaoya Chen, Zhebin Zeng, Zhidan Zhao, Tiantian Li, Gang Ding, Ross G. Atkinson, Yue Huang, Yunjiang Cheng, Xiuxin Deng, and Yunliu Zeng

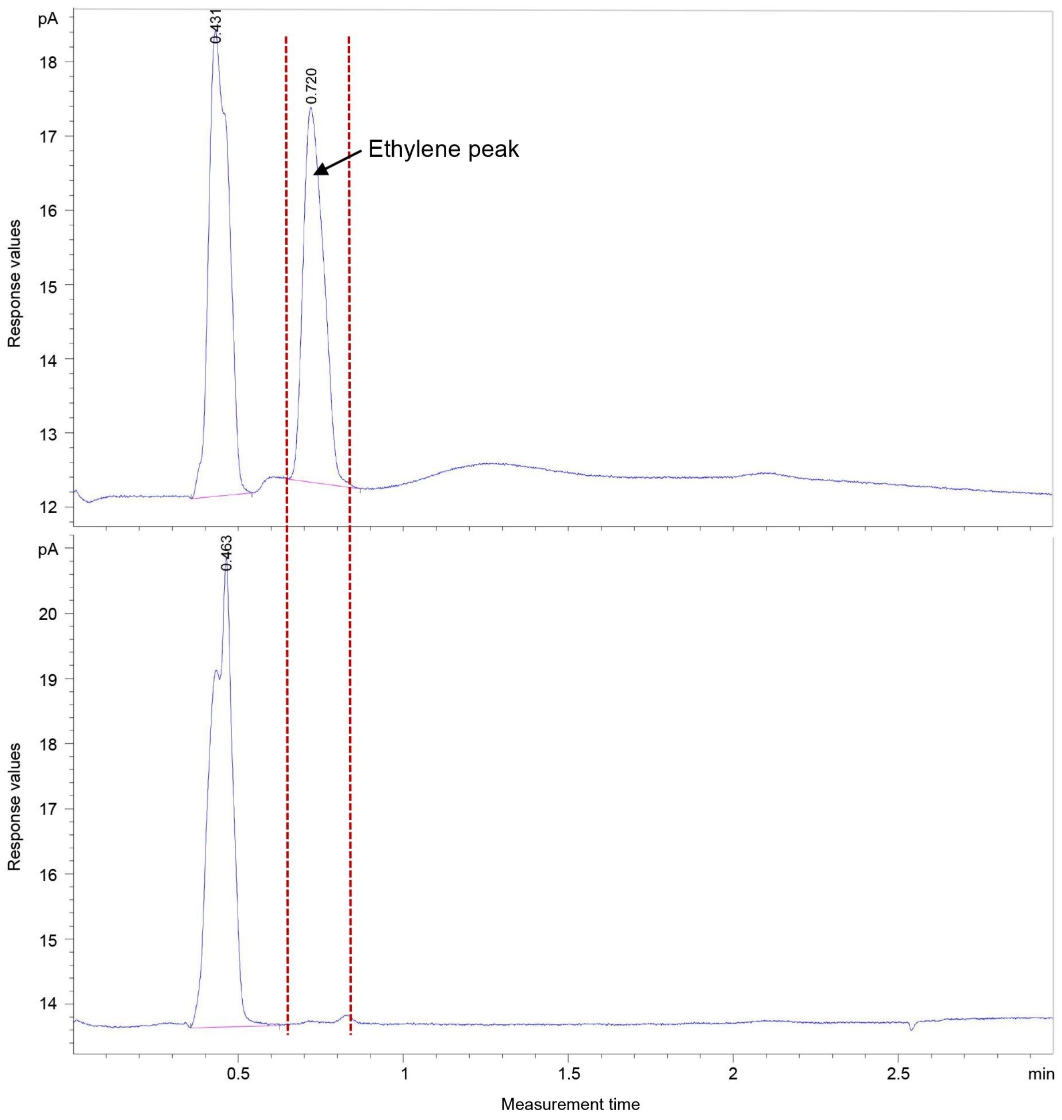

Supplementary Fig. 1 Ethylene analysis by gas chromatography in kiwifruit during storage. Representative chromatogram traces confirm a detectable ethylene peak in fruit treated with exogenous ethylene, but no ethylene peak in fruit stored at cool temperature (CT).

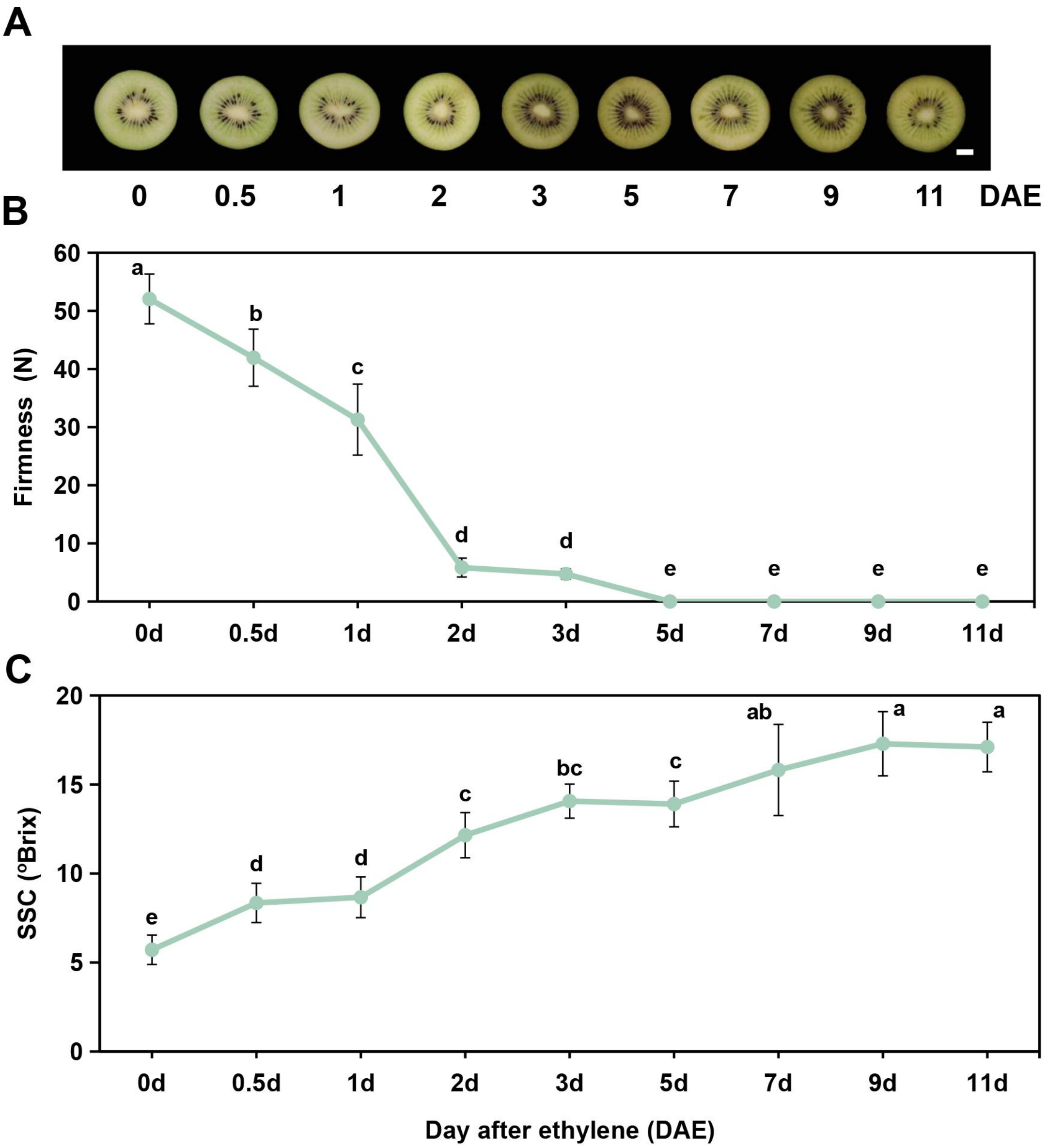

Supplementary Fig. 2 Changes in appearance, firmness and soluble solids content in 'Jintang No.3' kiwifruit treated with ethylene.

Alterations in appearance (A), firmness (B), and soluble solids content (C) of 'Jintang No.3' kiwifruit in 0–11 d following ethylene treatment (DAE). Scale bar represents 1 cm. Data are mean ± SE (n ≥ 3). Statistically significant differences (P<0.05) are denoted by lowercase letters.

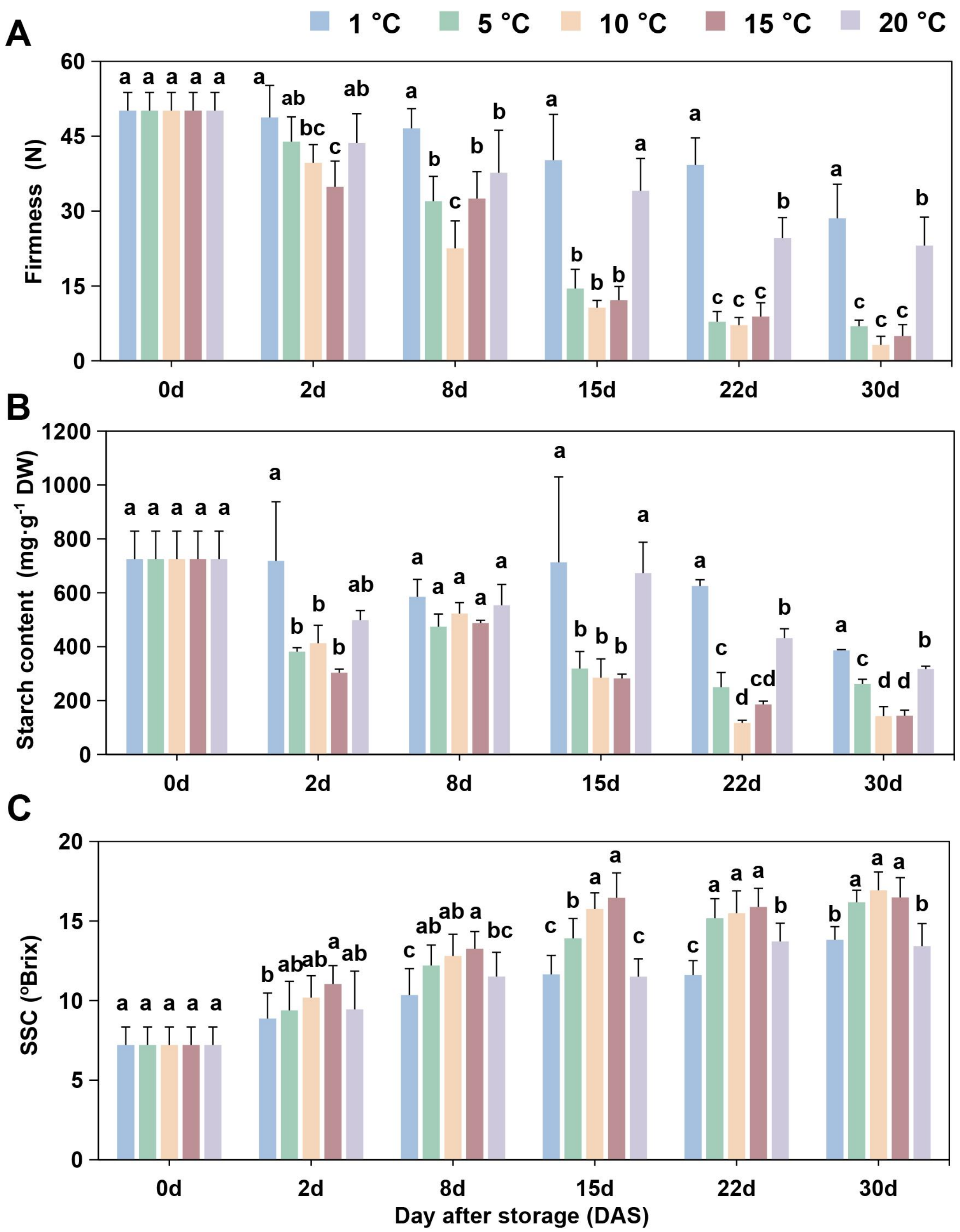

Supplementary Fig. 3 Changes in firmness, starch content and soluble solids content 'Hongyang' kiwifruit during storage at different temperatures.

Variations in firmness (A), starch content (B), and soluble solids content (C) of 'Hongyang' kiwifruit during storage at 1°C, 5°C, 10°C, 15°C, and 20°C. Different colors represent the respective storage temperatures. Data are mean ± SE (n ≥ 3). Statistically significant differences ($P<0.05$) are denoted by lowercase letters.

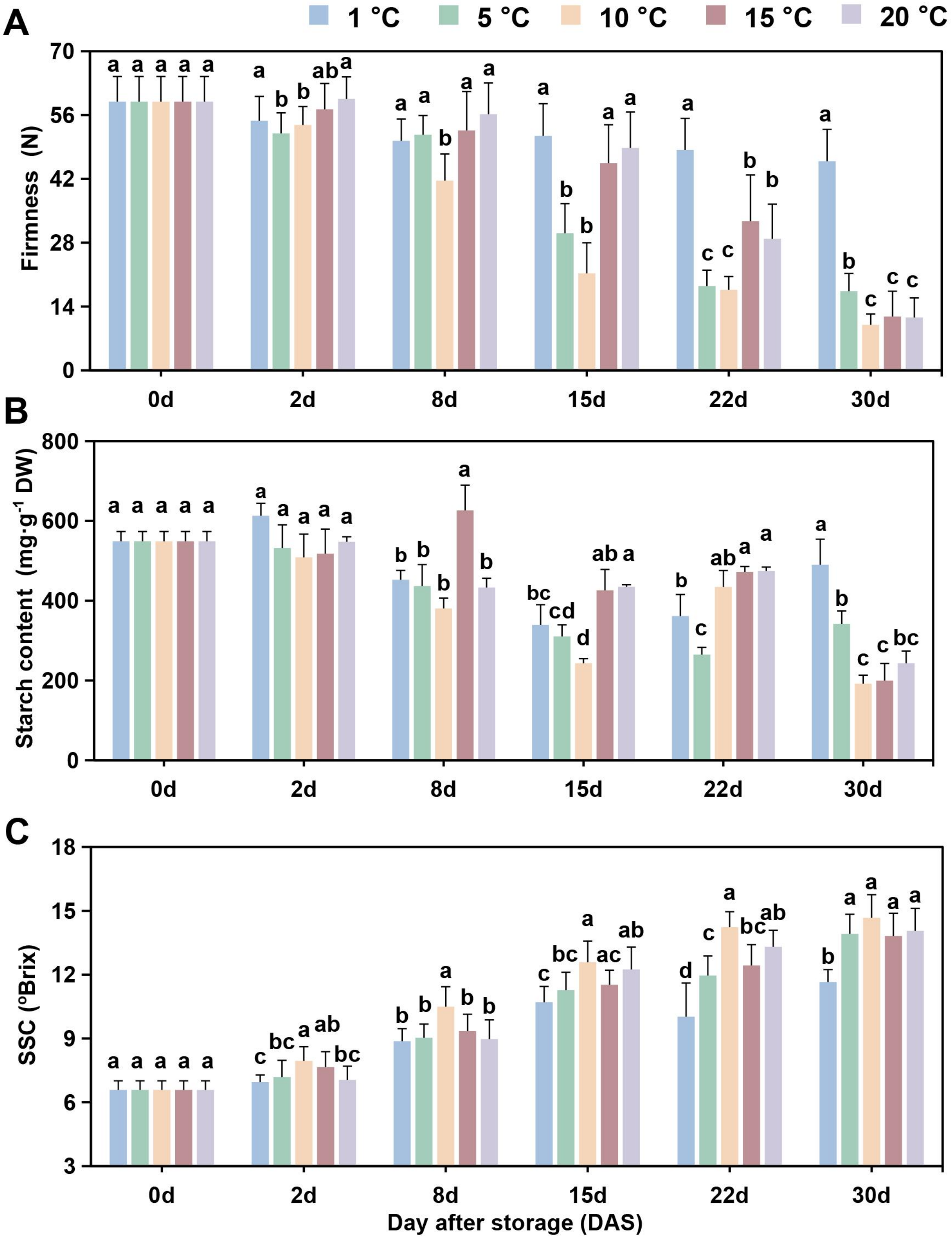

Supplementary Fig. 4 Changes in firmness, starch content and soluble solids content in 'Cuixiang' kiwifruit during storage at different temperatures.

Variations in starch content (A), soluble solids content (B), and firmness (C) of 'Cuixiang' kiwifruit during storage at 1°C, 5°C, 10°C, 15°C, and 20°C. Different colors represent the respective storage temperatures. Data are mean ± SE (n ≥ 3). Statistically significant differences (*P*<0.05) are denoted by lowercase letters.

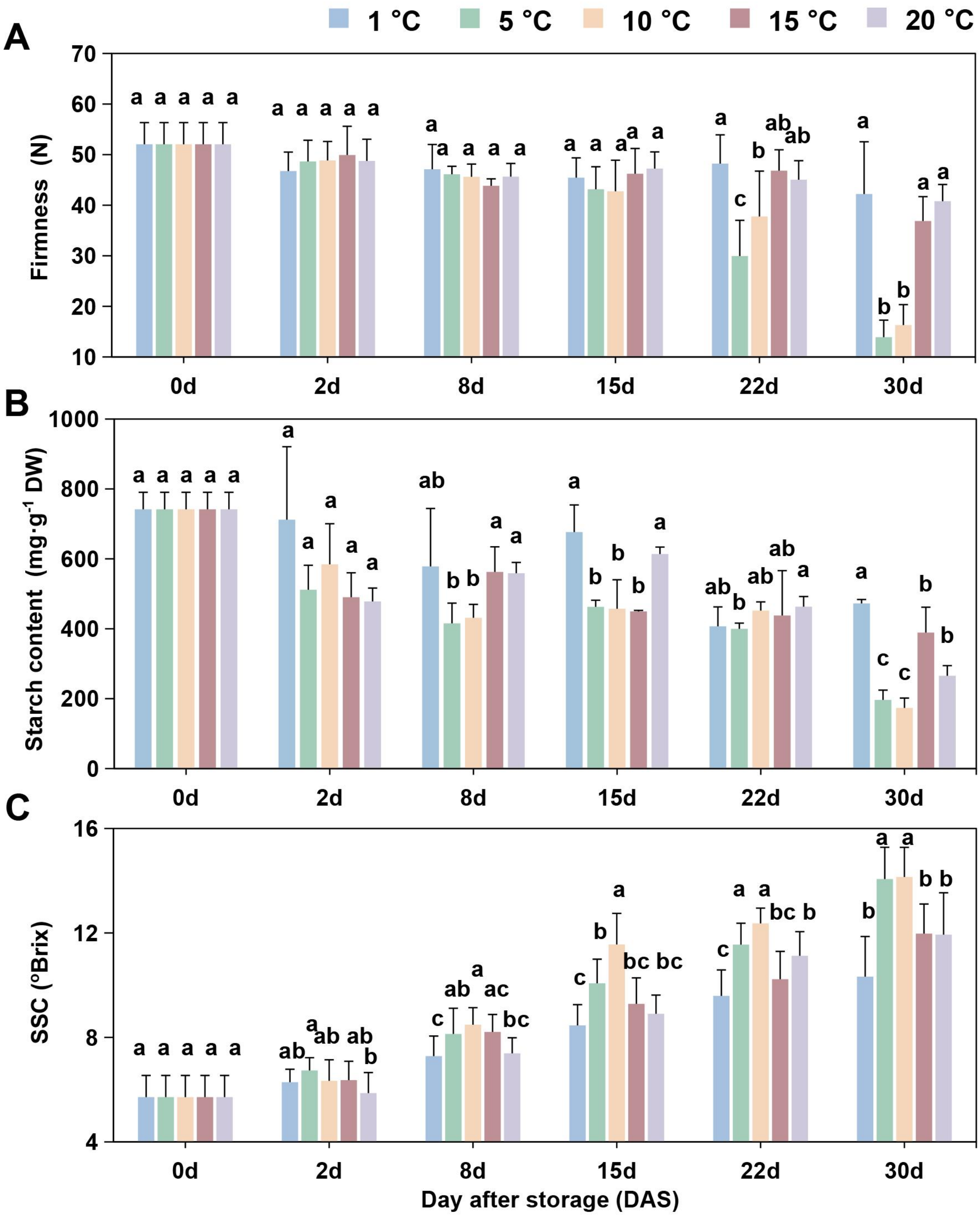

Supplementary Fig. 5 Changes in firmness, starch content, and soluble solids content in 'Jintang No.3' kiwifruit during storage at different temperatures. Changes in firmness (A), starch content (B), and soluble solids content (C) of 'Jintang No.3' kiwifruit during storage at 1°C, 5°C, 10°C, 15°C, and 20°C. Different colors represent the respective storage temperatures. Data are mean ± SE (n ≥ 3). Significant differences (*P*<0.05) are denoted by lowercase letters.

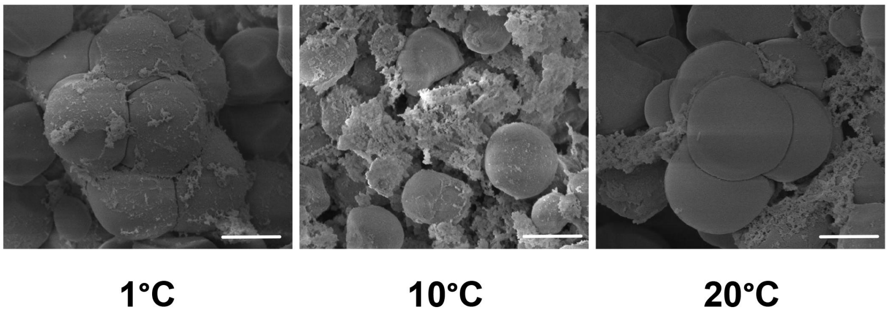

Supplementary Fig. 6 The ultrastructure of starch grain in kiwifruit under different temperature during storage

Starch grains were imaged by scanning electron microscopy at 22 day after storage (DAS) following storage at 1°C, 10°C, and 20°C in ‘Hongyang’ fruit. Scale = 5 μm.

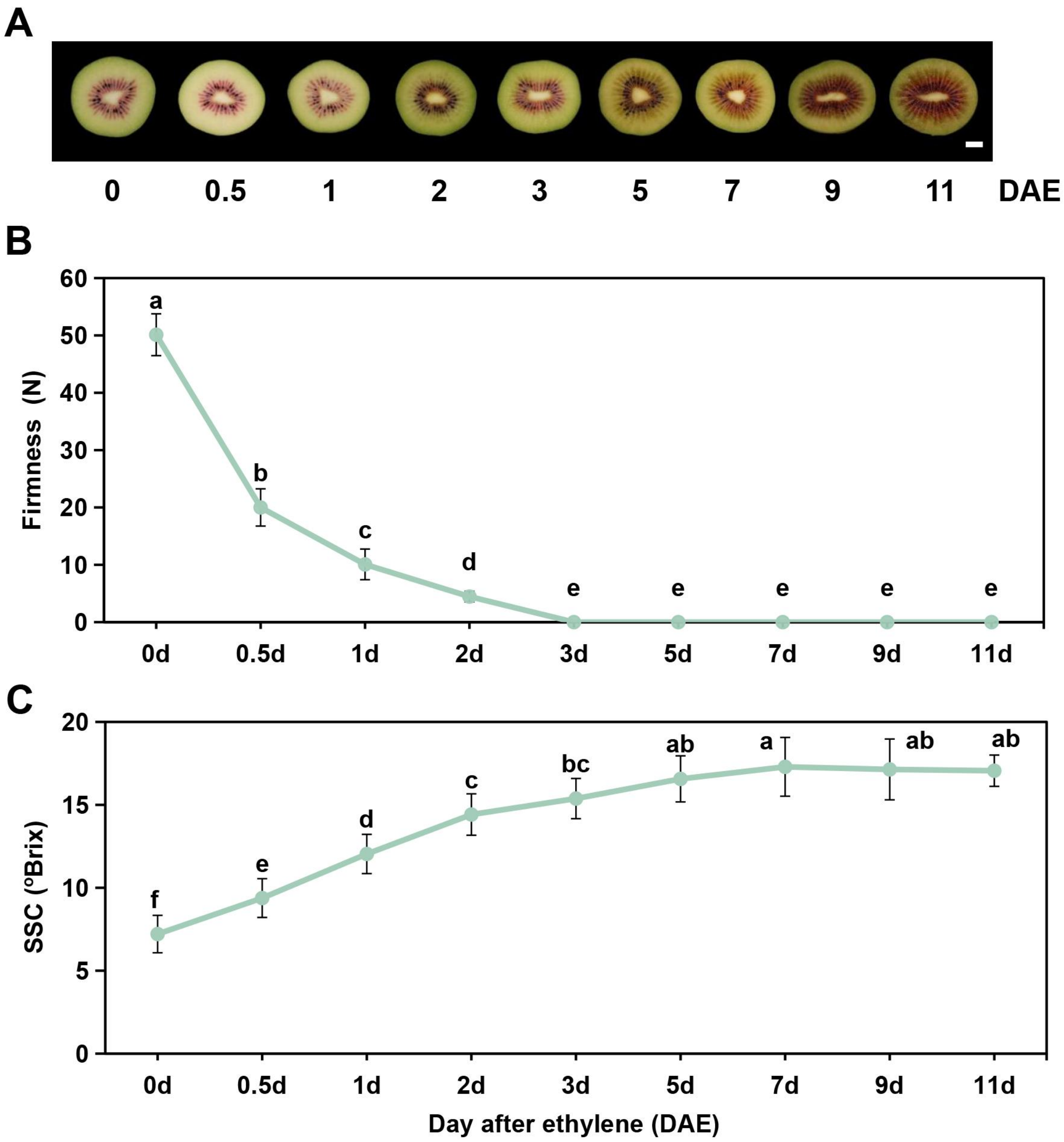

Supplementary Fig. 7 Changes in appearance, firmness and soluble solids content in 'Hongyang' kiwifruit treated with ethylene.

Changes in appearance (A), soluble solids content (B), and firmness (C) of 'Hongyang' kiwifruit in 0–11 d after ethylene treatment (DAE). Data are mean ± SE ($n \geq 3$). Scale bar represents 1 cm. Significant differences (P<0.05) are denoted by lowercase letters.

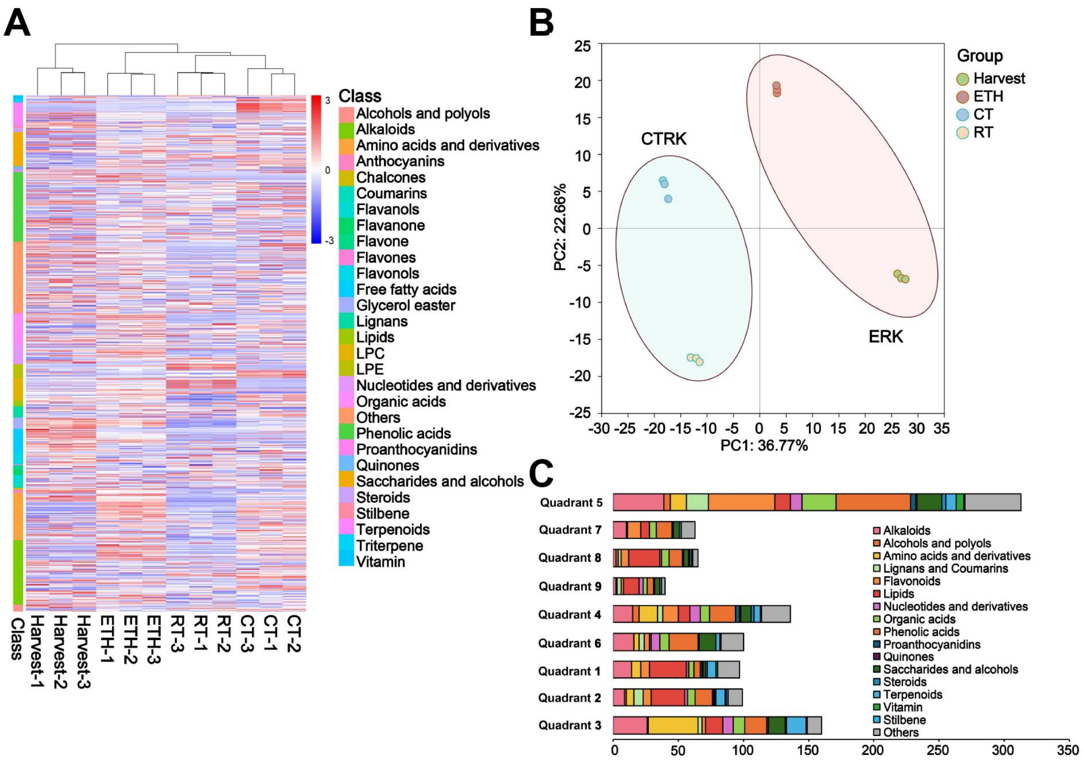

Supplementary Fig. 8 Metabolome analysis of cool-temperature and ethylene treated kiwifruits.

A) A summary of the metabolome data set for cool-temperature (CT) induced ripened kiwifruits (CTRKs) and ethylene-induced ripened kiwifruits (ERKs). Cluster analysis of metabolomes with Z-scores standardized to −3 to 3.

B) Principal component analysis (PCA) of metabolomes in cool-temperature induced ripened kiwifruits (CTRKs) and ethylene-induced ripened kiwifruits (ERKs).

C) Statistics of metabolite class categories in the nine quadrants, with color marks indicating various enrichments in metabolic pathways. A total of 1,073 metabolites were statistically analyzed, with a coefficient of variation >0.15 and average abundance >1. The nine quadrants (1−9) represent different accumulation patterns of metabolites under cool temperature and ethylene treatment, as shown in Fig. 1C. 1, increased at CT but decreased under ethylene; 2, increased at CT and stable under ethylene; 3, increased at both CT and ethylene; 4, stable at CT but decreased under ethylene; 5, unclassified; 6, stable at CT but increased under ethylene; 7, decreased at both CT and ethylene; 8, decreased at CT but stable under ethylene; 9, decreased at CT but increased under ethylene. The X-axis displays the number of metabolites in each quadrant, with 97, 99, 160, 136, 313, 100, 63, 65, and 40 metabolites in quadrants 1-9, respectively. Lipids were mainly enriched in quadrants 1, 2, 8, and 9, accounting for 28.9%, 26.3%, 37.0%, and 30.0%, respectively. Quadrant 3 was primarily enriched with amino acids and derivatives, making up 23.75%. Phenolic acids were predominantly found in quadrants 4, 5, 6, and 7, with respective proportions of 14.7%, 18.2%, 22.0%, and 19.1%.

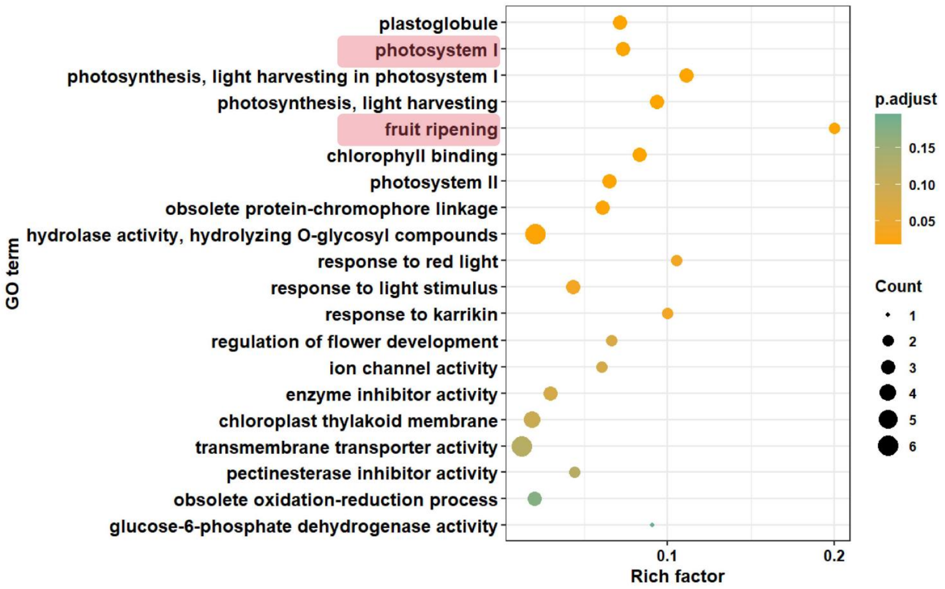

Supplementary Fig. 9 Gene ontology analysis.

Gene ontology (GO) analysis of genes specifically induced by cool temperature (CT) in 'Jingtang No.3' and 'Hongyang' kiwifruit. A total of 133 genes were analyzed. The X-axis shows the proportion of differentially expressed genes in each pathway relative to the total number of genes. The Y-axis represents the GO terms, which include molecular function, cellular component, and biological process. The color indicates the *P*-value, and the size of the dots reflects the number of differentially expressed genes in each category. Fruit ripening and photosynthesis, including processes like light harvesting in photosystem I and light harvesting in photosynthesis, displayed the highest enrichment, with factor values of 0.20 and 0.21, respectively.

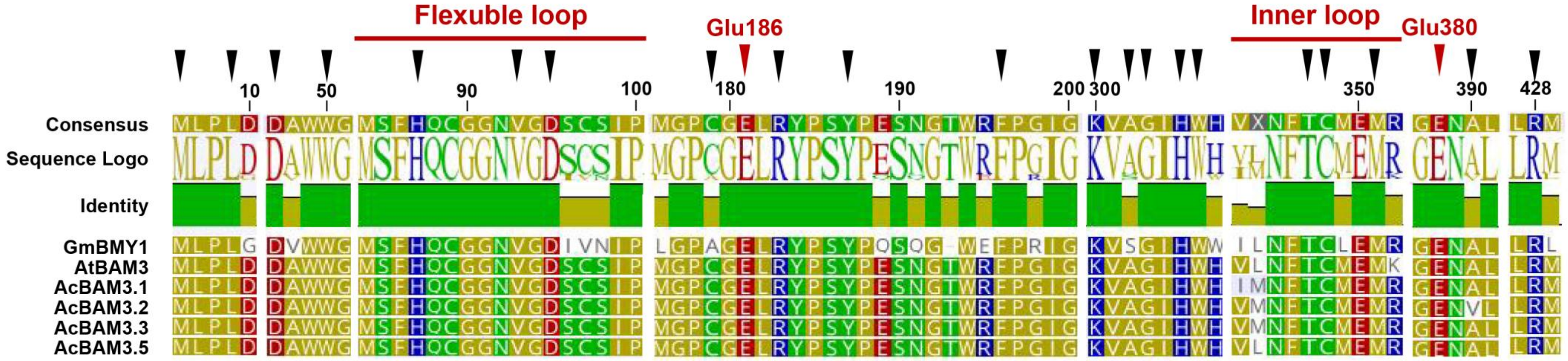

Supplementary Fig. 10 Alignment of core glucosyl hydrolase domains in β-amylase genes.
The alignment of AcBAM3.1, AcBAM3.2, AcBAM3.3, and AcBAM3.5 proteins, along with the Arabidopsis AtBAM3 protein and the soybean GmBMY1 protein was performed using the ClustalW sequence alignment program and analyzed with Geneious (R11 version). Color shading indicates conserved substitutions, while unshaded residues are not conserved. The bar graph illustrates sequence conservation, with green bars indicating high conservation and short brown bars indicating low conservation. Black arrowheads denote substrate binding residues, and red arrowheads indicate the catalytic residues Glu186 and Glu380. Red solid lines above the sequences mark the residues forming the flexible and inner loops

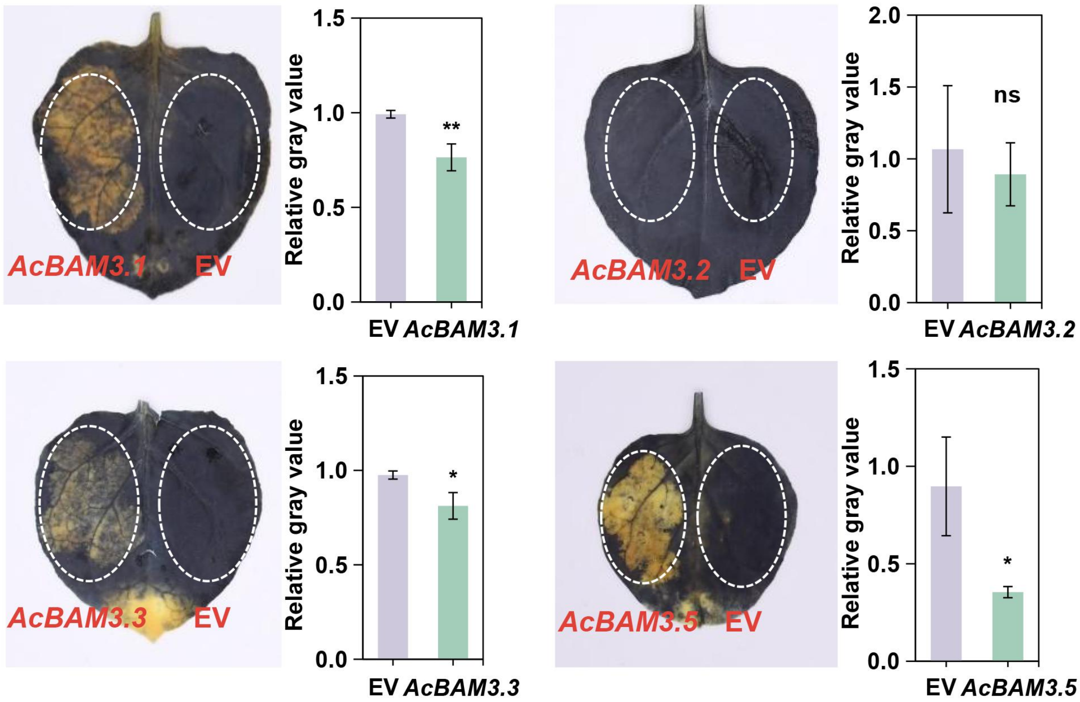

Supplementary Fig. 11 I-KI staining analysis of *N. benthamiana* leaves, with *AcBAM3* genes driven by the 35S promoter (left) and control infiltrated with empty vector (EV; right).

Starch degradation areas are circled with white dashed lines, and starch content is represented as grayscale value. ImageJ was used to calculate the grayscale value of the infiltrated area. Data are means ± SE (n = 3). Asterisks indicate significant differences between dehydrated samples and control at the same time point by Student's *t*-test (*, $P<0.05$; **, $P<0.01$); ns = not significant.

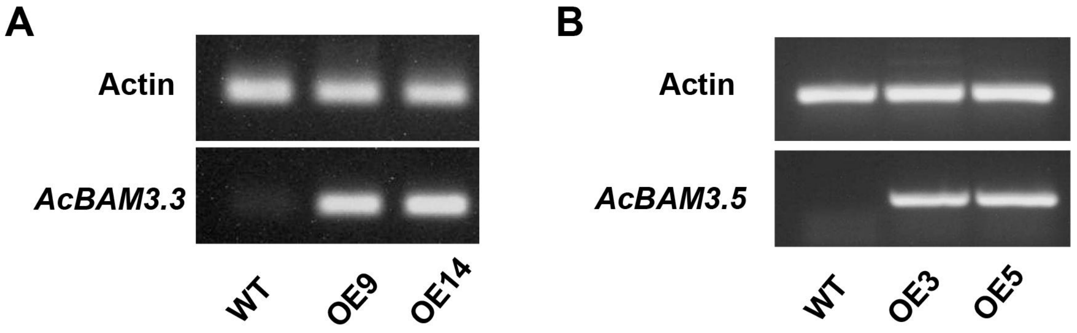

Supplementary Fig. 12 Semi-quantitative expression analysis of *AcBAM3.3*-OE (A) and *AcBAM3.5*-OE (B) tomato fruits. Primers were shown in Spplementary Table 2.

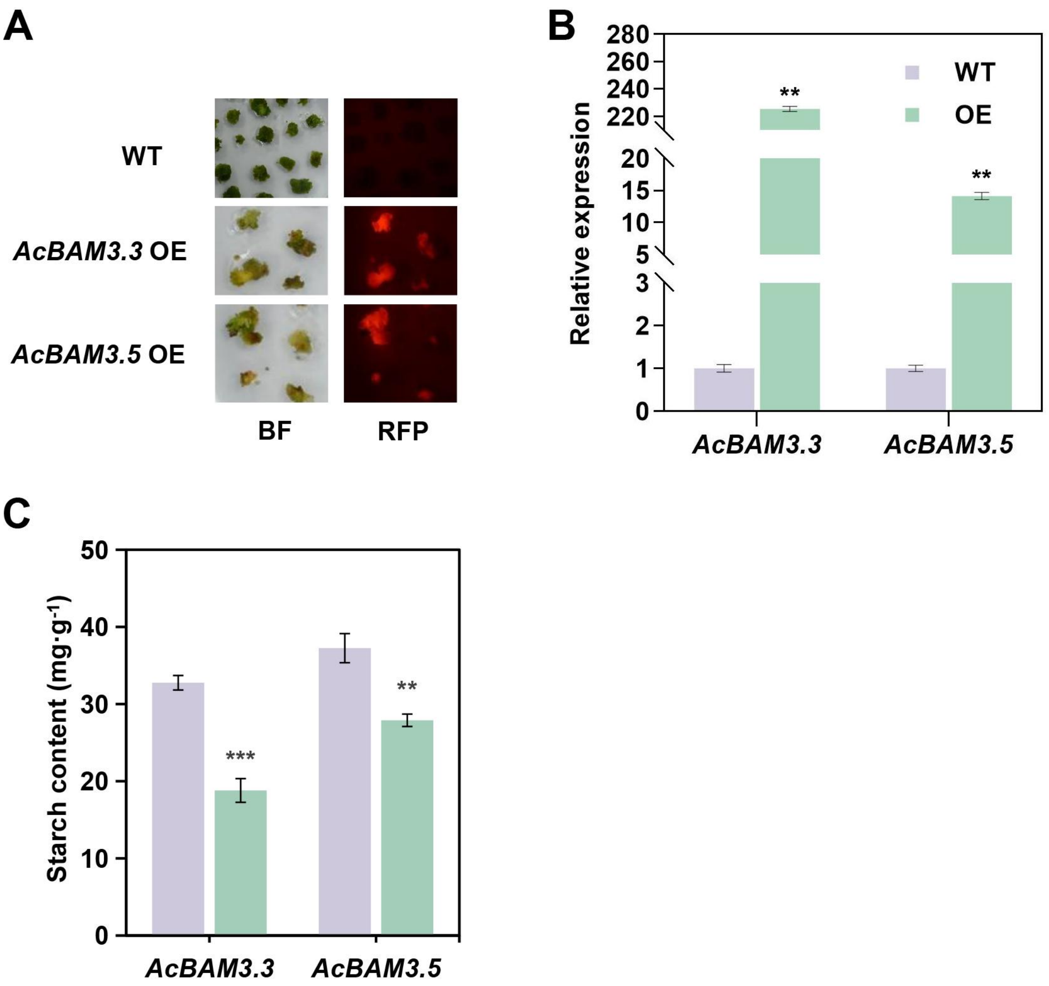

Supplementary Fig. 13 Analysis of starch degradation in kiwifruit callus overexpressing of *AcBAM3.3* and *AcBAM3.5*.

(A) Phenotypic appearance of wild-type (WT), *AcBAM3.3*-overexpressing (OE), and *AcBAM3.5*-OE callus in brightfield (BF) and red fluorescence light source (RFP) views.

(B) Expression analysis of *AcBAM3.3* and *AcBAM3.5* in WT, *AcBAM3.3*-OE, and *AcBAM3.5*-OE callus. Primers are shown in Supplementary Table 2.

(C) Starch content in WT, *AcBAM3.3*-OE, and *AcBAM3.5*-OE callus.

Data are mean ± SE of three replicates. Asterisks indicate significant differences as determined by Student's *t*-test (**, P<0.01; ***, P<0.001).

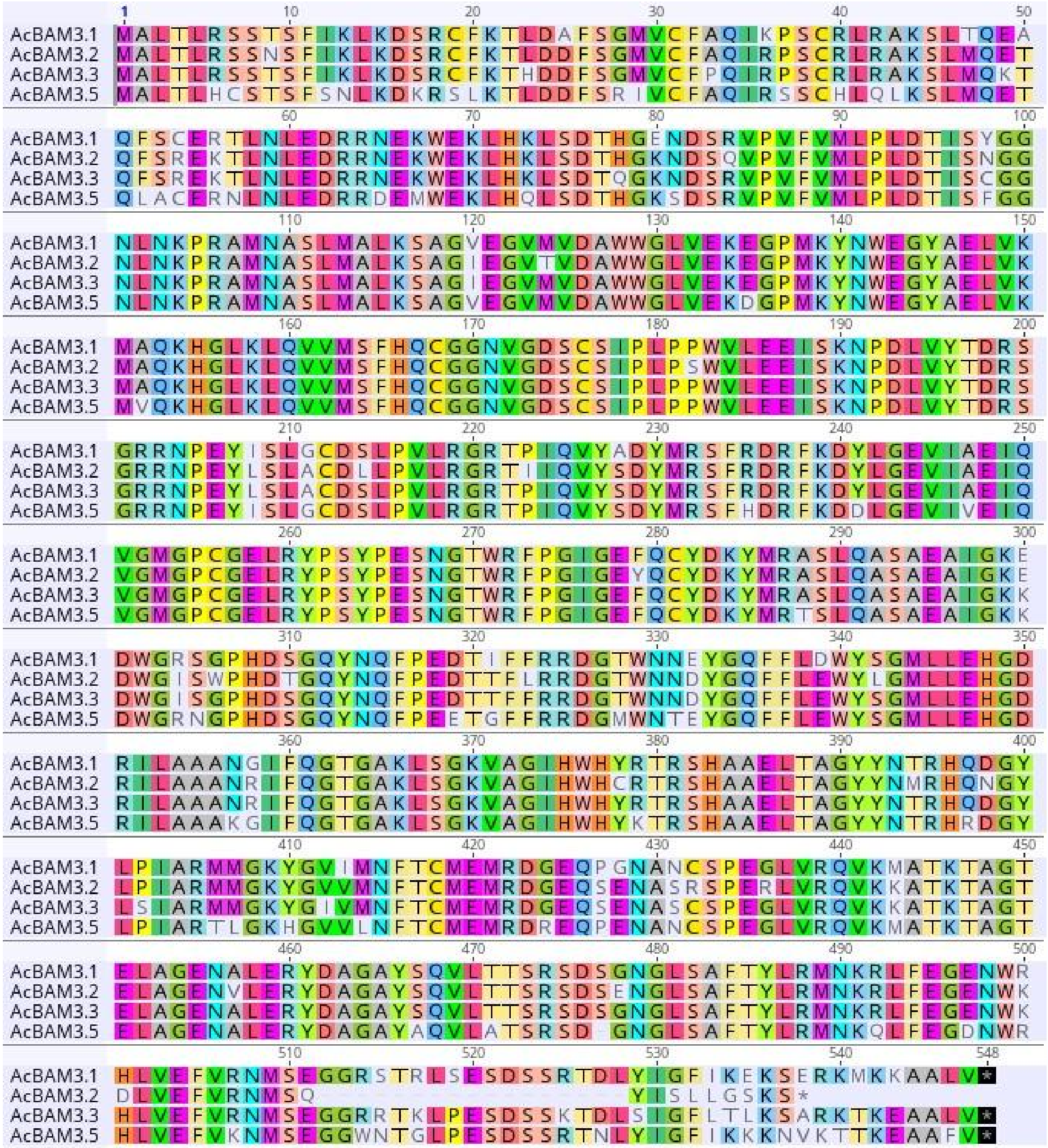

Supplementary Fig. 14 Amino acid alignment of AcBAM3s.
Amino acid sequences were aligned in Geneious (V2019.0.3).

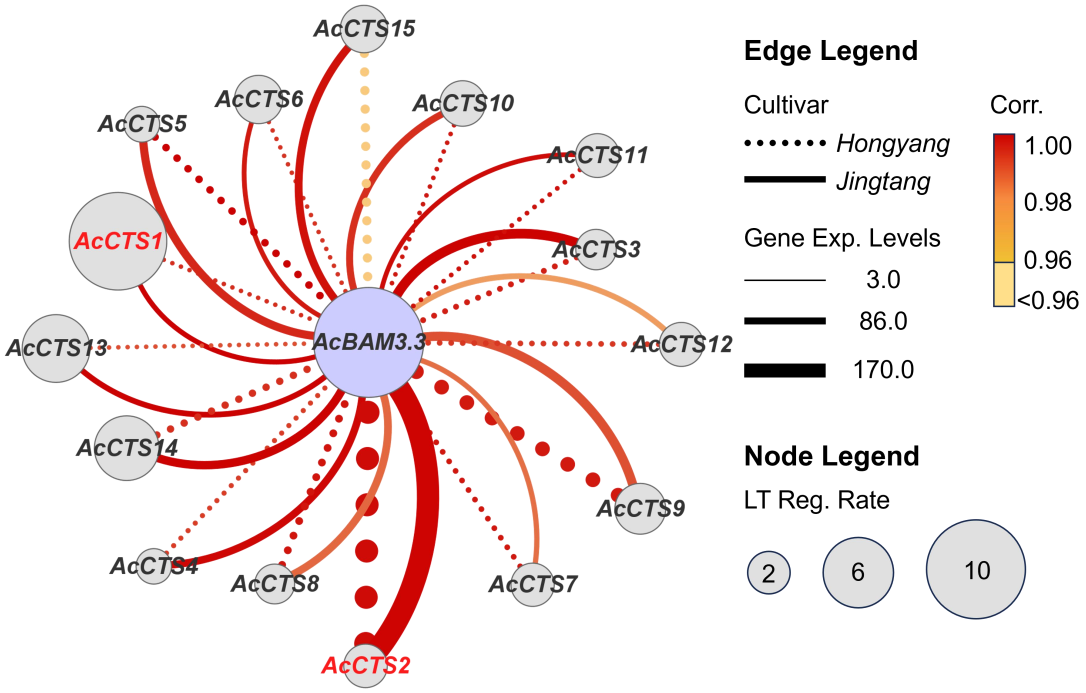

Supplementary Fig. 15 Correlation between *AcBAM3.3* expression and cool temperature-specific (CTS) transcription factors. Threshold: log2FC of CT/ room temperature (RT)>2, R>0.96. AcCTS1 (AcTINY2), Acc12510; AcCTS2 (AcSIG5.1), Acc05562; AcCTS3 (AcbZIP1), Acc23746; AcCTS4 (AcbZIP1), Acc12208; AcCTS5 (AcDBB1), Acc04028; AcCTS6 (AcDof1), Acc06345; AcCTS7 (AcDof2), Acc21930; AcCTS8 (AcDof3), Acc29872; AcCTS9 (AcERF1), Acc02810; AcCTS10 (AcERF2), Acc30992; AcCTS11 (AcMYB1), Acc29660; AcCTS12 (AcMYB2), Acc05338; AcCTS13 (AcNAC1), Acc33448; AcCTS14 (AcNAC2), Acc32998; AcCTS15 (AcNAC3), Acc08011. More detailed information was shown in Data Set S2 and S3.

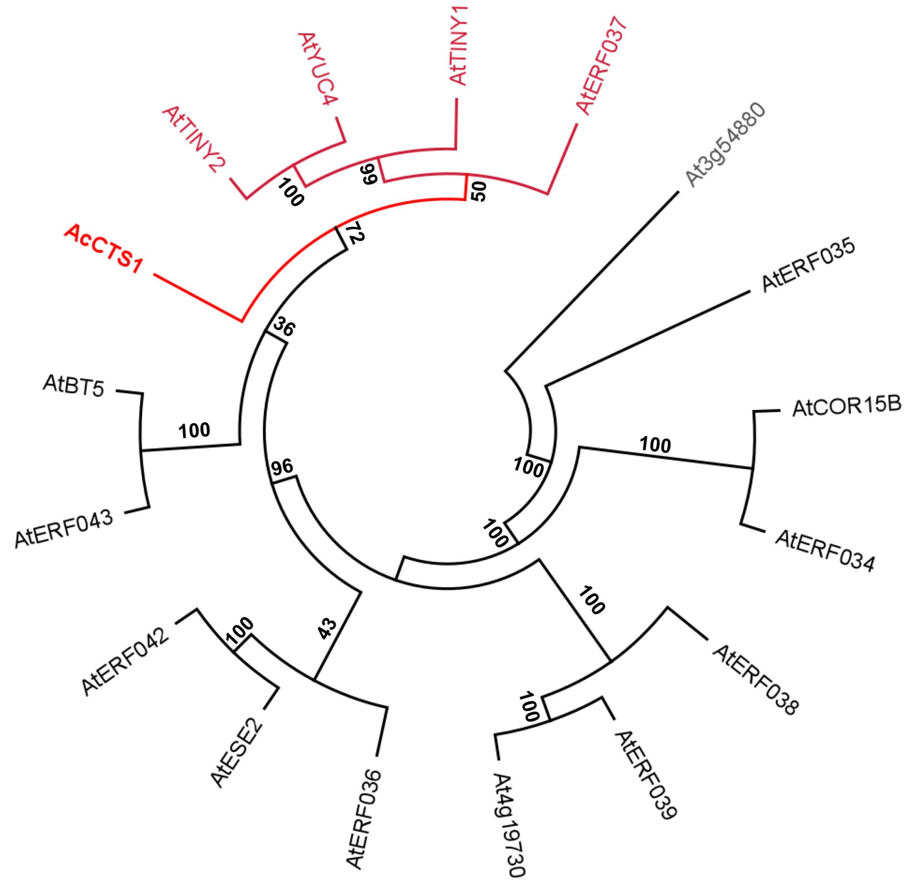

Supplementary Fig. 16 Phylogenetic analysis of amino acid sequences of AcCTS1 and ERFs from *Arabidopsis thaliana*. AcCTS1 and AtTINY2 showed the highest amino acid identity (48.7%). Sequence alignments were conducted using BLASTp in NCBI, with the non-redundant protein sequences (nr) database, limited to Arabidopsis. The remaining settings were left as defaults. Proteins with a BLAST score >130 were chosen for phylogenetic analysis. The phylogenetic tree was constructed using the neighbor-joining method of the MEGA7 software. Bootstrap analysis of 1,000 trials provided a reliable estimate of the topology of phylogenetic tree. The branch number showed the consensus support (%). AcCTS1, Acc12510; AtTINY1, AT5G25810; AtTINY2, AT5G11590; AtERF034, AT2G44940; AtERF035, AT3G60490; AtERF036, AT3G16280; AtERF037, AT1G77200; AtERF038, AT2G35700; AtERF039, AT4G16750; AtERF042, AT2G25820; AtERF043, AT4G32800; AtYUC4, AT5G11320; AtBT5, AT4G37610; AtCOR15B, AT2G42530; AtESE2, AT2G21670.

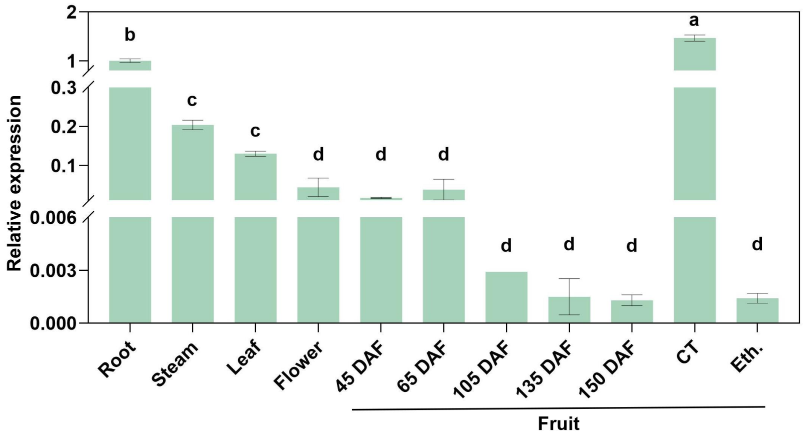

Supplementary Fig. 17 Expression profile of *AcCTS1* in kiwifruit. *AcCTS1* expression was analyzed across different tissues (Root; Stem; Leaf; Flower), fruit developmental stages (45, 65, 105, 135, 150 day after flowering, DAF), and postharvest treatments, including ethylene (Eth.) for 3 days, cool temperature (CT, 5 °C), and room temperature (RT, 20°C) at 15 day after storage (DAS). Data represent mean ± SE (n = 3). Different lowercase letters indicate significant differences ($P < 0.05$).

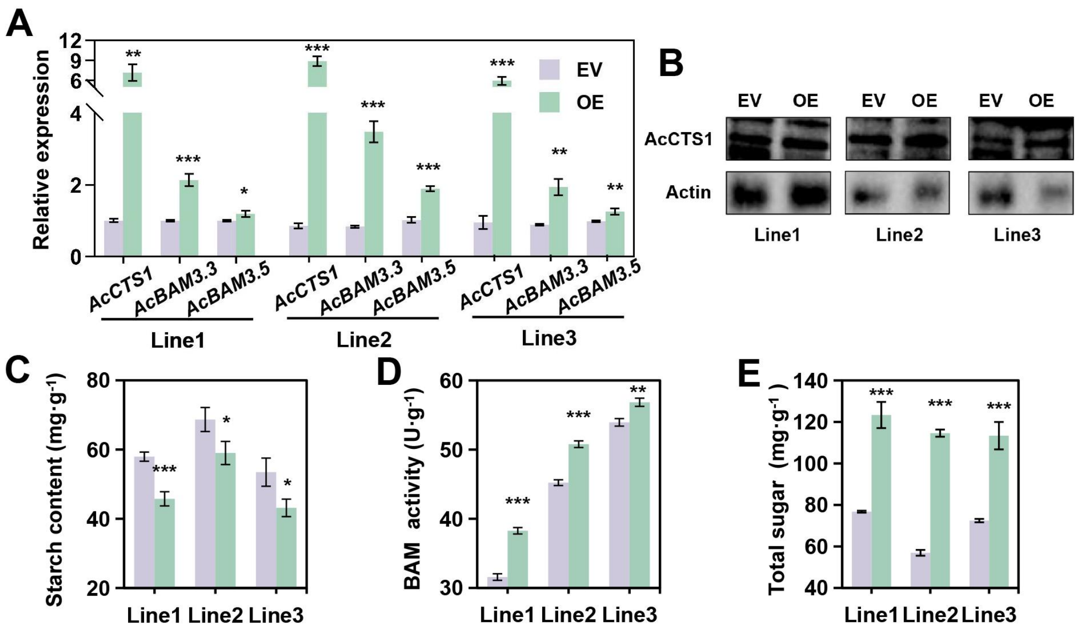

Supplementary Fig. 18 Transient overexpression of *AcCTS1* in kiwifruits enhances CT-induced starch degradation. A) Expression of *AcCTS1*, *AcBAM3.3*, and *AcBAM3.5* at the injection site of kiwifruit. B) Immunoblotting analysis of kiwifruit injected with empty vector and *AcCTS1*-OE. Actin was used as a loading control. C – E) Changes in starch content, BAM activity, and total sugar content in the injection site of kiwifruit. Data are means ± SE from three biological replicates. Asterisks indicate significant differences as determined by Student's *t*-test (*, P<0.05; **, P<0.01; ***, P<0.001).

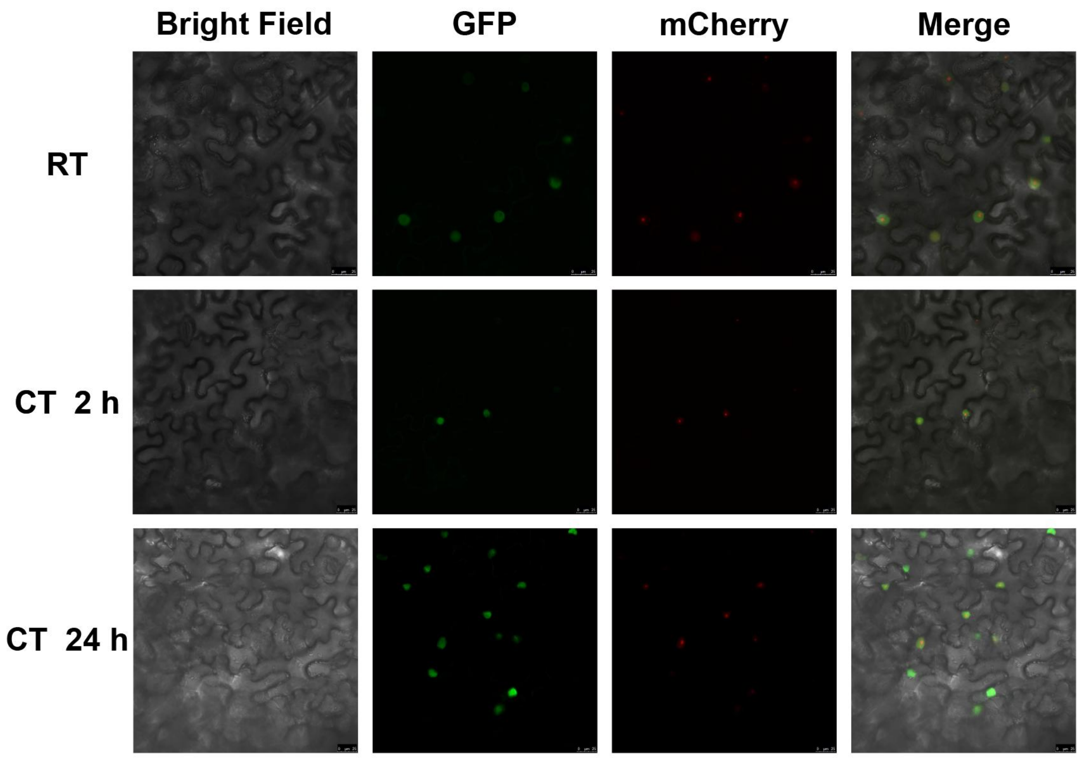

Supplementary Fig. 19 Subcellular localization of AcCTS1. Confocal microscopy images of *N. benthamiana* epidermal cells co-expressing AcCTS1-GFP (green) and the nuclear marker mCherry (red). From left to right: brightfield image, GFP channel, mCherry channel, and merged view. Scale bars are indicated in the bottom right of each panel.

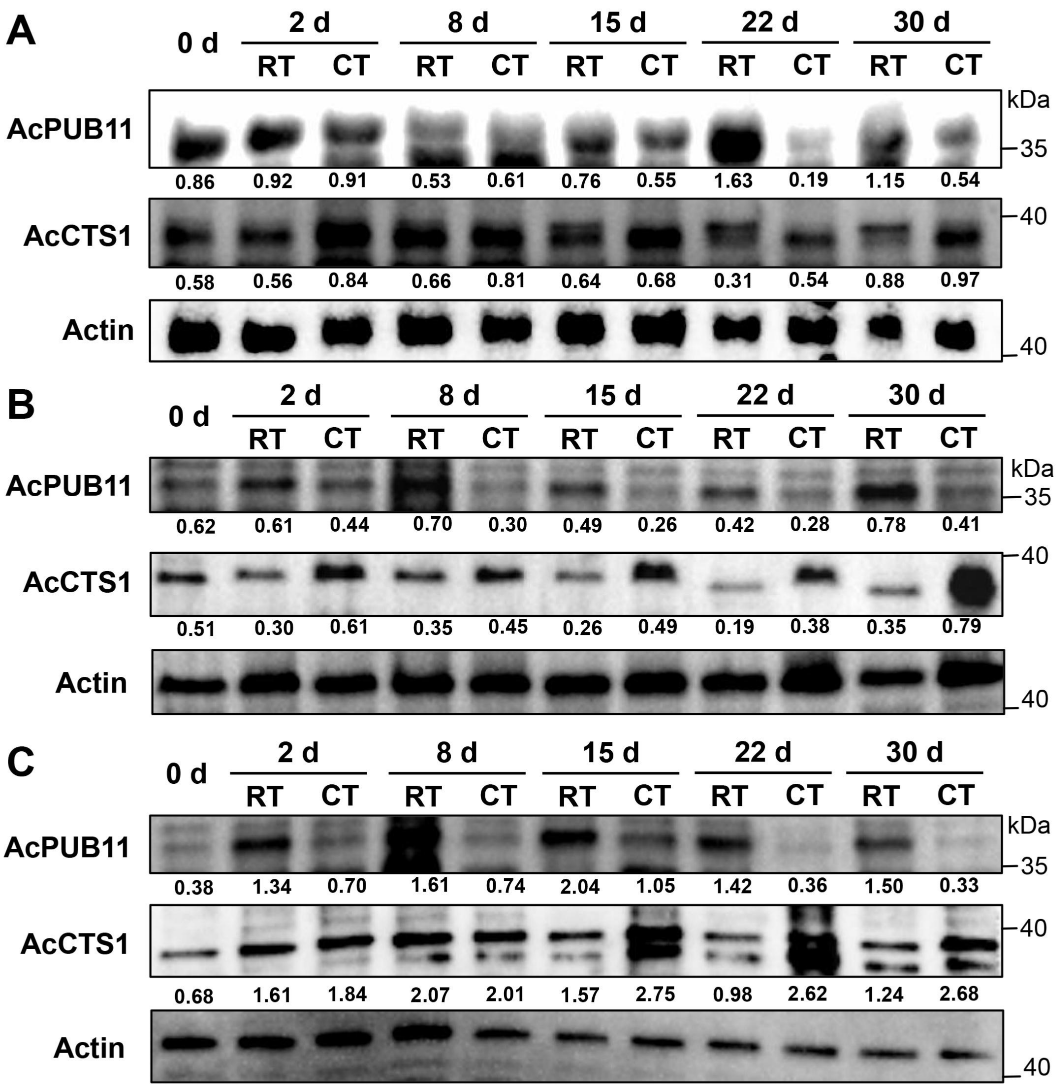

Supplementary Fig. 20 Changes in the abundance of AcPUB11 and AcCTS1 at RT (20°C) and CT (5°C) were assessed using western blot in 'Jintang No.3' (A), 'Hongyang' (B), and 'Jianxiang' (C). The anti-AcPUB11 and anti-AcCTS1 antibodies were used separately, with Actin serving as the loading control. Molecular weight markers (kDa) are shown on the right for each western blot.

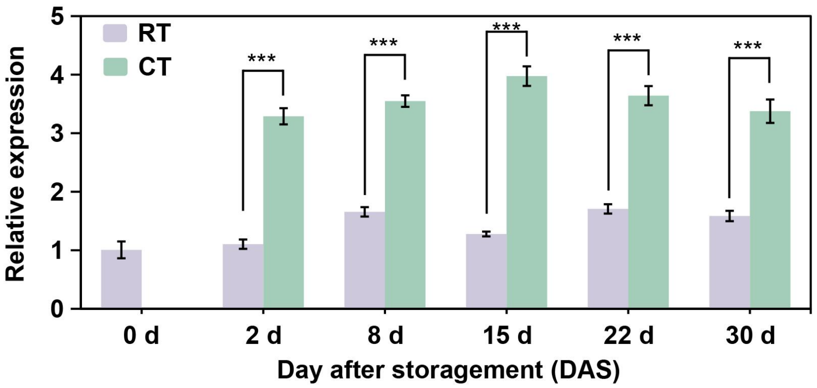

Supplementary Fig. 21 Expression analysis of *AcPUB11* at room temperature (RT) and cool temperature (CT). Relative expression levels of *AcPUB11* mRNA in kiwifruit outer pericarp during storage at RT (20°C) and CT (5°C), as determined by RT-qPCR. Data are mean ± SE (n = 3). Asterisks indicate a significant difference as determined by Student's *t*-test ($P < 0.001$).

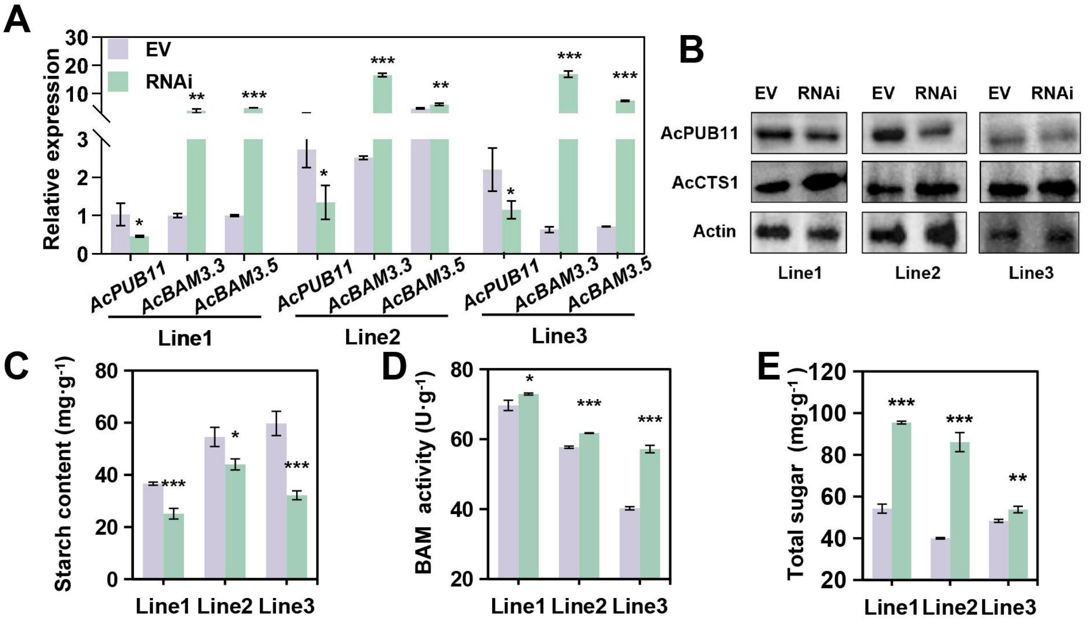

Supplementary Fig. 22 Transient silencing of *AcPUB11* in kiwifruit promotes CT-induced starch degradation. A) Expression of *AcPUB11*, *AcBAM3.3*, and *AcBAM3.5* at the injection site of kiwifruit. B) Immunoblotting analysis of kiwifruit injected with empty vector (EV) and *AcPUB11*-RNAi. Actin was used as a loading control. C–E) Changes in starch content, BAM activity, and total sugar content in the injection site of kiwifruit. Data are means ± SE from three biological replicates. Asterisks indicate significant differences as determined by Student's *t*-test (*, $P<0.05$; **, $P<0.01$; ***, $P<0.001$).

Supplementary Table 1. Genes identified by yeast two-hybrid screening using AcCTS1 as bait

Yeast Clone No.	Gene ID	BLAST identity (%)	Description
2	Acc32202.1	98.91	Kiwellin
3	Acc32202.1	98.29	Kiwellin
4	Acc10675.1	100	YABBY 2
5	0 hits found	\	\
6	Acc17723.1	99.81	Inactive exonuclease EXD1
7	Acc04863.1	95.9	Endochitinase A
8	Acc18864.1	100	Metallothionein-like protein type 3
11	0 hits found	\	\
13	Acc00338.1	97.95	Endochitinase EP3
20	Acc03574.1	99.67	60S ribosomal protein L15
21	Acc21654.1	98.56	Polyubiquitin
22	Acc33269.1	100	40S ribosomal protein S8
25	Acc33269.1	100	40S ribosomal protein S8
26	Acc33269.1	100	40S ribosomal protein S8
27	Acc09233.1	98.91	Plant U-box protein 11
32	Acc00338.1	94.74	Endochitinase EP3
33	Acc20571.1	97.75	Polyubiquitin
34	Acc18864.1	100	Metallothionein-like protein type 3
35	Acc32202.1	99.07	Kiwellin
36	Acc06504.1	96.34	Thebaine 6-O-demethylase
37	Acc33269.1	100	40S ribosomal protein S8
39	Acc21654.1	98.56	Polyubiquitin
44	Acc11113.1	98.8	GTP-binding protein SAR1A
46	Acc27868.1	98.98	Expansin-A8
47	Acc23900.1	99.83	Deubiquitinating enzyme 36
50	Acc23900.1	99.83	Deubiquitinating enzyme 36
56	Acc14525.1	97.07	RING-type E3 ubiquitin transferase
58	Acc32202.1	99.07	Kiwellin
59	Acc11295.1	99.45	Zinc finger protein ZAT10

* Only successfully sequenced yeast clones are listed.

Supplementary Table 2. Primers used in this study

Vector or Gene	Sequence
pKlic1.0-AcCTS1-F	actagttccagggcgcccgggATGAGCACTGAAAGCTGCTCAA
pKlic1.0-AcCTS1-R	atcatcgacccgacgcccgggTCAATAATCCCACAACACAGCC
pKlic1.0-AcCTS2-F	actagttccagggcgcccgggATGGGAGTTGTAACTGTTTCTAGC
pKlic1.0-AcCTS2-R	atcatcgacccgacgcccgggTTAGAAAACGTAACGGCGAAGATAA
pGreen0800II-LUC-AcBAM3.3 promoter-F	ggtggagatcgaattccatgg GGAATGAGCATCCAAATTCATG
pGreen0800II-LUC-AcBAM3.3 promoter-R	tgtttttggcgtcttccatggATGATGTTTCTTCTTGGTTTTGTGA
pGreen0800II-LUC-AcBAM3.5 promoter-F	ggtggagatcgaattccatggTGGCTGAGTTTGTGGGGTTT
pGreen0800II-LUC-AcBAM3.5 promoter-R	tgtttttggcgtcttccatgg ATTCAATTCTTCTGGGTTTTCTGAA
pAbAi-AcBAM3.3 promoter-F	cttgaattcgagctcggtaccGGAATGAGCATCCAAATTCATG
pAbAi-AcBAM3.3 promoter-R	atacagagcacatgcctcgagATGATGTTTCTTCTTGGTTTTGTGA
pGADT7-AcCTS1-F	gccatggaggccagtgaattcATGAGCACTGAAAGCTGCTCAA
pGADT7-AcCTS1-R	cagctcgagctcgatggatccTCAATAATCCCACAACACAGCC
pGADT7-AcPUB11-F	gccatggaggccagtgaattcTCATGGAAGTGAAGCATCGGACG
pGADT7-AcPUB11-R	cagctcgagctcgatggatccTTATGAAGAGCGACTTGATAAAGAA
pK7GW35s-AcBAM3.1-F	gtacaaaaaagcaggcttcATGGCTTTGACGCTACGTT
pK7GW35s-AcBAM3.1-R	tgtacaagaaagctgggtccccCACTAGAGCGGCCTTCTTC
pK7GW35s-AcBAM3.2-F	gtacaaaaaagcaggcttcATGGCTTTGACGCTACGTT
pK7GW35s-AcBAM3.2-R	tgtacaagaaagctgggtccccTGATTTTGATCCCAGTAAACTAATA
pK7GW35s-AcBAM3.3-F	gtacaaaaaagcaggcttcATGGCTTTGACGCTACGCTC
pK7GW35s-AcBAM3.3-R	tgtacaagaaagctgggtccccCACTAGAGCAGCCTCCTTC
pK7GW35s-AcBAM3.5-F	gtacaaaaaagcaggcttcATGGCTTTAACATTACATTGTTCGA
pK7GW35s-AcBAM3.5-R	tgtacaagaaagctgggtccccCACAAAAGCAGCCTCCTTG
pK7GW35s-AcCTS1-F	gtacaaaaaagcaggcttcATGAGCACTGAAAGCTGCTCAA
pK7GW35s-AcCTS1-R	tgtacaagaaagctgggtccccATAATCCCACAACACAGCCCC
ph7lic -AcPUB11-F （GFP-AcPUB11）	attacgccgaggTCATGGAAGTGAAGCATCGGACG
ph7lic -AcPUB11-R （GFP-AcPUB11）	tagggaagaggTTATGAAGAGCGACTTGATAAAGAA
pHELLSGATE 8-AcCTS1-F	gtacaaaaaagcaggcttcATGAGCACTGAAAGCTGCTCAA
pHELLSGATE 8-AcCTS1-R	tgtacaagaaagctgggtccccTTCGGGGAAGTTGAGCACAG
pHELLSGATE 8-AcPUB11-F	gtacaaaaaagcaggcttcATGGAAGTGAAGCATCGGACG
pHELLSGATE 8-AcPUB11-R	tgtacaagaaagctgggtccccGGGGTTGAGGAGGGCGTG
pGBKT7-AcCTS1-F	atggccatggaggccgaattcATGAGCACTGAAAGCTGCTCAA
pGBKT7-AcCTS1-R	ccgctgcaggtcgacggatccTCAATAATCCCACAACACAGCC
pGEX-4T-1-AcPUB11-F	atcggatctggttccgcgtggatccATGGAAGTGAAGCATCGGACG
pGEX-4T-1-AcPUB11-R	gctcgagtcgacccgggaattcTTATGAAGAGCGACTTGATAAAGAA
pMAL-C6T-AcCTS1-F	tccagatgctgatgggcggccgcATGAGCACTGAAAGCTGCTCAA
pMAL-C6T-AcCTS1-R	acctgcagggaattcggatccTCAATAATCCCACAACACAGCC
ph7lic-AcCTS1-F (HA-AcCTS1)	attacgccgaggTCATGAGCACTGAAAGCTGCTCAA
ph7lic-AcCTS1-R (HA-AcCTS1)	tagggaagaggTCAATAATCCCACAACACAGCC
ph7lic-GUS-F（GFP-GUS）	attacgccgaggTCATGTTACGTCCTGTAGAAACCC
ph7lic-GUS-R（GFP-GUS）	tagggaagaggTCATTGTTTGCCTCCCTGCT
pMDC43-nYFP-AcCTS1-F	gtacaaaaaagcaggcttcATGAGCACTGAAAGCTGCTCAA
pMDC43-nYFP-AcCTS1-R	tgtacaagaaagctgggtccccTCAATAATCCCACAACACAGCC
pMDC43-cYFP-AcPUB11-F	gtacaaaaaagcaggcttcATGGAAGTGAAGCATCGGACG
pMDC43-cYFP-AcPUB11-R	tgtacaagaaagctgggtccccTTATGAAGAGCGACTTGATAAAGAA
pCDFD-AcCTS1-F	gagggaaggatttcagaattcATGAGCACTGAAAGCTGCTCAA
pCDFD-AcCTS1-R	aacgtcgtatgggtaaggcctATAATCCCACAACACAGCCCC
pACYCD-AcPUB11-F	gcatcaccatcatcaccaagccagggatccATGGAAGTGAAGCATCGGA
pACYCD-AcPUB11-R	cctcggagatgagcttctgctcaggcctTGAAGAGCGACTTGATAAAGAA [illegible]
BAM3.3 promoter-F (FAM labeled, EMSA)	AAAACTACACTTGCCGACTACAAATCCTCT
BAM3.3 promoter-F (Cold probe,EMSA)	AAAACTACACTTGCCGACTACAAATCCTCT
BAM3.3 promoter-R (EMSA)	AGAGGATTTGTAGTCGGCAAGTGTAGTTTT
BAM3.3 promoter-F (Mutant probe, EMSA)	AAAACTACACTTAAAAAATACAAATCCTCT
BAM3.3 promoter-R (Mutant probe, EMSA)	AGAGGATTTGTATTTTTTAAGTGTAGTTTT
BAM3.5 promoter-F (FAM labeled, EMSA)	TTCAGAAATAGCTCCGACGGCCGTTCTCAC
BAM3.5 promoter-F (Cold probe,EMSA)	TTCAGAAATAGCTCCGACGGCCGTTCTCAC
BAM3.5 promoter-R (EMSA)	GTGAGAACGGCCGTCGGAGCTATTTCTGAA

Vector or Gene	Sequence
BAM3.5 promoter-F (Mutant probe, EMSA)	TTCAGAAATAGCTAAAAAGGCCGTTCTCAC
BAM3.5 promoter-R (Mutant probe, EMSA)	GTGAGAACGGCCTTTTTAGCTATTTCTGAA
AcCTS1-F (Hi-TOM)	CTGAAAGCTGCTCAAACTCG
AcCTS1-R (Hi-TOM)	GATCCGTTTGATTTTCGTCAGG
AcPUB11-F (Hi-TOM)	AGACAGCGAATTGGAGATGG
AcPUB11-R (Hi-TOM)	ATATGAGACGAAGCTCGCAG
Actin-F (RT-qPC)	TGCATGAGCGATCAAGTTTCAAG
Actin-R (RT-qPC)	TGTCCCATGTCTGGTTGATGACT
AcCTS1-F (RT-qPC)	CGAGTTACGAATCCAACGAGTC
AcCTS1-R (RT-qPC)	CAGCCCCAAAACTGCTTTG
AcPUB11-F (RT-qPC)	GTTCCTGCTTTGTTCTCGTTG
AcPUB11-R (RT-qPC)	ATAACCCCAATACCCGAAACC
AcBAM3.3-F (RT-qPC)	CCGAATACCTATCCTTGGCTTG
AcBAM3.3-R (RT-qPC)	GAATTTCCGCAATAACCTCGC
AcBAM3.5-F (RT-qPC)	GGAACTGGAGCTAAACTATCTGG
AcBAM3.5-R (RT-qPC)	TCCATGTTTACCCAGTGTACG
